# Supplementary material for: Self-organization of plasticity and specialization in a primitively social insect
Source: Cell Syst. 2022 Sep 21;13(9):768–779.e4. doi: 10.1016/j.cels.2022.08.002 (PMC9512265; doi:10.1016/j.cels.2022.08.002)
Supplement: Document S1. Figures S1–S5, Tables S1–S3, supplemental theory, and supplemental references [file mmc1.pdf]

Cell Systems, Volume *13*

Supplemental information

Self-organization of plasticity

and specialization in a primitively social insect

Solenn Patalano, Adolfo Alsina, Carlos Gregorio-Rodríguez, Martin Bachman, Stephanie Dreier, Irene Hernando-Herraez, Paulin Nana, Shankar Balasubramanian, Seirian Sumner, Wolf Reik, and Steffen Rulands

Supplementary Information for:

“Self-organisation of plasticity and specialization in a primitively social insect”

Contents:

Supplementary Figures

Supplementary Tables

Supplemental Theory

Supplementary Figure 1

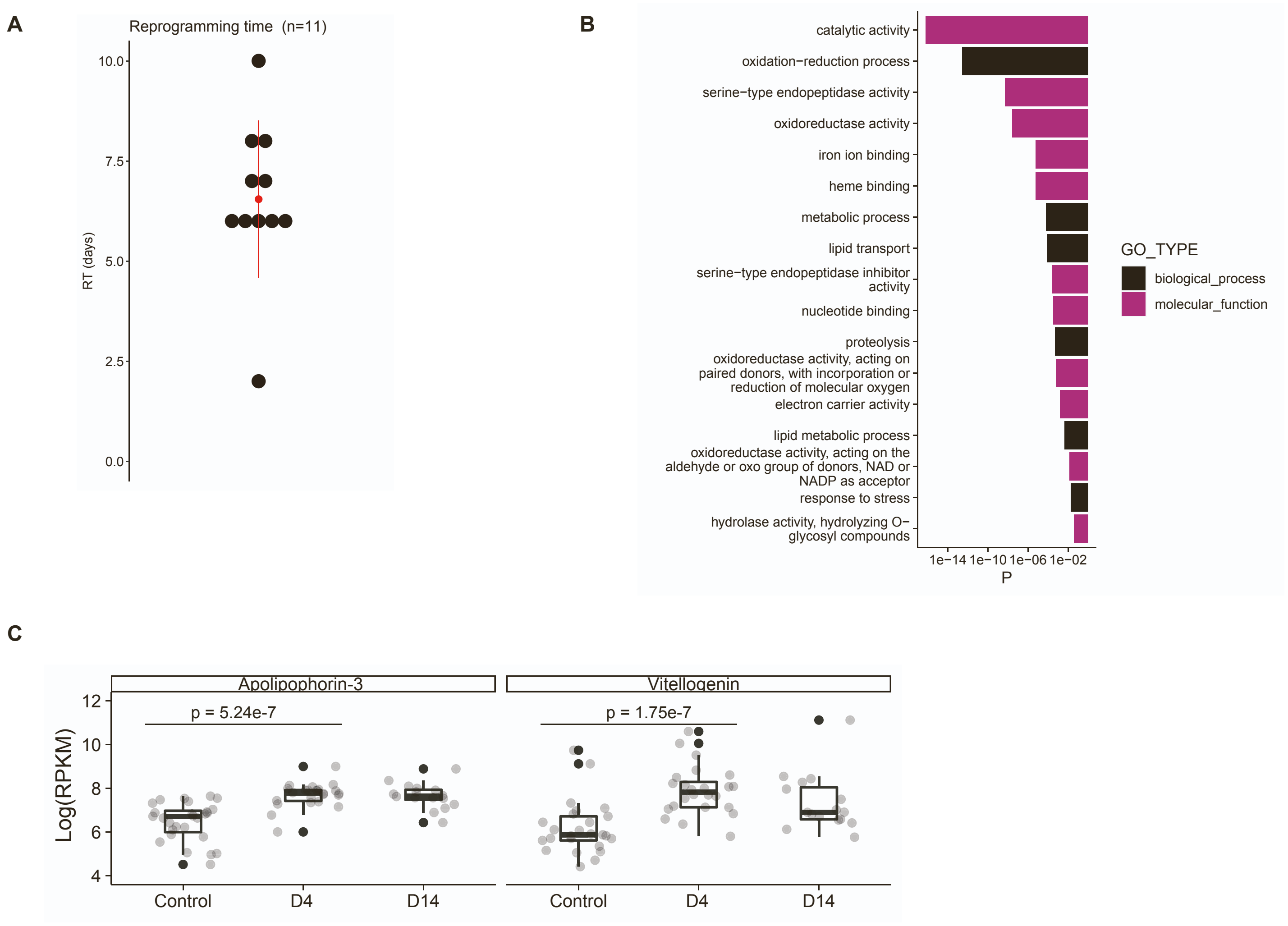

Supplementary Fig. 1.

A, Reprogramming times (i.e. the time taken for a new egglayer to emerge) after queen removal for individual nests (dots). Mean and standard deviation are represented by crossbars. **B,** GO enrichment analysis of the 227 differentially expressed genes. **C,** Expression levels of Vitellogenin and Apolipophorin-3 across the 3 phases (Control, D4, D14). P-values in **b** and **c** were calculated using the R package DESeq2 and corrected for multiple testing using the Benjamini-Hochberg method.

Supplementary Figure 2

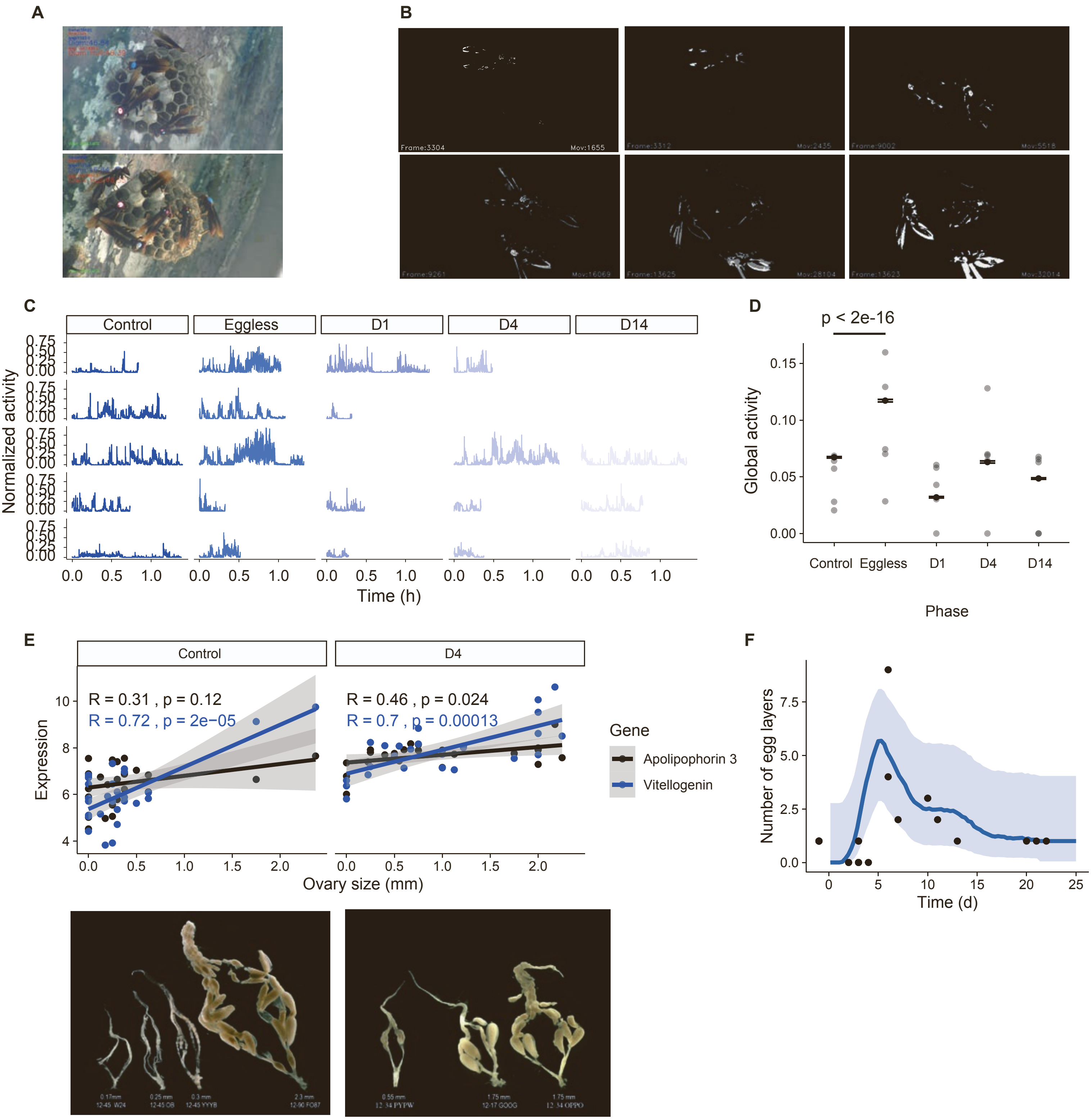

Supplementary Fig. 2.

A, Normalisation using the tag diameter to adjust camera and nest distance. **B,** Example of an increase in global movement detection in the nest N1217 shown by the increase of the amount of white at different time points of the analysis of pixel changes across various frames. 'Frame' indicates the time in which the picture was taken. 'Mov' indicates the number of pixels that changed compared with the previous frame. **C,** Global activity for each nest over time. **D,** Global changes in nest activity. Each grey dot represents a nest. Black dots and error bars correspond to the mean and SEM of all frames. A t-test was performed on the null hypothesis that average activity is equal in control and reprogrammed nests. **E,** Pearson correlation between ovaries development and both *Vitellogenin* and *Apolipophorin-3* genes. Examples of specific ovary dissections for each phase are shown below. **F,** Predicted and measured number of egg layers (ovary size > 1.5mm) over time after queen removal. Shaded area denotes 95% confidence bounds of the theoretical prediction for individual nests.

Supplementary Figure 3

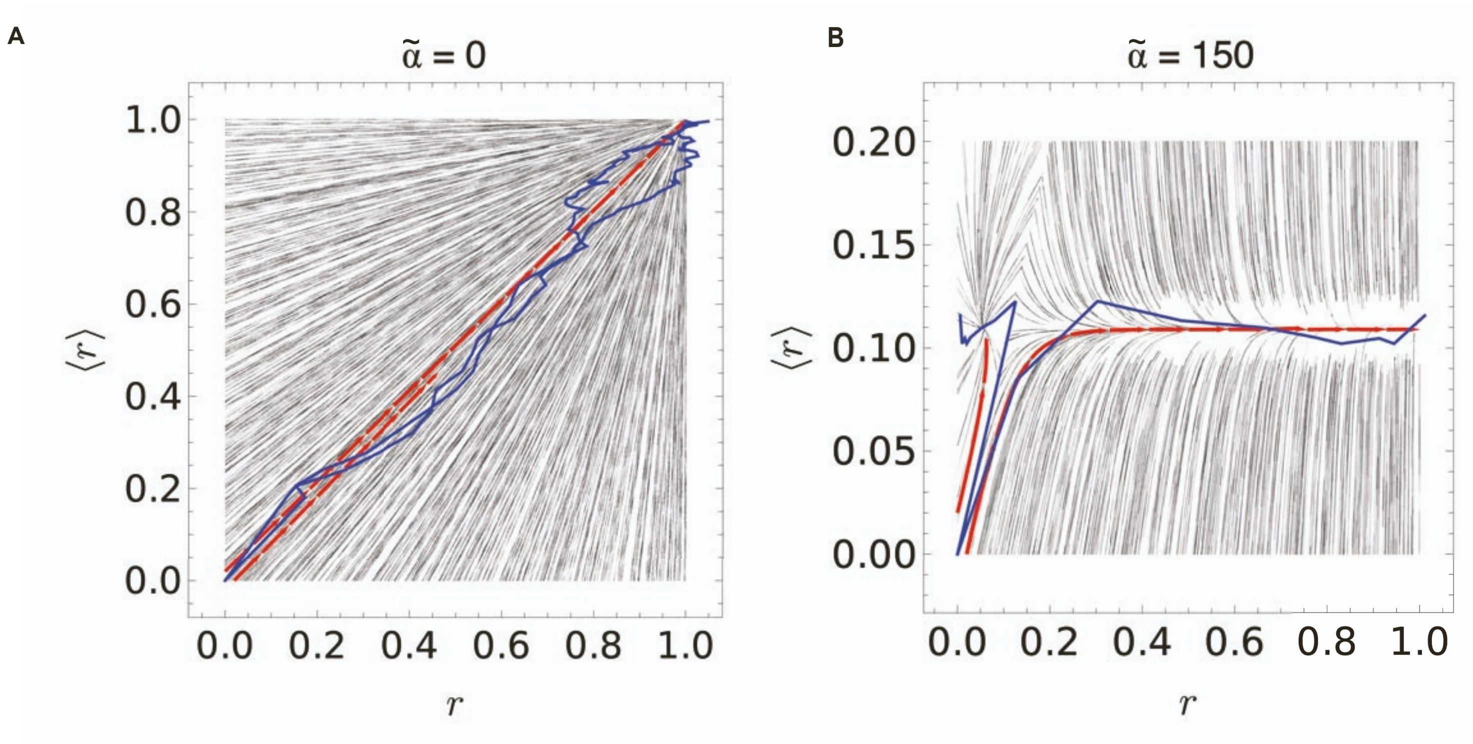

Supplementary Fig. 3.

Phase portrait of the mean-field master equation for values of the interaction rate $\tilde{\alpha}$ corresponding to fully reproductive (**A**) and social **(B)** steady states. The flow lines of the vector field defined by the mean-field master equation are represented by grey lines and two trajectories originating around the origin, corresponding to a nest composed only of individuals lacking queen gene expression, are highlighted in red. Blue lines denote trajectories from numerical simulations of the full stochastic description of the system (Supplemental Theory).

Supplementary Figure 4

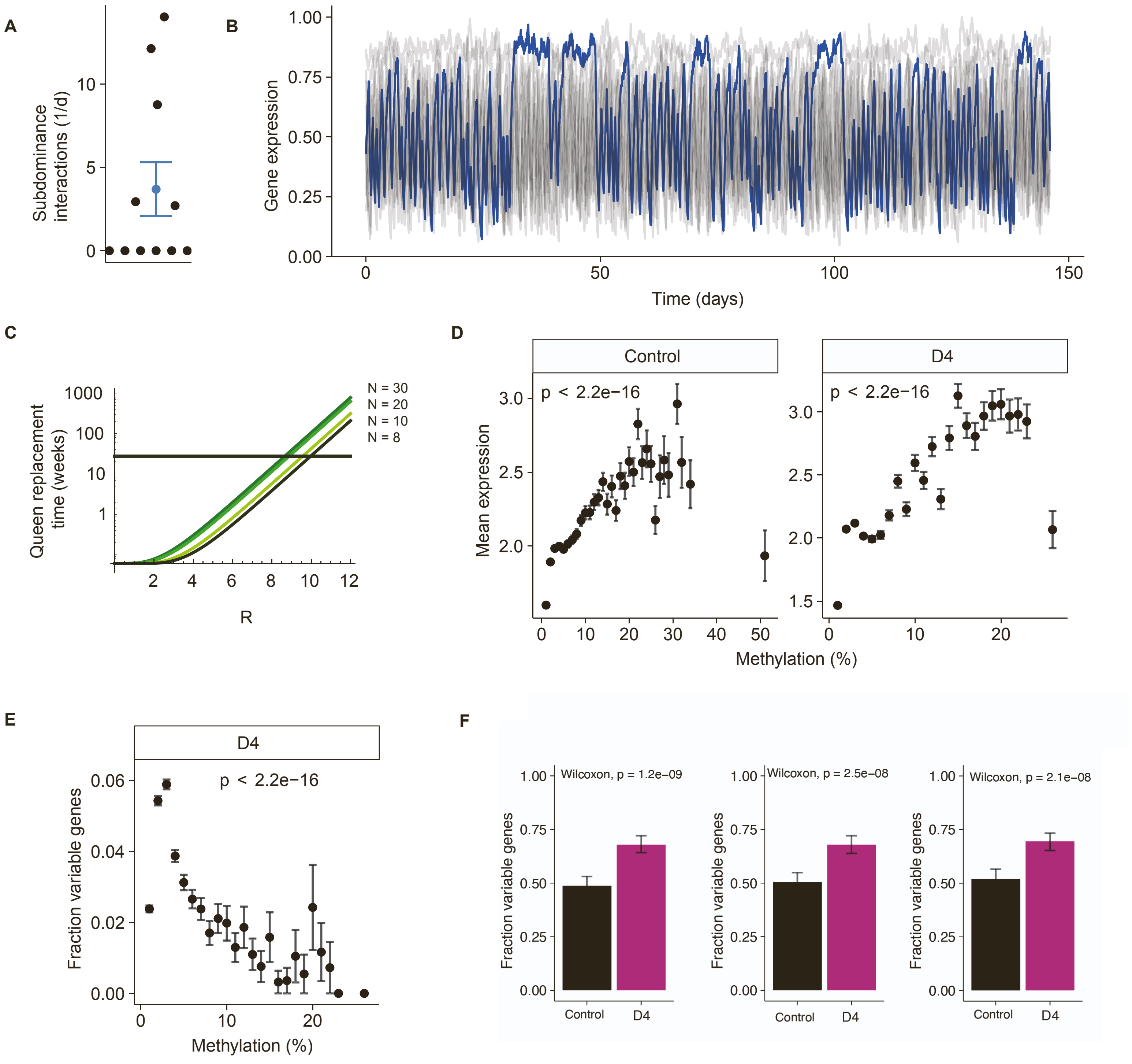

Supplementary Fig. 4.

A, Rate of subdominant interactions per worker and per hour and normalised by the total number of individuals detected during night census (see Methods section). **B,** Sample trajectory obtained from numerical simulations showing the stochastic turnover of queens**. C,** Predicted queen replacement times as a function of the ratio R between interaction and molecular time scales for different values of the population size N. The horizontal line indicates the experimentally measured queen lifespan in *Polistes canadensis* (Supplementary Table 1). **D,** Mean gene expression as a function of the DNA methylation levels in gene bodies. P-values were determined from a Pearson correlation test. **E,** Fraction of significantly variable genes as a function of DNA methylation in the same genes in the early-commitment phase. The p-value was determined from a Pearson correlation test. **F,** Fraction of significantly variable genes in control and early commitment phase for different threshold values of the adjusted p-value: 0.01 (left), 0.05 (centre), 0.1 (right).

Supplementary Figure 5

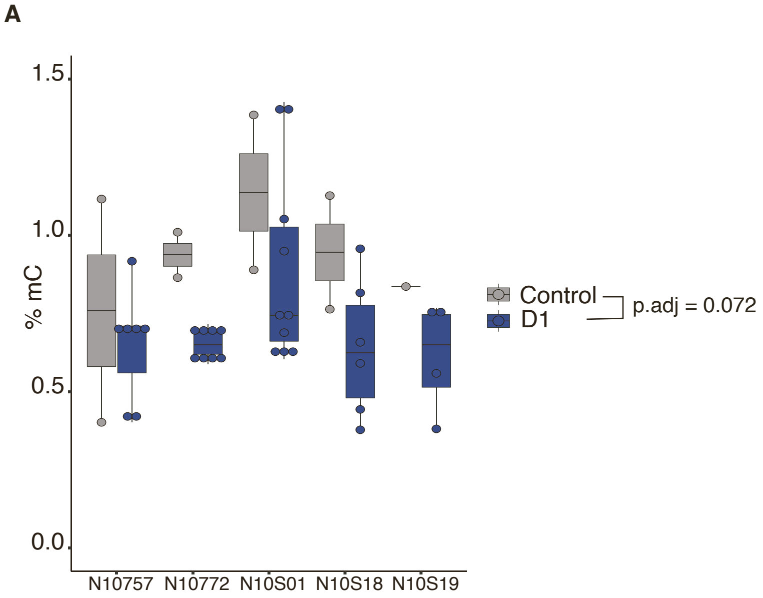

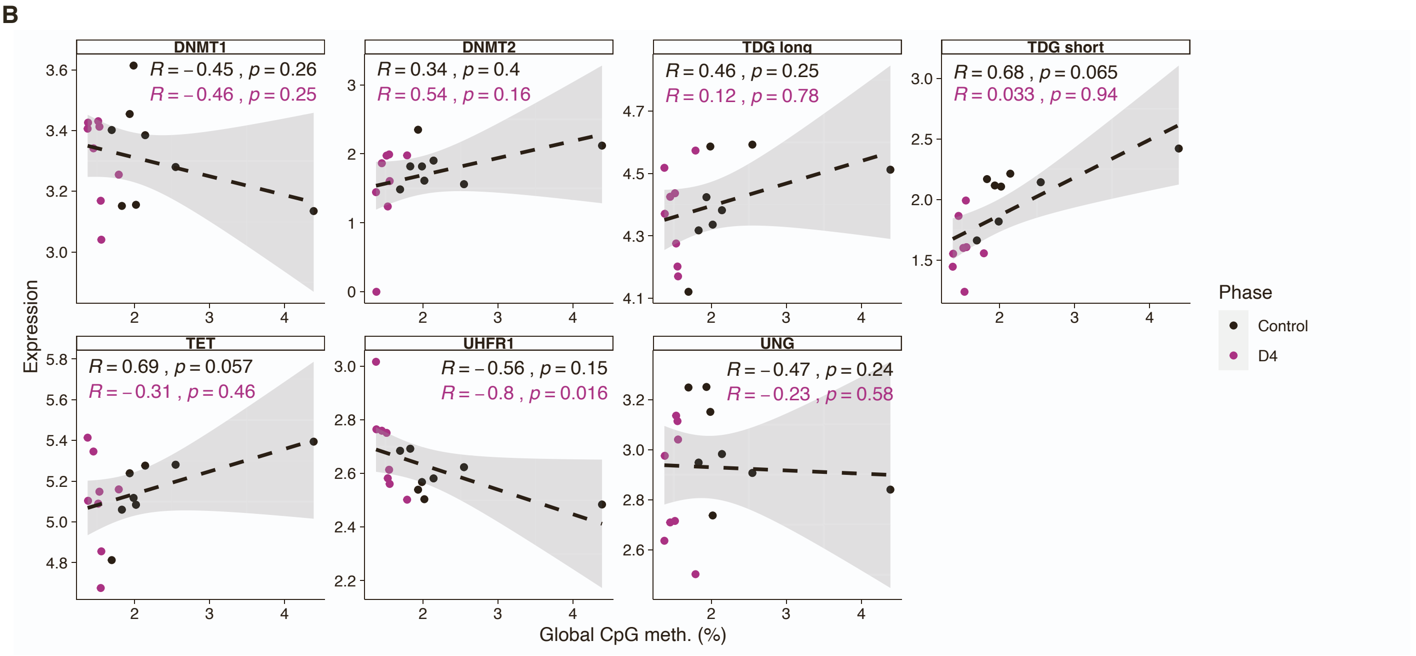

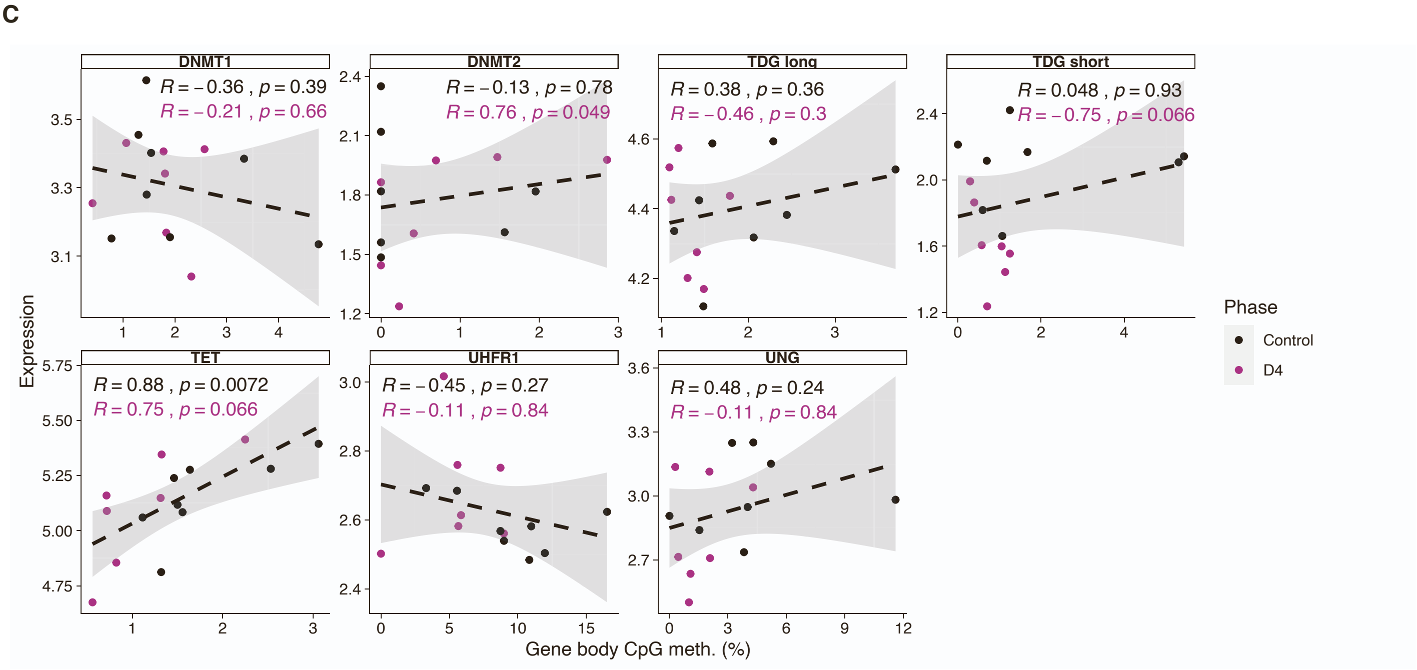

Supplementary Fig. 5.

A, Global level of DNA methylation measured by mass spectrometry in *Polistes canadensis* before and after queen removal (n=44). Center line corresponds to the median and lower and upper hinges to the 25th and 75th percentiles, respectively. P-value was calculated from a Wilcoxon signed rank test between the corresponding phases and corrected for multiple testing using the Holm method. **B, C,** Gene expression of key members of DNA methylation enzymatic machinery[19] as a function of global DNA methylation (B) and gene body DNA methylation (C) in the same genes. R and p denote Spearman's rank correlation coefficient and p-value, respectively, calculated independently for each of the two phases.

Supplementary Tables

Supplementary Table 1.

Number of collected and analysed individuals for each phase of queen removal experiments

Nest	Experiment	Collection	Individual collected	Behavioural - observation	Ovaries dissection	Mass spec methyl	RNA-seq sequencing	BS-seq sequencing
N10789	Control	8-Jul-10	14	N/A	13	0	0	0
N1253	Control	2-May-12	17	N/A	17	0	0	0
N1239	Control	8-May-12	21	N/A	21	0	0	0
N1219	Control	9-May-12	17	N/A	16	0	0	0
N1245	Control	18-May-12	16	N/A	16	0	16	0
N1255	Control	17-Jun-12	12	N/A	12	0	0	0
N1290	Control	17-Jun-12	12	N/A	11	0	11	8
N12101	Control	17-Jun-12	5	N/A	5	5	0	0
N09108	Eggless	20-Jul-09	16	N/A	16	0	0	0
N09143	Eggless	22-Jul-09	12	N/A	12	0	0	0
N09145	Eggless	23-Jul-09	21	N/A	21	0	0	0
N09175	Eggless	17-Jul-09	23	N/A	23	0	0	0
N09178	Eggless	17-Jul-09	17	N/A	15	0	0	0
N09230	Eggless	23-Jul-09	14	N/A	14	0	0	0
N09302	Eggless	23-Jul-09	13	N/A	12	0	0	0
N10757	Control / D1	28-Jun-10	14	N/A	13	9	0	0
N10772	Control / D1	7-Jul-10	45	N/A	43	10	0	0
N10S01	Control / D1	7-Jul-10	15	N/A	14	14	0	0
N10S18	Control / D1	7-Jul-10	10	N/A	10	8	0	0
N10S19	Control / D1	7-Jul-10	6	N/A	6	5	0	0

N1217	D4	27-May-12	10	18	10	0	8	8
N1234	D4	9-Jun-12	13	12	11	0	8	0
N1279	D4	7-Jun-12	11	N/A	10	0	8	0
N1242	D14	9-Jun-12	18	14	18	0	16	0
N1265	D14	13-Jun-12	11	15	11	0	9*	0
N1283	D14	9-Jun-12	15	21	15	0	11*	0
**Total**			**398**	**80**	**385**	**51**	**87**	**16**

* Unoriented sequenced libraries

Supplementary Table 2.

Sequencing data and their respective NCBI SRA accession numbers.

Nest	Experiment	Tag ID	Phenotype	Brain Dissection ID	Sequencing	BioSample	SRA
N1245	Control	OOOW	Worker	P98	RNA-seq	SAMN13938101	SRX7644456
N1245	Control	White	Worker	P99	RNA-seq	SAMN13938025	SRX7644457
N1245	Control	BYBY	Worker	P100	RNA-seq	SAMN13938077	SRX7644382
N1245	Control	W16	Foundress	P101	RNA-seq	SAMN13938076	SRX7644383
N1245	Control	GGGO	Worker	P102	RNA-seq	SAMN13938075	SRX7644384
N1245	Control	W23	Foundress	P103	RNA-seq	SAMN13938074	SRX7644385
N1245	Control	BOO	Worker	P104	RNA-seq	SAMN13938073	SRX7644386
N1245	Control	W64 - Pink	Worker	P105	RNA-seq	SAMN13938043	SRX7644387
N1245	Control	W24	Foundress	P106	RNA-seq	SAMN13938042	SRX7644388
N1245	Control	OB	Worker	P107	RNA-seq	SAMN13938041	SRX7644389
N1245	Control	W19	Queen	P108	RNA-seq	SAMN13938023	SRX7644390
N1245	Control	OBW	Worker	P109	RNA-seq	SAMN13938022	SRX7644391
N1245	Control	GYP	Worker	P110	RNA-seq	SAMN13938021	SRX7644392
N1245	Control	YYYB	Worker	P111	RNA-seq	SAMN13938020	SRX7644393
N1245	Control	W67	Foundress	P112	RNA-seq	SAMN13938019	SRX7644394
N1245	Control	W51 - Pink	Foundress	P113	RNA-seq	SAMN13938018	SRX7644395
N1290	Control	OOBG	Worker	P127	BS-seq	SAMN13938017	SRX7644365

N1290	Control	BPPG	Worker	P128	BS-seq	SAMN13938016	SRX7644366
N1290	Control	PBYY	Worker	P129	BS-seq	SAMN13938015	SRX7644367
N1290	Control	WYBG	Worker	P130	BS-seq	SAMN13938014	SRX7644368
N1290	Control	FO91	Foundress	P131	BS-seq	SAMN13938013	SRX7644369
N1290	Control	OOYG	Worker	P132	BS-seq	SAMN13938012	SRX7644370
N1290	Control	OWGB	Worker	P133	BS-seq	SAMN13938011	SRX7644371
N1290	Control	FO87	Queen	P134	BS-seq	SAMN13938010	SRX7644372
N1290	Control	OOBG	Worker	P127	RNA-seq	SAMEA2061704	ERS227149
N1290	Control	BPPG	Worker	P128	RNA-seq	SAMEA2060493	ERS227150
N1290	Control	PBYY	Worker	P129	RNA-seq	SAMEA2060662	ERS227151
N1290	Control	WYBG	Worker	P130	RNA-seq	SAMEA2061705	ERS227152
N1290	Control	FO91	Foundress	P131	RNA-seq	SAMEA2060660	ERS227145
N1290	Control	OOYG	Worker	P132	RNA-seq	SAMEA2060492	ERS227147
N1290	Control	OWGB	Worker	P133	RNA-seq	SAMEA2060661	ERS227148
N1290	Control	FO87	Queen	P134	RNA-seq	SAMEA2060491	ERS227144
N1290	Control	BPPY	Worker	P135	RNA-seq	SAMEA2060663	ERS227154
N1290	Control	BWWP	Worker	P136	RNA-seq	SAMEA2060494	ERS227153
N1290	Control	PYYY	Worker	P137	RNA-seq	SAMEA1971113	ERS227146
N1217	D4	P91	Foundress	P138	BS-seq	SAMN13938009	SRX7644373
N1217	D4	GYPG	Worker	P141	BS-seq	SAMN13938008	SRX7644374
N1217	D4	GOOG	Worker	P142	BS-seq	SAMN13938007	SRX7644375

N1217	D4	YGGG	Egg Layer	P143	BS-seq	SAMN13938006	SRX7644376
N1217	D4	OWPP	Worker	P144	BS-seq	SAMN13938005	SRX7644377
N1217	D4	P72 (From 12-18)	Foundress	P145	BS-seq	SAMN13938004	SRX7644378
N1217	D4	BPBP	Egg Layer	P146	BS-seq	SAMN13938003	SRX7644379
N1217	D4	Y65 - PPWW	Worker	P147	BS-seq	SAMN13938078	SRX7644380
N1217	D4	P91	Foundress	P138	RNA-seq	SAMN13938040	SRX7644396
N1217	D4	GYPG	Worker	P141	RNA-seq	SAMN13938039	SRX7644397
N1217	D4	GOOG	Worker	P142	RNA-seq	SAMN13938038	SRX7644398
N1217	D4	YGGG	Egg Layer	P143	RNA-seq	SAMN13938037	SRX7644399
N1217	D4	OWPP	Worker	P144	RNA-seq	SAMN13938036	SRX7644400
N1217	D4	P72 (From 12-18)	Foundress	P145	RNA-seq	SAMN13938035	SRX7644401
N1217	D4	BPBP	Egg Layer	P146	RNA-seq	SAMN13938034	SRX7644402
N1217	D4	Y65 - PPWW	Worker	P147	RNA-seq	SAMN13938033	SRX7644403
N1234	D4	OPYW	Worker	P148	RNA-seq	SAMN13938032	SRX7644404
N1234	D4	YOOG	Worker	P149	RNA-seq	SAMN13938031	SRX7644405
N1234	D4	OPPO	Egg layer	P150	RNA-seq	SAMN13938030	SRX7644406
N1234	D4	BPPB	Worker	P151	RNA-seq	SAMN13938029	SRX7644407
N1234	D4	WBYB	Worker	P152	RNA-seq	SAMN13938028	SRX7644408
N1234	D4	YGPP	Worker	P153	RNA-seq	SAMN13938027	SRX7644409
N1234	D4	YYYG	Worker	154	RNA-seq	SAMN13938026	SRX7644410
N1234	D4	PYPW	Worker	P157	RNA-seq	SAMN13938100	SRX7644411

N1279	D4	PBBP	Worker	P160	RNA-seq	SAMN13938099	SRX7644412
N1279	D4	Y11	Egg layer	P161	RNA-seq	SAMN13938098	SRX7644413
N1279	D4	BWB	Worker	P162	RNA-seq	SAMN13938097	SRX7644414
N1279	D4	BGYY=YBYW	Worker	P163	RNA-seq	SAMN13938096	SRX7644415
N1279	D4	PWG	Egg layer	P164	RNA-seq	SAMN13938095	SRX7644416
N1279	D4	PPPP	Worker	P165	RNA-seq	SAMN13938094	SRX7644417
N1279	D4	YWOO	Worker	P166	RNA-seq	SAMN13938093	SRX7644418
N1279	D4	BYBP	Worker	P167	RNA-seq	SAMN13938092	SRX7644419
N1242	D14	Bl	Worker	P172	RNA-seq	SAMN13938091	SRX7644420
N1242	D14	OGBB	Worker	P173	RNA-seq	SAMN13938090	SRX7644421
N1242	D14	YPGP	Worker	P174	RNA-seq	SAMN13938089	SRX7644422
N1242	D14	B59 - BP	Egg layer	P175	RNA-seq	SAMN13938088	SRX7644423
N1242	D14	OOBO	Worker	P176	RNA-seq	SAMN13938087	SRX7644424
N1242	D14	WYGY	Worker	P177	RNA-seq	SAMN13938086	SRX7644425
N1242	D14	WGBB	Worker	P178	RNA-seq	SAMN13938085	SRX7644426
N1242	D14	B10	Foundress	P179	RNA-seq	SAMN13938084	SRX7644427
N1242	D14	WYOY	Worker	P180	RNA-seq	SAMN13938083	SRX7644428
N1242	D14	YWOY	Worker	P181	RNA-seq	SAMN13938082	SRX7644429
N1242	D14	OYOY	Worker	P182	RNA-seq	SAMN13938081	SRX7644430
N1242	D14	W71	Worker	P183	RNA-seq	SAMN13938080	SRX7644431
N1242	D14	OPWO	Worker	P184	RNA-seq	SAMN13938079	SRX7644432

N1242	D14	PPBG	Worker	P185	RNA-seq	SAMN13938123	SRX7644433
N1242	D14	YOY	Worker	P186	RNA-seq	SAMN13938122	SRX7644434
N1242	D14	Bl	Worker	P187	RNA-seq	SAMN13938121	SRX7644435
N1265	D14	BWOB	Worker	P188	RNA-seq	SAMN13938120	SRX7644436
N1265	D14	BGGP	Worker	P189	RNA-seq	SAMN13938119	SRX7644437
N1265	D14	White spot	Foundress	P190	RNA-seq	SAMN13938118	SRX7644438
N1265	D14	WGPP	Worker	P191	RNA-seq	SAMN13938117	SRX7644439
N1265	D14	FY95	Foundress	P192	RNA-seq	SAMN13938116	SRX7644440
N1265	D14	YPBY	Worker	P193	RNA-seq	SAMN13938115	SRX7644441
N1265	D14	Yellow	Egg layer	P196	RNA-seq	SAMN13938114	SRX7644442
N1265	D14	green spot	Foundress	P197	RNA-seq	SAMN13938113	SRX7644443
N1265	D14	Red	Worker	P198	RNA-seq	SAMN13938112	SRX7644444
N1283	D14	GYWW	Worker	P201	RNA-seq	SAMN13938111	SRX7644445
N1283	D14	PPPW	Worker	P202	RNA-seq	SAMN13938110	SRX7644446
N1283	D14	BYOY	Worker	P203	RNA-seq	SAMN13938109	SRX7644447
N1283	D14	PYY	Egg layer	P204	RNA-seq	SAMN13938108	SRX7644448
N1283	D14	G91	Worker	P205	RNA-seq	SAMN13938107	SRX7644449
N1283	D14	GWPY	Worker	P206	RNA-seq	SAMN13938106	SRX7644450
N1283	D14	OYOW	Worker	P208	RNA-seq	SAMN13938105	SRX7644451
N1283	D14	BWWY	Worker	P209	RNA-seq	SAMN13938104	SRX7644452
N1283	D14	YWBB (from 12-42)	Foundress	P210	RNA-seq	SAMN13938103	SRX7644453

N1283	D14	G68	Worker	P211	RNA-seq	SAMN13938102	SRX7644454
N1283	D14	WOGW	Worker	P212	RNA-seq	SAMN13938024	SRX7644455

Supplementary Table 3.
Details of the statistical analysis

Figure	Comparison	p-value	Adjusted p (BH)	Adjusted p (Holm)	Number of tests	Test type
1F	Control-D4	7.85*10^-5	2.36*10^-4	2.36*10^-4	2	t-test
1F	Control-D14	0.20	0.20	0.20	2	t-test
2B(top)	Control-Eggless	3.13*10^-6	1.25*10^-5	1.25*10^-5	4	wilcoxon
2B(top)	Control-D1	0.005	0.01	0.015	4	wilcoxon
2B(top)	Control-D4	0.074	0.0987	0.148	4	wilcoxon
2B(top)	Control-D14	0.72	0.72	0.72	4	wilcoxon
2B(mid)	Queen-workers (Control)	0.041	0.0683	0.164	5	wilcoxon
2B(mid)	Queen-workers (Eggless)	0.759	0.759	0.759	5	wilcoxon
2B(mid)	Queen-workers (D1)	0.018	0.0683	0.09	5	wilcoxon
2B(mid)	Queen-workers (D4)	0.041	0.0683	0.164	5	wilcoxon
2B(mid)	Queen-workers (D14)	0.081	0.101	0.164	5	wilcoxon
2B(bot)	Queen-workers (Control)	0.02	0.033	0.06	5	wilcoxon
2B(bot)	Queen-workers (Eggless)	0.465	0.465	0.465	5	wilcoxon
2B(bot)	Queen-workers (D1)	0.214	0.268	0.428	5	wilcoxon
2B(bot)	Queen-workers (D4)	1.16*10^-4	5.8*10^-4	5.8*10^-4	5	wilcoxon

2B(bot)	Queen-workers (D14)	7.58*10^-4	1.90*10^-4	3.03*10^-3	5	wilcoxon
2G	Control-Eggless	<2.2*10^-16	-	-		
4B		1.4*10^-10	-	-		pearson
4D	B.juncea - P.canadensis	4.7*10^-3	-	-		wilcoxon
4G	Control-D4	2.5*10^-8	-	-		wilcoxon
S1C(l)	Control-D4	5.24*10^-7	-	-		t-test
S1C(r)	Control-D4	1.75*10^-7	-	-		t-test
S2D	Control-Eggless	<2.2*10^-16	-	-		t-test
S2E	Vitellogenin (control)	2*10^-5	-	-		pearson
S2E	Apolipophorin 3 (control)	0.12	-	-		pearson
S2E	Vitellogenin (D4)	1.3*10^-4	-	-		pearson
S2E	Apolipophorin 3 (D4)	0.024	-	-		pearson
S4D	Control	<2.2*10^-16	-	-		pearson
S4D	D4	<2.2*10^-16	-	-		pearson
S4E		<2.2*10^-16	-	-		pearson
S5A	Control-D1	0.00716		0.0072		wilcoxon
S5B	DNMT1 (Control)	0.26	-	-		pearson
S5B	DNMT1 (D4)	0.25	-	-		pearson

S5B	DNMT2 (Control)	0.4	-	-		pearson
S5B	DNMT2 (D4)	0.16	-	-		pearson
S5B	TDG long (Control)	0.25	-	-		pearson
S5B	TDG long (D4)	0.78	-	-		pearson
S5B	TDG short (Control)	0.065	-	-		pearson
S5B	TDG Short (D4)	0.94	-	-		pearson
S5B	TET (Control)	0.057	-	-		pearson
S5B	TET (D4)	0.46	-	-		pearson
S5B	UHFR1 (Control)	0.15	-	-		pearson
S5B	UHFR1 (D4)	0.016	-	-		pearson
S5B	UNG (Control)	0.24	-	-		pearson
S5B	UNG (D4)	0.58	-	-		pearson
S5C	DNMT1 (Control)	0.39	-	-		pearson
S5C	DNMT1 (D4)	0.66	-	-		pearson
S5C	DNMT2 (Control)	0.78	-	-		pearson
S5C	DNMT2 (D4)	0.049	-	-		pearson
S5C	TDG long (Control)	0.36	-	-		pearson
S5C	TDG long (D4)	0.3	-	-		pearson

S5C	TDG short (Control)	0.93	-	-		pearson
S5C	TDG short (D4)	0.066	-	-		pearson
S5C	TET (Control)	0.0072	-	-		pearson
S5C	TET (D4)	0.066	-	-		pearson
S5C	UHFR1 (Control)	0.27	-	-		pearson
S5C	UHFR1 (D4)	0.84	-	-		pearson
S5C	UNG (Control)	0.24	-	-		pearson
S5C	UNG (Control)	0.84	-	-		pearson

Supplemental Theory

Adolfo Alsina and Steffen Rulands

In this Supplemental Theory we provide details of the derivation of the mathematical framework underlying the results presented in the main text.

Contents

1 Introduction **3**

2 Model definition **5**

2.1 Dynamics on the molecular level 5

2.2 Interactions between individuals 6

2.3 Coupling between population-level interactions and gene expression . . 8

3 Phase diagram **10**

4 Derivation of the mean-field master equation **12**

4.1 Continuum limit 15

5 Derivation of the phase portrait **16**

6 Stability of the steady state **22**

7 Prediction of experimental data **26**

7.1 Marginal distribution of ovary sizes 26

7.2 Experimental parameters 29

8 Numerical simulations **31**

1 Introduction

The primitive social insect *Polistes canadensis* forms societies composed of a single queen, which is the only reproductive individual, and multiple workers. After removal of the queen the remaining workers are capable of reprogramming and producing a new queen. In this study, we therefore use *Polistes canadensis* as a model system to understand how biological systems form stable structures in noisy environments which can, at the same time, be rapidly remodelled upon specific cues.

In mathematical terms, within the scope of this work, we define *specialisation* to denote situations where the distribution of phenotypes in the steady state of the population exhibits clearly separated modes which are stable on time scales much longer than the intrinsic time scales of the system. Such a scenario is typically the result of effective barriers resulting either explicitly from a predefined potential landscape or implicitly, as entropic barriers, from the kinetic rules governing the dynamics.

Given such a situation, one would naively expect that the reestablishment of the population structure after removal of one of the modes occurs on similar time scales as the lifetime of the metastable steady state [1]. With the term *plasticity* we denote the capability of the population to repopulate a missing mode on a time scale similar to the intrinsic time scales, i.e. much faster than the lifetime of the metastable steady state.

Our approach to understanding specialization and plasticity in the *Polistes* society combines multi-scale experimental measurements with biophysical modelling. Different nests of *Polistes* were monitored in video, their individuals dissected and their brains subjected to multi-modal sequencing (see details in Supplementary Material), both before and at different time points after queen removal. This experimental approach provides information on different levels of biological organisation: the social, individual and molecular scales. Integrating the information provided by these experiments on different scales allows us to understand the mechanistic principles underlying

the robust specialisation and rapid reprogramming in *Polistes* societies.

In this supplement, we provide details of the calculations underlying the derivation and analysis of a multi-scale biophysical model to understand the simultaneous capability for robust specialization and plasticity in the *Polistes* society. In our approach we seek to define the simplest model that capable of describing the experimental phenomenology. Specifically, we do not assume non-linearities unless motivated by experimental observations. Although our model therefore necessarily does not reflect the full complexity of wasp behaviour, such a reductionist approach will allow us to obtain mechanistic understanding underlying the principles governing specialisation and plasticity. Starting from a model that describes the non-Markovian dynamics of the joint probability distribution we derive the time evolution equations for the marginal probabilities of gene expression levels and ovary sizes. We then take the mean-field limit to derive the structure of the phase space.

The structure of this document is as follows: in section 2 using a master equation formalism we construct a model that describes molecular processes taking place in each individual which we then couple through interactions on the population scale. In section 7 we draw on this model to derive the time evolution of experimental observables, such as the distribution of ovary sizes and global nest activity. Then, in section 3, we construct the rich phase diagram as a function of the interaction rate and the interaction sensitivity. In section 4 we derive a mean-field master equation from the full stochastic model describing the time evolution of the individual and collective dynamics. These results are then used in section 5 to build a phase portrait of the system, which we use to understand how specialization and plasticity are regulated. In section 6 we consider the stability of the systems with respect to fluctuations and investigate the stabilising effect of epigenetic processes on the social structure. Finally, in section 8 we describe the numerical implementation of the model.

2 Model definition

2.1 Dynamics on the molecular level

Our RNA-seq analysis showed that genes, which are upregulated in queens compared to workers in control nests (queen genes), are collectively expressed in all workers during the reprogramming process. This suggests, on the one hand, that the expression of these queen genes can be described by a single degree of freedom. We here denote this degree of freedom on the molecular level by n_i, taken to be the total abundance of proteins corresponding to queen genes in individual i. On the other hand, our analysis demonstrates that these queen genes are constitutively expressed in the absence of queen interactions, a finding corroborated by previous studies on individual workers in Polistes and other social insects [2]. Therefore, in the absence of interactions, the dynamics on the molecular level are described by the production and degradation of proteins with rates μ and δ, respectively. The time evolution of the probability of finding protein abundances $\{n_k\}$ in a population of $N+1$ insects, $P(\{n_k\},t)$, is governed by a master equation of the form

$$\frac{d}{dt}P(\{n_k\},t) = \sum_{i=1}^{N+1} \mu \left[P(\{n_i-1\},t) - P(\{n_i\},t)\right] + \delta \left[(n_i+1)P(\{n_i+1\},t) - n_i P(\{n_i\},t)\right] , \tag{1}$$

where $P(\{n_i \pm 1\},t)$ represents the probability of observing protein levels $\{n_1,\ldots,n_i \pm 1,\ldots,n_{N+1}\}$ across the population. The first two terms describe the Poissonian production of proteins and the remaining terms their degradation. From here on, we will omit the time dependence of $P(\{n_k\},t)$ unless necessary. The ensuing stochastic dynamics give rise to a steady state characterised by a distribution of protein abundances with an average value of μ/δ. Therefore, in the absence of interactions, the dynamics converge to a steady state where all individuals express queen genes.

2.2 Interactions between individuals

To break the symmetry between individuals and select a single queen the dynamics on the molecular level need to be coupled to a collective process on the population scale. In the context of a nest, individuals interact in different ways, including fighting. These fighting interactions are directed, meaning that in every interaction there is a dominant individual, the attacker, and a subdominant one.

The rate with which individual i is subject to a subdominant interaction with individual j, K_{ij}, can be written as the total interaction rate between both, a_{ij}, times the conditional probability b_{ij} that individual i is subdominant in such an interaction, $K_{ij} = a_{ij}b_{ij}$. To calculate a_{ij} we note that according to our video recordings, which we correlated with ovary size measurements in individual insects, the interaction rate of an individual insect increases with ovary size and the expression level of queen genes, $a_i \equiv \sum_{j\neq i} a_{ij} \propto n_i$. The pairwise interaction rate therefore is proportional to the probability that both individuals interact in a given time interval, $a_i a_j$, times the probability that this interaction involves individuals i and j, $2/[N(N-1)]$, $a_{ij} = 2a_i a_j/[N(N-1)] \propto 2n_i n_j/[N(N-1)]$, such that we set $a_{ij} = \omega n_i n_j$ where ω is proportional to $2/[N(N-1)]$. To derive the conditional probability that individual i is subdominant, b_{ij}, we resort to previous work showing that the outcome of an interaction is strongly determined by the concentrations of Insect Juvenile Hormone (JH), a hormone involved in ovaries development [3]. Indeed, in our video recording of control nests we found that, in an interaction between two individuals the subdominant one is with high probability the one with the smaller ovaries. Given that ovary size is a proxy for queen gene expression, we take the conditional probability of an individual being subdominant to depend on the gene expression difference between the two interacting individuals, $b_{ij} = b(n_j - n_i)$.

Taken together, the conditional probability of i being the subdominant individual

in an interaction between i and j is

$$b(n_j - n_i) = \Theta(n_j - n_i)\,. \tag{2}$$

The total rate of subdominant interactions that individual i receives then takes the form

$$\omega \sum_{j \neq i} K(n_i, n_j)\,, \tag{3}$$

with the interaction kernel being defined by

$$K(n_i, n_j) = n_i n_j \Theta(n_j - n_i)\,. \tag{4}$$

In order to derive the above form of the interaction kernel we have assumed that individuals can accurately measure the gene expression levels of the insect they are interacting with. A more realistic approach is to take into account that sensing of gene expression levels is associated with an uncertainty. If this uncertainty is distributed following a normal distribution with zero mean and variance σ^2, the probability of individual i being subdominant in an interaction with individual j is

$$\int_{-\infty}^{\infty} \frac{1}{\sqrt{2\pi\sigma^2}} e^{\eta^2/2\sigma^2} \theta(n_j - n_i + \eta) d\eta = \frac{1}{2}\left[1 + \mathrm{Erf}\left(\frac{n_j - n_i}{\sqrt{2\sigma^2}}\right)\right]\,, \tag{5}$$

with the error function defined as $\mathrm{Erf}(x) = \int_0^x e^{-y^2}\,dy$. With this the interaction kernel reads

$$K(n_i, n_j) = \frac{n_i n_j}{2}\left[1 + \mathrm{Erf}\left(\frac{n_j - n_i}{\sqrt{2\sigma^2}}\right)\right]\,, \tag{6}$$

where σ^2 determines how precisely an individual can measure gene expression of other individuals. The above expression is difficult to manipulate both numerically and analytically due to the presence of the error function. It is, however, well approximated

by another sigmoidal function

$$K(n_i, n_j) = n_i n_j \frac{e^{-\lambda(n_i - n_j)}}{1 + e^{-\lambda(n_i - n_j)}} \,, \tag{7}$$

with $\lambda = 2\sigma^{-1}$ denoting the sensing sensitivity.

2.3 Coupling between population-level interactions and gene expression

How do interactions on the population scale get translated into changes on the molecular scale? An interaction leads to the transient increase in the concentration of factors influencing queen gene expression in the subdominant individual. While the precise nature of the pathways and molecular species involved in this process are not fully known in *Polistes*, to break the symmetry between insects and to obtain a single queen they must counteract the gene expression dynamics and thus can only be repressive. This reasoning is also supported by previous studies in *Polistes* showing that inhibition of fertility-linked compounds requires physical interactions [4]. In the following, we therefore refer to these factors as *queen gene repressors*. Based on this, we make a minimal set of assumptions to describe the coupling between interactions and gene expression dynamics:

1. Interactions lead to the transient presence of queen gene repressors in the subdominant individual and
2. these factors, while present, inhibit the expression of queen genes.

Not knowing the precise nature of the molecular pathways triggered by an interaction we model these repressive factors as a binary variably, $q_i \in \{0, 1\}$, representing the absence or presence of repressive factors in individual i, respectively. In our model, queen gene repressors inhibit the expression of queen genes which mathematically translates to a vanishing rate of the production of queen gene products. Therefore, the production rate of queen gene products reads $\mu(1 - q_i)$. The dynamics of the repressive

factors themselves are comprised of two processes: queen gene repressors are activated in the subdominant individual upon an interaction ($q_i = 1$) and then persist for a time drawn from a distribution $\Gamma(t)$. If the activity of queen gene repressors involves sufficiently many steps Γ will be approximately normally-distributed with a variance much smaller than the mean. We therefore set $\Gamma(t) \propto \delta(t_{\text{per}} - t)$, where t_{per} is the typical persistence time of queen gene repressors.

With this in mind we can now mathematically define the time evolution of the joint probability $P(\{n_k, q_k\})$. To this end, let us consider a population of $N+1$ individuals, each with two degrees of freedom: the number of queen gene products, n_k, and the state of presence of repressive factors, q_k. Taken together, the stochastic dynamics is described by in terms of the non-Markovian master equation of the form

$$\begin{aligned}\frac{d}{dt}P(\{n_k, q_k\}) = \sum_{i=1}^{N+1} \Bigg\{ & \mu(1-q_i)\left[P(\{n_i-1, q_i\}) - P(\{n_i, q_i\})\right] \\ & + \delta\left[(n_i+1)P(\{n_i+1, q_i\}) - n_i P(\{n_i, q_i\})\right] \\ & + \Gamma(t_i^{\text{int}})P(\{n_i, 1\})(1-2q_i) + \omega \sum_{j\neq i} K_{ij} P(\{n_i, 0\})(2q_i - 1) \Bigg\},\end{aligned} \tag{8}$$

where t_i^{int} is a realisation of a stochastic process defined by the time elapsed since the last subdominant interaction of individual i evaluated at time t. Equation (8) accounts for the full stochastic dynamics of the population, encompassing gene expression dynamics and the dynamics of the repressive factors. Rescaling the number of gene products and time appropriately, we obtain the master equation in dimension-

less form,

$$\frac{d}{d\tau}P(\{r_k,q_k\}) = \sum_{i=1}^{N+1}\Big\{(1-q_i)\left[P(\{r_i-\epsilon,q_i\})-P(\{r_i,q_i\})\right]$$
$$+\left[(r_i+\epsilon)P(\{r_i+\epsilon,q_i\})-r_iP(\{r_i,q_i\})\right]$$
$$+\Gamma(\tau_i^{\text{int}})P(\{r_i,1\})(1-2q_i)$$
$$+\alpha\frac{\mu}{\delta}\sum_{j\neq i}K(r_i,r_j)P(\{r_i,0\})(2q_i-1)\Big\}, \qquad (9)$$

where $\alpha = \omega/\delta$ is the interaction rate in units of the degradation time, dimensionless time $\tau = \mu \cdot t$, rescaled queen gene expression levels $r_k = \delta n_k/\mu$ and $\epsilon = \delta/\mu$.

3 Phase diagram

In order to gain insight into the range of possible behaviours of the system we performed stochastic simulations of the dynamics defined by Equation (9) as described in section 8. We scanned the phase space of the system as a function of two parameters that control the coupling between the molecular and population scales: the interaction rate, α, and the sensitivity, λ. For a given combination of these parameters we sampled 50 independent trajectories of the stochastic nest dynamics defined in Equation (9). We set the population size to $N = 10$ individuals and $\tau_{\text{per}} = 1$. We took averages over the the asymptotic ovary size (for a definition see section 7) at a time long after the steady state had been reached, $\tau = 60$. The results are shown in Figure 2e of the main text.

In this phase diagram, we identify three regimes based on the value of the asymptotic number of queens, defined here as those individuals whose gene expression value is larger or equal than 0.8, which corresponds to 80% of the steady state value. In the limit $\alpha \ll 1$ interactions occur on a much slower time scale than the molecular

processes, effectively uncoupling the molecular and the population scales and leading to a steady state where all individuals obtain a queen phenotype. On the other hand, for $\alpha \gg 1$, the asymptotic composition of the society depends on the sensitivity, λ: If λ is small, $\lambda \ll 1$, subdominant interactions affect all individuals with roughly equal probability. As a result, we observe that the asymptotic dynamics converges to a state represented solely by individuals with low expression of queen genes (workers).

On the other hand, if the scale defined by the sensitivity λ is at least of equal order than the typical variability of queen gene expression values, i.e. individuals are able to distinguish phenotypically relevant changes in gene expression, $\lambda > 1$, interactions break the symmetry between individuals and a bimodal social steady state arises. While multiple queens can exist for precisely defined values of α, exactly one single queen is guaranteed to emerge as long as the interaction rate exceeds a threshold value and individuals are capable of distinguishing "macroscopic" gene expression states. Therefore, by integrating antagonistic dynamics on different spatial scales, *Polistes* societies establish a single queen robustly for a large range of parameters, avoiding the need for fine-tuning of parameters that fluctuate in time and from nest to nest.

To locate the empirical parameter values in the phase diagram we estimated the parameters from the experimental data presented in Figure 2b of the main text, obtaining $\alpha_{\exp} \approx 1$. To estimate λ we counted the number of subdominant queen interactions. Out of 17 interactions involving queens in the control and late-commitment phases we observed 0 interactions where the queen was subdominant. The maximum likelihood estimate for the error rate using the beta-distribution as a (conjugate) prior therefore is 1/17. Using the definition of the interaction kernel we find an analytical expression for the error rate,

$$2\int_{-\infty}^{0} d\Delta r (1+\exp(-\lambda\Delta r))^{-1} = 2\ln 2/\lambda$$

Solving for λ we find that $\lambda \approx 24$. According to the phase diagram, these parameters indeed lead to the emergence of a single queen in support of the proposed paradigm.

4 Derivation of the mean-field master equation

The model defined in Equation (9) predicts many of the features of the reprogramming process and properties of the steady state. But its high dimensionality and non-Markovianity render it unsuitable for analytical treatment. In order to understand how specialization and plasticity are simultaneously achieved in the *Polistes* society we start from Equation (9) and develop a continuum, mean-field description. We will then employ this continuum description in section 5 to explore the structure of the phase space as a function of the individual and collective degrees of freedom.

To begin, we consider the time evolution of a single "tracer" individual embedded in a nest with a given composition $P\left(\{r_i, q_i\}_{i=1}^N\right)$ and study the evolution of the probability of finding the tracer in the state (r, q) given the nest composition, $P(r, q) \equiv P(r, q|\{r_i, q_i\})$. In this approach we consider the nest as a "bath", which is not affected by the tracer and which determines the fluctuations of the individual tracer dynamics. The master equation for the time evolution of the queen gene expression level and repressor state of the tracer individual reads

$$\begin{aligned}\frac{d}{d\tau}P(r,q) &= (1-q)\left[P(r-\epsilon,q)-P(r,q)\right] \\ &+ \left[(r+\epsilon)P(r+\epsilon,q)-rP(r,q)\right] \\ &+ \Gamma(\tau^i_{int})P(r,1)(1-2q)+\alpha\frac{\mu}{\delta}\sum_{j=1}^{N}K(r,r_j)P(r,0)(2q-1)\,. \end{aligned} \tag{10}$$

To obtain a time evolution equation for the marginal probability $P(r)$ we first need to integrate out the queen gene repressors variable, q. To this end, we formally define a trajectory dependent time (cf. Figure 1) as

$$\tilde{\tau}(t) = \int_0^{\tau} d\tau' \prod_{i\in\mathcal{I}} f(\tau_i - \tau')\,, \tag{11}$$

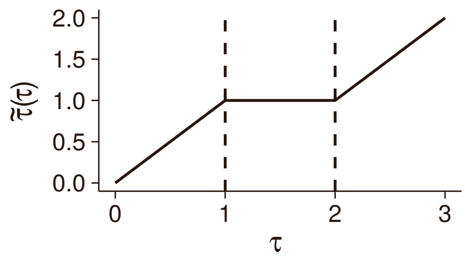

Figure 1: Example depicting the dependence of the trajectory dependent time $\tilde{\tau}$ as a function of the physical time. $\tilde{\tau}$ grows as the same rate as the physical time τ unless an interaction takes place (in this case at $\tau = 1$), in which case it remains at the same value for a time τ_{per}.

where $\{\tau_i\}_{i\in\mathcal{I}}$ is the set of times when an interaction, and hence a $q = 0$ to $q = 1$ transition, takes place, and the function $f(\tau_i - \tau)$ is defined as

$$f(\tau_i - \tau) = \begin{cases} 0 & \tau \in [\tau_i, \tau_i + \tau_{per}] \\ 1 & \text{otherwise}\,. \end{cases} \tag{12}$$

From this definition it follows that $\tilde{\tau}$ increases in the same manner as τ if and only if $q = 0$ and is constant otherwise. The evolution of the system in the new time coordinate $\tilde{\tau}$ thus coincides with the evolution with respect to τ when $q = 0$ and its effect is collapsed to a single time point when $q = 1$. This is equivalent to considering dynamics where $q = 0$ for all times and where a suitably chosen number, M, of queen gene products are instantaneously degraded at times $\tau \in \{\tau_i\}_{i\in\mathcal{I}}$. M is the typical number of molecules degraded in a time interval of length τ_{per}, $M \approx r[1 - \exp(-\tau_{\text{per}})]$. The master equation describing such dynamics takes the form

$$\begin{aligned} \frac{d}{d\tilde{\tau}}P(r) &= [P(r-\epsilon) - P(r)] \\ &+ [(\epsilon + r)P(r+\epsilon) - rP(r)] \\ &+ \alpha\frac{\mu}{\delta}\sum_{j=1}^{N}\left[K(r+M, r_j)P(r+M) - K(r, r_j)P(r)\right]\,. \end{aligned} \tag{13}$$

Next, we consider time scales much longer than typical interaction times. To derive a continuum description, we rescale the persistence time of the repressive factors $\tau_{\rm per}$ in such a way that the average number of degraded queen gene products after an interaction $M(\tau_{\rm per})$ is, on average, equal to a queen gene product unit, ϵ. We simultaneously keep the total effect of repressive interactions in a long time interval $[\tau_1, \tau_2]$ on the concentration of queen gene products, $\int_{\tau_1}^{\tau_2} d\bar{\tau}' M(\bar{\tau}_{\rm per}) \prod_{i\in\mathcal{I}} \delta(\bar{\tau}_i - \bar{\tau}')$, invariant. To this end, the above constraint dictates a corresponding rescaling of the interaction rate, α. Intuitively, such a coarse-graining operation corresponds to a homogeneous distribution of interaction events in the time domain for sufficiently long time scales. As the total effect of interactions on long time scales remains unchanged the structure of the phase portrait remains unchanged as well. With this, we obtain

$$\begin{aligned}\frac{d}{d\tilde{\tau}}P(r) = {} & [P(r-\epsilon) - P(r)] \\ & + [(r+\epsilon)P(r+\epsilon) - rP(r)] \\ & + \alpha' \sum_{j=1}^{N} [K(r+\epsilon, r_j)P(r+\epsilon) - K(r, r_j)P(r)]\end{aligned} \tag{14}$$

where $\alpha' \approx \alpha\mu M/\delta$ is the rescaled interaction rate. The mean-field master equation is then obtained by setting, $x = r/\Omega$, and performing a Kramers-Moyal expansion to the lowest order [5]. For simplicity retaining the symbol r to represent queen gene expression, we find

$$\partial_{\tilde{\tau}} r = \tilde{\alpha}_1(x) = 1 - r - \tilde{\alpha} \sum_{j=1}^{N} K(r, r_j)\,, \tag{15}$$

where

$$\tilde{\alpha}_1(r) = \Omega^{-1} \int_{-\infty}^{\infty} dr'(r' - r) W(r'|r) \tag{16}$$

is the first jump moment and $\tilde{\alpha} = \alpha'/\Omega^2$. We henceforth refer to the dual version of Equation (15) describing the evolution of the probability density as the mean field

master equation,

$$\partial_{\tilde{\tau}} P(r) + \partial_r \left[(1-r)P(r)\right] = \tilde{\alpha}\partial_r \left(P(r) \sum_{j=1}^{N} K(r, r_j) \right) . \tag{17}$$

4.1 Continuum limit

We next we take the continuum limit of Equation (15) on the number of individuals, $N \to \infty$, to obtain the time evolution of queen gene expression levels in the "tracer" wasp,

$$\partial_t r = 1 - r - \tilde{\alpha} \sum_{i \neq j} K(r, r_j) = 1 - r - \tilde{\alpha} \int_0^{\infty} \sum_{j=1}^{N} K(r, r')\delta(r' - r_j) \tag{18}$$

$$1 - r - \tilde{\alpha} \int_0^{\infty} \sum_{j=1}^{N} K(r, r')\delta(r' - r_j) \xrightarrow[N\to\infty]{} 1 - r - \tilde{\alpha} \int_0^{\infty} K(r, r') f(r') dr' \tag{19}$$

where the empirical distribution function $f(r') \equiv \sum_j \delta(r_j - r')$ represents the fraction of individuals in the nest having queen gene expression between r and $r + dr$, as well as the time evolution of the population composition,

$$\partial_{\tilde{\tau}} f(r) + \partial_r \left[(1-r)f(r)\right] = \tilde{\alpha}\partial_r \left(f(r) \int_0^{\infty} K(r, r') f(r') dr' \right) . \tag{20}$$

This represents the mean-field description of Equation (9) which is valid in the limit of large populations and time scales, i.e. in the steady state. As we will discuss below, while Equation (19) and Equation (20) are not suitable for quantitatively describing the reprogramming dynamics they nevertheless are capable of providing mathematical insight into the mechanisms underlying the regulation of specialisation and plasticity. It is interesting to note that Equation (20) is conceptually similar to equations described in other biological contexts, such as quorum-sensing bacteria [6] or Mitogen competition by stem cells [7]. In this context, it is also worth to note that in

spatially structured systems specialisation can be achieved by spatially separating different phenotypes, such as via the Turing mechanism, spinodal decomposition, lateral inhibition or via external signalling gradients [8, 9]. If the spatially homogeneous state is unstable such systems are naturally "plastic".

The dynamics on the molecular scale are coupled through a collision-like functional that represents the effect of repressive interactions. In the next section we will study how such a coupling gives rise to specialization and plasticity as emergent properties of the system.

5 Derivation of the phase portrait

The individual and collective dynamics derived in the previous section provide a mean-field description of the system. In Equation (20) the time evolution of $f(r, \bar{\tau})$ is governed by a term describing the molecular dynamics (second term on the left hand side) and a term describing the collective behaviour on the population scale (term on the right hand side). The equation gives rise to a steady state when the molecular dynamics is balanced by the population-level feedback. To understand the relaxation dynamics to the steady state, and its stability, it is instructive to consider the co-evolution of the molecular scale, given by the queen gene expression level r, and the population scale, represented by the distribution $f(r, t)$. In this section we will illustrate the results of our analysis by means of a phase portrait of the multi-scale dynamics. Although the limits we take, such as taking the mean-field limit, do not accurately reflect the full biological complexity, our approximations are validated by comparison to simulations of the full stochastic dynamics.

The starting point of our analysis are Equation (19) and Equation (20), describing the individual and collective dynamics, respectively. Taken together, these equations describe the co-evolution of the queen gene expression level of an individual and the population structure. Stable fixed points of such dynamics represent possible pheno-

types in the society, such as queen and workers. We calculate these fixed points from the intersection of the nullclines of the system. These nullclines are given by

$$0 = 1 - r - \tilde{\alpha} \int_{-\infty}^{\infty} K(r, r') f(r') dr' \tag{21}$$

$$\partial_r \left[(1 - r) f(r, t) \right] = \tilde{\alpha} \partial_r \left(f(r, t) \int_{-\infty}^{\infty} K(r, r') f(r', t) dr' \right) . \tag{22}$$

Equation (21) and Equation (22) provide the basis for understanding the steady state of the system as a function of the population composition and the fixed points of the individual dynamics. Although the population composition is represented by a probability distribution, f, its functional nature complicates intuitive interpretations of the relaxation dynamics. In order to obtain a more intuitive picture, we reduce the system to a two-dimensional system describing the coupled evolution of r and the first moment of the population composition, $\langle r \rangle$. The starting point of this approximation is Equation (19),

$$\partial_{\tilde{\tau}} r = 1 - r - \tilde{\alpha} \int_{0}^{\infty} r r' \Theta(r' - r) f(r') dr' \,, \tag{23}$$

describing the evolution of the molecular degree of freedom of a tracer individual. The integral in the right hand side of the equation represents the effect of the interactions received by the tracer individual. In the limit of long times compared with the typical interaction time scale, $t \gg \alpha^{-1}$, the effect of interactions can be approximated by the overall effect of interacting with an effective individual with gene expression level $\langle r \rangle$,

$$\partial_{\tilde{\tau}} r \approx 1 - r - \tilde{\alpha} r \langle r \rangle \Theta(\langle r \rangle - r) \,, \tag{24}$$

where $\langle r \rangle = \int_0^\infty r f(r, t) dr$. Further, by multiplying Equation (20) by r and integrating over r, we obtain the time evolution of the first moment,

$$\partial_{\tilde{\tau}} \langle r \rangle = 1 - \langle r \rangle - \tilde{\alpha} \int_{0}^{\infty} dr \int_{0}^{\infty} dr' K(r, r') f(r) f(r') \,, \tag{25}$$

where $\int_0^\infty dr \int_0^\infty dr' K(r,r')f(r)f(r')$ is the total interaction rate at time t in the nest. To close the system of equations we approximate $\int_0^\infty dr \int_0^\infty dr' K(r,r')f(r)f(r') \approx \langle r\rangle^2/2$ where the factor 2 ensures arises due to double counting of subdominant interactions. Finally, our reduced system of equations reads

$$\partial_{\tilde{T}} r \approx 1 - r - \tilde{\alpha} r \langle r\rangle \Theta(\langle r\rangle - r)\,, \tag{26}$$

$$\partial_{\tilde{T}} \langle r\rangle \approx 1 - \langle r\rangle - \tilde{\alpha}\frac{\langle r\rangle^2}{2}\,. \tag{27}$$

Equation (26) and Equation (27) form a two-dimensional system of equations that is amenable for a bidimensional graphical representation given by a phase portrait. The solutions of these equations in the steady state provide the fixed points of the dynamics. In the steady state we find,

$$\langle r\rangle_0 = \frac{\sqrt{2\tilde{\alpha}+1}-1}{\tilde{\alpha}}\,. \tag{28}$$

For $\tilde{\alpha} = 0$ Equation (26) and Equation (27) admit only one stable solution corresponding to high queen gene expression levels, $r_0 = 1$ (Figure 2). For $\alpha > 0$, we find three solutions if

$$\langle r\rangle > \frac{\sqrt{4\tilde{\alpha}+1}-1}{2\tilde{\alpha}}\,, \tag{29}$$

which in the steady state is always fulfilled for $\tilde{\alpha} > 0$. These three solution comprise two stable branches, at $r_0 = 1$ and $r_2 = 1/(1+\tilde{\alpha}\langle r\rangle)$ and an unstable branch at $r = \langle r\rangle$. Formally, the system therefore comprises a saddle node bifurcation with the population structure as a bifurcation parameter. Intrinsic perturbations, which do not change the value of $\langle r\rangle$, are therefore suppressed by the bistable dynamics in the steady state. Substituting the population steady state,$\langle r\rangle_0$ we obtain as intersections

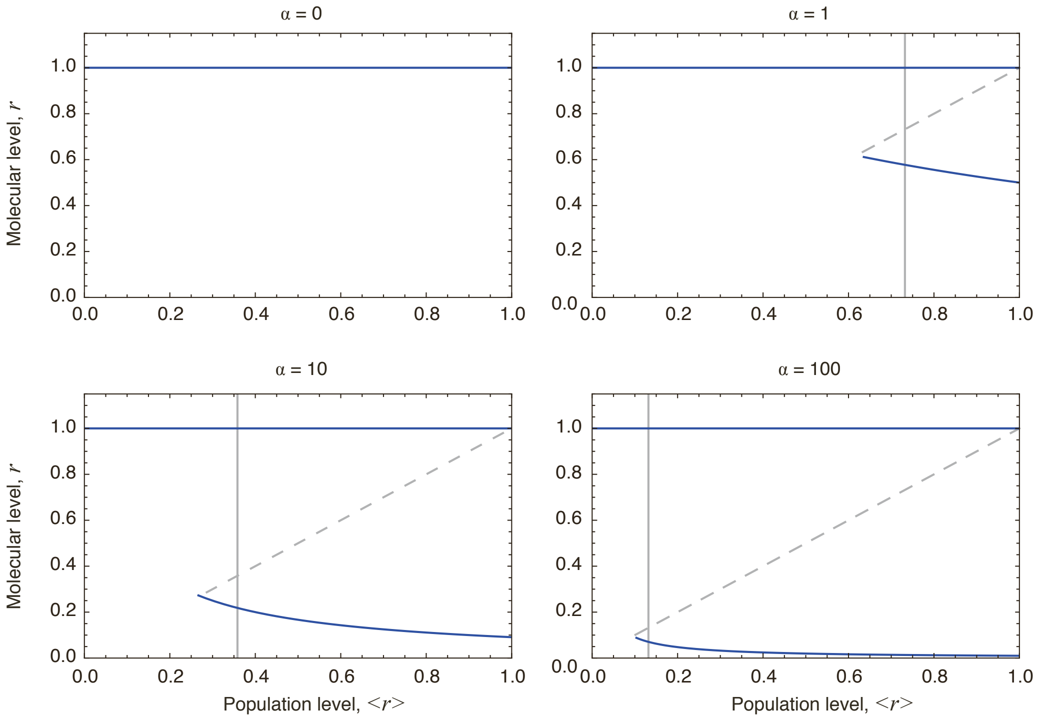

Figure 2: Bifurcation diagram showing fixed points of Equation (26) as a function of $\langle r \rangle$. Stable branches are represented by solid lines and unstable branches by dashed lines. Vertical lines denote the nullclines of Equation (27). For $\tilde{\alpha} > 0$ the dynamics undergo a saddle node bifurcation with the population composition, $\langle r \rangle$, as a bifurcation parameter.

of the nullclines for $\tilde{\alpha} > 0$,

$$
\begin{aligned}
r'_0 &= 1\,, \\
r'_1 &= \frac{\sqrt{2\tilde{\alpha}+1}-1}{\tilde{\alpha}}\,, \\
r'_2 &= \frac{1}{\sqrt{2\tilde{\alpha}+1}}\,.
\end{aligned}
\tag{30}
$$

The flow of the system towards the steady state is represented in Figure 3 and in Figure 3a of the main text. In this representation the x axis corresponds to the molecular degree of freedom and the y axis to the population composition, represented by the first moment of the distribution, $\langle r \rangle$. A population in this portrait is represented

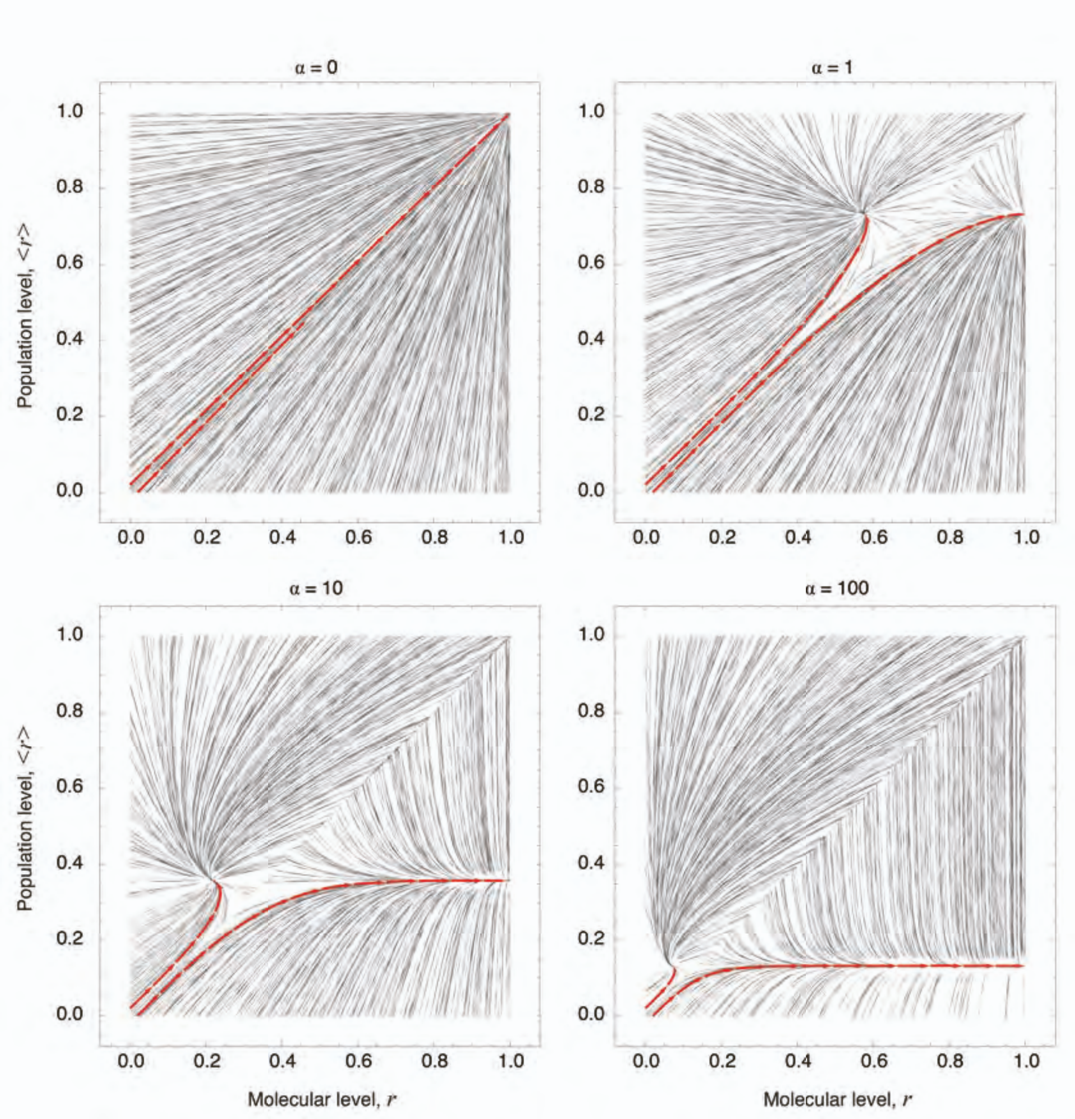

Figure 3: Phase portraits of Equation (26) and Equation (27) for different values of $\tilde{\alpha}$. Random trajectories are represented by gray lines and two trajectories originating around the point $(0, 0)$ corresponding to a nest with only individuals lacking queen gene expression are highlighted in red.

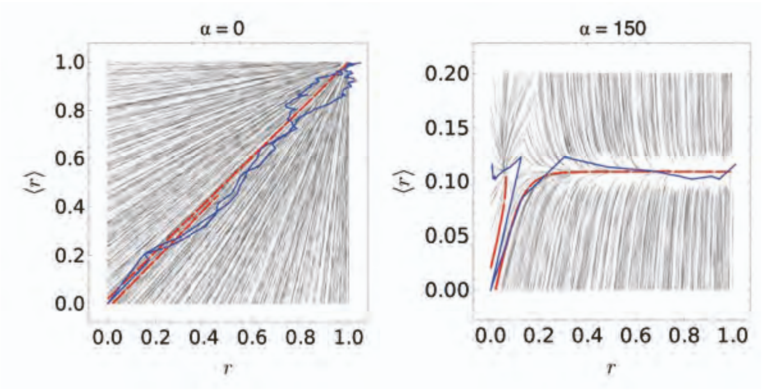

Figure 4: Phase portraits of Equation (26) and Equation (27) for different values of $\tilde{\alpha}$. As in Figure 3 random trajectories are represented by gray lines and two trajectories originating around the point $(0, 0)$ corresponding to a nest with only individuals lacking queen gene expression are highlighted in red. Blue lines denote trajectories from stochastic simulation of the full stochastic system, Equation (9).

by a set of N points, each of them with a different value of the x coordinate, but all of them with the same value of the y coordinate. Changes in the molecular degree of freedom are reflected in changes in the population composition that again affect all individuals identically.

The flow in the mean-field limit qualitatively represents the flow obtained from stochastic simulations of Equation (9) as shown in Figure 4. Therefore, while our calculations are strictly only valid in the limit of an infinite population size and on long time scales, Equation (26) and Equation (27) accurately represent the qualitative structure of the deterministic phase space of Equation (9).

After the removal of the queen the population obtains a homogeneous structure narrowly distributed around the separatrix defined by $r = \langle r \rangle$ and $r = \langle r \rangle \ll 1$. From these initial conditions, the dynamics evolve rapidly along the separatrix. On this separatrix fluctuations can drive each individual either to the queen attractor or to the worker attractor.

6 Stability of the steady state

In section 5 we have analyzed the fixed points of the dynamics of the system and studied their stability against uncorrelated and correlated perturbations. To this end, we employed a mean-field approximation effectively neglecting fluctuations arising due to the finite number of individuals and stochasticity in interaction times, Equation (19) and Equation (20). We will ask how the stability of the nest and the social order, manifest in a clear separation of queen and worker phenotypes, can be maintained in the presence of strong fluctuations. Indeed, typical population sizes range between 8 to 30 individuals and interactions occur on similar time scales as typical protein degradation times, suggesting that fluctuations stabilise the steady state of the society.

To quantitatively understand how fluctuations affect the stability of the society we begin by estimating the persistence time of the society given typical time scales of molecular and population level processes. To this end, we first calculate the probability that any individual upregulates its queen genes in the time interval between two consecutive interactions, and thereby destabilised the society, by producing a sufficient number of queen gene products. If interaction events are statistically independent the waiting time T between consecutive interactions follows an exponential distribution, $P(T) = \omega e^{-\omega T}$. Denoting the time scale of molecular processes (e.g. the time needed for an individual to upregulate the queen genes) by T_δ, the probability that T is longer than T_δ is

$$P(T > T_\delta) = \int_{T_\delta}^{\infty} P(T)dT = e^{-\omega T_\delta}\,. \tag{31}$$

Thus, the probability of a single worker being subject to a repressive interactions in a time interval of length T_δ is $1 - e^{-\omega T_\delta}$. In a nest with N workers, the probability of all of them being subject to a repressive interaction in a time interval T then is $\left(1 - e^{-\omega T_\delta}\right)^N$. Conversely, the probability that at least one worker is capable of

upregulating its queen genes between two consecutive interactions is

$$p = 1 - \left(1 - e^{-R}\right)^N \ , \tag{32}$$

where $R = \omega T_\delta$ is a dimensionless parameter that represents the ratio between the molecular and the interaction time scales. The number of interactions before an insect is capable of upregulating its queen genes follows a geometric distribution with mean $1/p$. Therefore, the persistence time of the society, τ. is

$$\tau = \omega^{-1}\left(p^{-1} + R\right) \ . \tag{33}$$

The theoretical prediction given by Equation (33) indicates that in order to obtain a queen turnover time consistent with the experimentally measured value of roughly 27.5 weeks (Supplementary Table 1) the ratio between molecular and interaction time scales, R, must be larger or equal than 9 (Supplementary Fig. 4c). Given the experimental estimation of the time interval between two consecutive interactions, this would imply that gene expression states are stable for at least three days in the absence of interactions. However, transcriptional queen signatures are already established in *Polistes* three days after queen removal [10].

This result, based on a simple comparison of time scales and the constitutive expression of queen genes in the absence of interactions, is in seeming contradiction to the experimentally observed stability of *Polistes* societies over much longer time scales. This raises the question of how the social structure is stabilised. In the remainder of this section we will ask whether the observed reduction of gene expression variance in workers (Fig. 4b of the main text) provides a mechanism for stabilising the society in the long term.

We begin by considering the probability distribution of queen gene expression values, $P(r)$, in workers. In a population composed of N workers, the probability of observing

a new queen is equal to the probability that at least one worker up regulates the queen genes. Assuming that the queen gene expression value workers is distributed according to $P(r)$, the probability of observing a new queen is equal to the probability of finding an insect with queen gene expression larger or equal to one,

$$p = \int_1^\infty P(r) dr \,, \tag{34}$$

Consistent with our previous calculation, Equation (33), the persistence time of the society then is

$$\tau(\sigma) = \omega^{-1} \left[\frac{1}{1-(1-p)^N} + R \right] \,. \tag{35}$$

The variance of $P(r)$, σ^2 is determined by an interplay between queen gene expression and queen interactions. Our main conclusions from this analysis are independent of the details of these processes. We here provide two examples to illustrate our results. If subdominant interactions with the queen are statistically independent and happen at a rate independent of queen gene expression levels in workers, then queen gene expression levels in workers follow an exponential distribution, $P(r) = \exp(-\omega r/\mu)$, with μ and ω being the rate of queen gene expression and interactions, respectively. With the variance given by $\sigma^2 = (\mu/\omega)^2$ we obtain $p = \exp(-1/\sigma)$ and a persistence time of the society of

$$\tau = \omega^{-1} \left[\frac{1}{1-(1-e^{-1/\sigma})^N} + R \right] \,. \tag{36}$$

In a more realistic setting corresponding to our model the rate of repressive interactions of a worker is proportional to its level of queen gene expression. In this case, in analogy to stochastic growth processes with resetting, queen gene expression levels

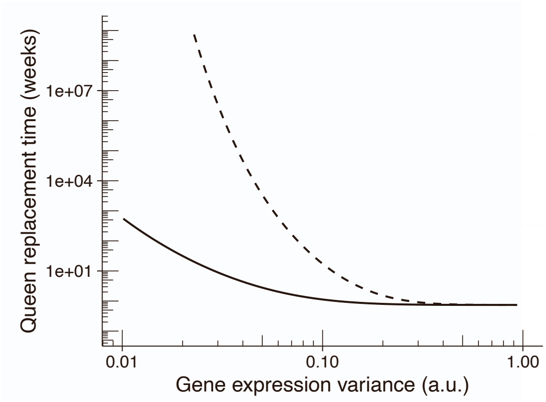

Figure 5: Lifetime of the society as a function of the population variance for normally (dashed line) and exponentially (solid line) distributed populations for values of the parameters $\omega = 3.7$ interactions/day, $R = 5.55$ and $N = 20$.

of workers follow a truncated normal distribution [11],

$$P(r) = \sqrt{\frac{2\omega^2}{\pi\mu^2}} e^{-\frac{r^2\omega^2}{2\mu^2}} . \quad (37)$$

With a variance $\sigma^2 = \mu^2/\omega^2$ we find in this case that

$$\tau = \omega^{-1} \left(\frac{1}{1 - (1 - \text{Erfc}\left[(2\sigma^2)^{-1/2}\right])^N} + R \right) , \quad (38)$$

where $\text{Erfc}(x) = 1 - \text{Erf}(x)$ is the complementary error function.

Taken together, variations in the variance of queen gene expression levels across workers have a strong, orders of magnitude effect on the stability of the society as a whole Figure 5. This holds qualitatively true independently of the details of the molecular of population level processes. These results, together with our finding that gene body methylation is associated with a decrease in gene expression variance in *Polistes* (Fig. 4b of the main text) shows that DNA methylation plays a significant role in stabilising the society in the long term.

7 Prediction of experimental data

Having derived the model describing the evolution of the joint probability of gene expression and queen gene repressors in Equation (9), we now set out to fix its parameters and predict the experimental data. To derive a mathematical description that is less dependent on parameters describing poorly understood molecular processes, such as the dynamics of queen gene repressors, we now derive an effective description of the time evolution of the marginal distribution of ovary sizes. To this end, we will derive a description based on ovary growth starting from the model derived above. As ovary sizes are a mere downstream effect of the queen gene expression dynamics (see below), such an effective description should be structurally similar to the master equation describing the time evolution of the marginal distribution of queen gene expression levels, Equation (13), if the time scales are chosen appropriately.

7.1 Marginal distribution of ovary sizes

In order to predict the time evolution of the probability of observing ovaries of size $\{o_k\}$ at time t, $P(\{o_k\}, t)$, we first note that many of the queen genes have functions related to reproduction or are directly responsible for ovary development, such that the instantaneous rate of ovary growth is given by a function that depends on the expression level of queen genes, $g(n_k)$. The function $g(n)$ summarises a cascade of molecular pathways which are not understood in detail. We here make the simplest possible assumption about the functional form of $g(n)$, namely that it depends linearly on gene expression levels and that ovaries grow if the gene expression level exceeds a threshold, n_0, and shrink otherwise. Therefore, the rate of ovary growth takes the form $g(n) \propto n - n_0$. As eggs are laid once they have reached a mature size, we impose a reflective boundary condition on o_i at a size o_0 which we set to be the maximum ovary size observed in the experiment, $o_0 = 2.5\,\mathrm{mm}$. With this, switching back to dimensional quantities, the time evolution of the conditional probability of ovary sizes

follows a master equation of the form

$$\frac{d}{dt}P(\{o_k\},t|\{n_k,q_k\}) = g(n_k)\theta(n_k-n_0)\left[P(\{o_k-1\},t|\{n_k,q_k\}) - P(\{o_k\},t|\{n_k,q_k\})\right] \tag{39}$$
$$- g(n_k)\theta(n_0-n_k)\left[P(\{o_k+1\},t|\{n_k,q_k\}) - P(\{o_k\},t|\{n_k,q_k\})\right] .$$

As, in this model, the expression level of queen genes is independent of ovary size, the joint probability $P(\{n_k,o_k\},t)$ factorizes as $P(\{n_k,o_k\},t) = P(\{o_k\}|\{n_k\},t)P(\{n_k\},t)$. Therefore, the dynamics of the joint probability of $\{n_k,o_k,q_k\}$ are described by

$$\begin{aligned}\frac{d}{dt}P(\{n_k,o_k,q_k\}) = \sum_{i=1}^{N+1}\Big\{&\mu(1-q_i)\left[P(\{n_i-1,o_i,q_i\}) - P(\{n_i,o_i,q_i\})\right] \\ &+\delta\left[(n_i+1)P(\{n_i+1,o_i,q_i\}) - n_iP(\{n_i,o_i,q_i\})\right] \\ &+g(n_i)\theta(n_i-n_0)\left[P(\{n_i,o_i-1,q_i\}) - P(\{n_i,o_i,q_i\})\right] \\ &-g(n_i)\theta(n_0-n_i)\left[P(\{n_i,o_i+1,q_i\}) - P(\{n_i,o_i,q_i\})\right] \\ &+\Gamma(t_i^{\text{int}})P(\{n_i,o_i,1\})(1-2q_i) \\ &+\omega\sum_{j\neq i}K(n_i,n_j)P(\{n_i,o_i,0\})(2q_i-1)\Big\} ,\end{aligned} \tag{40}$$

with $g(n)$ denoting the rate of ovary growth for a gene expression level n.

With the aim of comparing our simulation results to the experimental ovary dissection data we first integrate out gene expression, yielding an equation describing the evolution of ovary sizes. If the persistence time of the repressive effect of interactions plus the typical production time of gene products is smaller than the typical time between two interactions, individuals alternate periods of growing and shrinking of their ovaries with the duration of these periods determined by the ratio between the typical interaction and persistence times plus the queen gene production times. Taking this limit, the ovary growth rate only depends on whether queen gene expression is above

or below the threshold n_0. For that purpose we define a random variable,

$$s_i = \Theta(n_i - n_0)\,, \tag{41}$$

such that the ovary growth rate is proportional to $2s_i - 1$.

The time evolution of the random variable s_i is linked to the dynamics of n_i. This gives rise to an explicit time delay in the ovary equation. Specifically, following an interaction, an individual with $s_i = 1$ needs a time t_{off} to degrade enough gene products and activate the pathways responsible for flipping the ovary growth state, $s_i = 0$. On the other hand, if an individual with $s_i = 0$ does not engage in a subdominant interaction during a time $t_{\text{on}} \approx t_{\text{off}} + t_{\text{per}}$, given by the sum of the persistence time of queen gene repressors and the time needed to express queen genes beyond a level n_0 and activate pathways related to reproduction, it will again flip the ovary growth state, $s_i = 1$.

In the long time limit the ovary size of a given individual is determined by the sign of the diference $T_{s_i=1} - T_{s_i=0}$ where $T_{s_i=j} = \int_0^t dt' \theta(s_i - j)$ is the time that the ovary growth rate spends in the state j. In this limit, the instantaneous value of gene expression r at time t is a good indicator of the sign of $T_{s_i=1} - T_{s_i=0}$, and hence of ovary growth. Taking this into account the interaction kernel can be expressed in terms of ovary size and we obtain the effective master equation governing the evolution of the

marginal distribution of ovary sizes,

$$\frac{d}{dt}P(\{o_k, s_k\}) = \sum_{i=1}^{N+1}\Big\{ g(s_i)\left[P(\{o_i - 1, s_i\}) - P(\{o_i, s_i\})\right] \\ + \delta(t_i^{\text{int}} - t_{\text{off}})P(\{o_i, 1\})(1 - 2s_i) + \delta(t_i^{\text{int}} - t_{\text{on}})P(\{o_i, 0\})(2s_i - 1)\Big\}, \tag{42}$$

where t_i^{int} is, as before, the time elapsed since the last subdominant interaction of individual i. Equation (42) provides a description of the system at the ovary level that retains the foremost characteristics present in the experimental data, i.e. the presence of multiple queens in the nest shortly after reprogramming and the posterior relaxation towards an analogous state to the control, as demonstrated by the results in Figure 2F of the main text. The existence of explicit time delays t_{on} and t_{off} is responsible for the transient observation of multiple queens during reprogramming. Specifically, such an overshoot arises if the value of t_{off} is of similar magnitude as the time scale associated with ovary growth.

7.2 Experimental parameters

The model defined in Equation (42) includes several parameters. In this section we provide justifications for the parameter values used to predict the experimental observables in Supplementary Fig. 2f-h of the main text. In the derivation of the model, Equation (42), we did not assume any non-linearities unless supported by experimental data. Such non-linearities, for example in the relation between ovary growth and gene expression, naturally exist in any biological system. Therefore, we have to exercise some caution in interpreting these parameters in literal biological terms. We still expect, however, that the order of magnitude of parameters is not altered by unknown

Parameter	Value	Justification
ω (Interaction rate)	3.7 day^{-1}	Measurements from video recordings presented in Supplementary Fig. 4A of the main text.
τ_{off} (Typical time between an interaction and changes in ovary growth)	2.5 days	We estimated this parameter based on the observation of 2-3 egg layers in the early commitment phase.
o_r (Ovary growth rate)	$0.25\,\text{mm}\,\text{day}^{-1}$	The first egg layer is observed 6 days after queen removal with a size of mature eggs of 1.5 mm.

Table 1: Summary of parameter values used for predicting experimental data.

non-linearities and can be estimated by independent observations from experiments or the literature. Parameter values are summarised in Table 1.

The time evolution of the global activity in a nest (Supplementary Fig. 2h) is determined by a component which is independent of the fighting interactions involved in the regulation of the reprogramming process and a component reflecting these interactions. Only the latter component is reflected in the model. We note that this component is proportional to the total interaction rate. From Equation (42) we find that this interaction rate is

$$\sum_{ij} o_i o_j f(o_i) f(o_j) = \left(\sum_i o_i f(o_i)\right)\left(\sum_j o_j f(o_j)\right) = \langle o_i \rangle^2 .$$

In Supplementary Fig. 2h, to take into account the different contributions to the global activity mentioned above, an we added an offset value to the theoretical prediction such that the empirical and theoretical values matched in the control phase. Then, activity levels were rescaled such that the maximum of both curves matched.

8 Numerical simulations

We performed kinetic Monte Carlo simulations following Gillespie's algorithm to obtain approximate solutions to master equation for a population of N individuals [12]. Out of the different possible processes - production of a new gene product, degradation of gene products, interactions, degradation of the queen gene repressors or ovary development - one was randomly selected with probability proportional to the overall rate of the respective process (in vector form)

$$\left(\mu, \delta n_i, \alpha K(n_i, n_j), \delta(t^i_{int} - t_{per}), g(n_i)\right) \ . \tag{43}$$

Once a process has been selected the state of the system is updated depending on the selected reaction and finally, the simulation time is advanced an amount Δt drawn from an exponential distribution of parameter λ given by the inverse sum of the overall rates,

$$\lambda = \left\{ \sum_i \left(\mu + \delta n_i + \sum_j K(n_i, n_j) + \delta(t^i_{int} - t_{per}) + g(n_i) \right) \right\}^{-1} \ . \tag{44}$$

Unless specified otherwise the following parameters were used for all the simulations $\mu = 500$, $\delta = 1$, $n_0 = 250$, $\lambda = 10$, $t_{per} = 1$. The ovary growth rate was chosen so that ovaries were mature in 6 days, as observed experimentally.

All simulations were implemented in Julia and the source code is available upon request to the authors.

References

1. Assaf, M. & Meerson, B. WKB Theory of Large Deviations in Stochastic Populations. *J. Phys. A: Math. Theor.* **50,** 263001 (2017).

2. Hamilton, A., Shpigler, H., Bloch, G., Wheeler, D. & Robinson, G. in *Hormones, Brain and Behavior* 421–451 (Elsevier, 2017).
3. Roeseler, P.-F., Roeseler, I., Strambi, A. & Augier, R. Influence of Insect Hormones on the Establishment of Dominance Hierarchies among Foundresses of the Paper Wasp, Polistes Gallicus. *Behav Ecol Sociobiol* **15,** 133–142 (1984).
4. Oi, C. A. *et al.* Do Primitively Eusocial Wasps Use Queen Pheromones to Regulate Reproduction? A Case Study of the Paper Wasp Polistes Satan. *Front. Ecol. Evol.* **7,** 199 (2019).
5. Gardiner, C. W. *Handbook of Stochastic Methods for Physics, Chemistry, and the Natural Sciences* 3rd ed (Springer-Verlag, 2004).
6. Bauer, M., Knebel, J., Lechner, M., Pickl, P. & Frey, E. Ecological Feedback in Quorum-Sensing Microbial Populations Can Induce Heterogeneous Production of Autoinducers. *eLife* **6,** 1–38 (2017).
7. Jörg, D. J., Kitadate, Y., Yoshida, S. & Simons, B. D. Competition for Stem Cell Fate Determinants as a Mechanism for Tissue Homeostasis. arXiv: `1901.03903 [physics, q-bio]` (2019).
8. Cross, M. & Greenside, H. *Pattern formation and dynamics in nonequilibrium systems* (Cambridge University Press, 2009).
9. Rulands, S., Klünder, B. & Frey, E. Stability of Localized Wave Fronts in Bistable Systems. *Phys. Rev. Lett.* **110,** 038102 (2013).
10. Taylor, B. A., Cini, A., Wyatt, C. D. R., Reuter, M. & Sumner, S. The Molecular Basis of Socially Mediated Phenotypic Plasticity in a Eusocial Paper Wasp. *Nature Communications* **12,** 775 (2021).
11. Biró, T. & Néda, Z. Unidirectional Random Growth with Resetting. *Physica A: Statistical Mechanics and its Applications* **499,** 335–361 (2018).

12. Gillespie, D. T. Exact Stochastic Simulation of Coupled Chemical Reactions. *J. Phys. Chem.* **81,** 2340–2361 (1977).
