# Supplementary material for: Self-organization of plasticity and specialization in a primitively social insect
Source: Cell Syst. 2022 Sep 21;13(9):768–779.e4. doi: 10.1016/j.cels.2022.08.002 (PMC9512265; doi:10.1016/j.cels.2022.08.002)
Supplement: Document S3. Article plus supplemental information [file mmc9.pdf]

Cell Systems

Report

Self-organization of plasticity and specialization in a primitively social insect

Graphical abstract

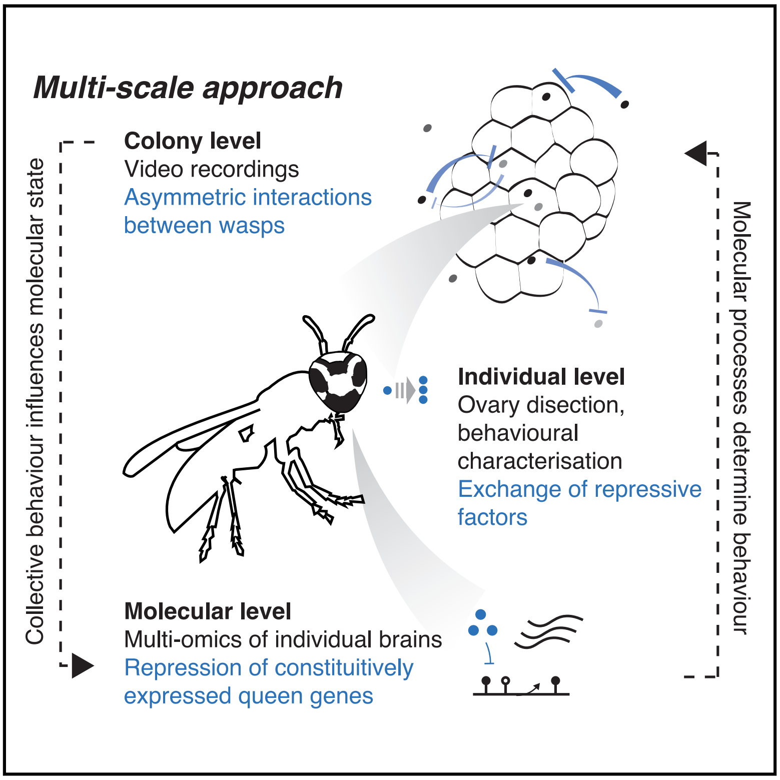

Highlights

- Experiments and theory reveal how *Polistes* wasps achieve specialization and plasticity
- Molecular upregulation of queen phenotype is balanced by colony-scale repression
- This allows *Polistes* to distinguish between intrinsic and extrinsic perturbations

Authors

Solenn Patalano, Adolfo Alsina, Carlos Gregorio-Rodríguez, ..., Seirian Sumner, Wolf Reik, Steffen Rulands

Correspondence

patalano@fleming.gr (S.P.),
wreik@altoslabs.com (W.R.),
rulands@pks.mpg.de (S.R.)

In brief

Combining theory with experiments, Patalano et al. show that both robust specialization and rapid plasticity in primitive social wasps result from a self-organized balance between the development of a molecular queen phenotype and its colony-scale inhibition. This allows *Polistes* to be stable against intrinsic molecular perturbations while reacting plastically to extrinsic cues.

Patalano et al., 2022, Cell Systems *13*, 768–779
September 21, 2022 © 2022 The Authors. Published by Elsevier Inc.
https://doi.org/10.1016/j.cels.2022.08.002

CellPress

CellPress
OPEN ACCESS

Cell Systems

Report

Self-organization of plasticity and specialization in a primitively social insect

Solenn Patalano,[1,14,16,*] Adolfo Alsina,[2,14] Carlos Gregorio-Rodríguez,[3] Martin Bachman,[4,5,6] Stephanie Dreier,[7] Irene Hernando-Herraez,[1] Paulin Nana,[8] Shankar Balasubramanian,[5,6,9] Seirian Sumner,[10] Wolf Reik,[1,11,12,15,17,*] and Steffen Rulands[2,13,15,18,*]

[1]Epigenetics Programme, Babraham Institute, Cambridge CB22 3AT, UK
[2]Max Planck Institute for the Physics of Complex Systems, Noethnitzer Str. 38, 01187 Dresden, Germany
[3]Departamento de Sistemas Informáticos y Computación, Universidad Complutense de Madrid, Plaza de Ciencias, 3, 28040 Madrid, Spain
[4]Discovery Science and Technology, Medicines Discovery Catapult, Alderley Park, Cheshire SK10 4GT, UK
[5]Cancer Research UK Cambridge Institute, University of Cambridge, Li Ka Shing Centre, Cambridge CB2 ORE, UK
[6]Yusuf Hamied Department of Chemistry, University of Cambridge, Cambridge CB2 1EW, UK
[7]Institute of Zoology, Zoological Society of London, Regent's Park, London NW1 4RY, UK
[8]Faculty of Agronomy and Agricultural Sciences, School of Wood, Water and Natural Resources, University of Dschang, Ebolowa Campus, P.O. Box 786, Ebolowa, Cameroon
[9]School of Clinical Medicine, University of Cambridge, CB2 0SP, Cambridge, UK
[10]Centre for Biodiversity and Environment Research, Department of Genetics Evolution and Environment, Division of Biosciences, University College London, Gower Street, London WC1E 6BT, UK
[11]Wellcome Trust Sanger Institute, Hinxton CB10 1SA, UK
[12]Centre for Trophoblast Research, University of Cambridge, Cambridge CB2 3EG, UK
[13]Center for Systems Biology Dresden, Pfotenhauer Str. 108, 01307 Dresden, Germany
[14]These authors contributed equally
[15]Senior author
[16]Present address: B.S.R.C "Alexander Fleming," 34 Fleming Street, 16672 Vari, Greece
[17]Present address: Altos Labs Cambridge Institute, Cambridge CB21 6GP, UK
[18]Lead contact
*Correspondence: patalano@fleming.gr (S.P.), wreik@altoslabs.com (W.R.), rulands@pks.mpg.de (S.R.)
https://doi.org/10.1016/j.cels.2022.08.002

SUMMARY

Biological systems have the capacity to not only build and robustly maintain complex structures but also to rapidly break up and rebuild such structures. Here, using primitive societies of *Polistes* wasps, we show that both robust specialization and rapid plasticity are emergent properties of multi-scale dynamics. We combine theory with experiments that, after perturbing the social structure by removing the queen, correlate time-resolved multi-omics with video recordings. We show that the queen-worker dimorphism relies on the balance between the development of a molecular queen phenotype in all insects and colony-scale inhibition of this phenotype via asymmetric interactions. This allows *Polistes* to be stable against intrinsic perturbations of molecular states while reacting plastically to extrinsic cues affecting the whole society. Long-term stability of the social structure is reinforced by dynamic DNA methylation. Our study provides a general principle of how both specialization and plasticity can be achieved in biological systems. A record of this paper's transparent peer review process is included in the supplemental information.

INTRODUCTION

Biological systems have the remarkable capacity to build and maintain complex spatio-temporal structures. Such structures are often surprisingly robust in noisy environments, and their formation relies on the integration of regulatory processes on vastly different spatial scales of organization, from the molecular level to tissue or population-level feedback (Cross and Greenside, 2009). Although historically theoretical and experimental research has focused on the processes underlying the formation of complex structures (Ocko et al., 2019; Peleg et al., 2018) such as the self-organization of cells into complex organs (Davies, 2013), in recent years, it has become clear that biological systems also have the remarkable capacity to break up and rebuild these structures (Merrell and Stanger, 2016; Kennedy et al., 2017). As an example, colonies of social insects rely on the long-term specialization of individuals into distinct castes, such as queen and worker polyphenisms (West-Eberhard, 2003). Although such phenotypes can be stable over years in the face of environmental noise, individuals are nevertheless capable of being rapidly phenotypically reprogrammed: upon receipt of specific cues, they undergo a transient phase in which an

 © 2022 The Authors. Published by Elsevier Inc.
This is an open access article under the CC BY license (http://creativecommons.org/licenses/by/4.0/).

individual's behavior normally associated with a specific caste is rapidly altered in order to perform a different task from the one it performed initially (plasticity) (Herb et al., 2012; Simola et al., 2016; Sheng et al., 2020; Todd et al., 2019).

The regulation of specialization typically relies on the establishment of distinct stable states (bistability) which is often mediated by positive feedback loops (Strogatz, 2015). In bistable systems, transitions between stable phenotypic states require the crossing of a potential or entropic barrier, such that these systems are stable against noise over long periods of time. The same barrier that stabilizes the system, however, mitigates against the rapid re-establishment of a lost phenotype. As in this case, both processes are driven by the same stochastic forces, the time for reprogramming is then comparable with the time of spontaneous phenotype switching (Hänggi et al., 1990) such that stable specialization and rapid plasticity are not simultaneously achievable.

Here, we ask how biological systems can achieve rapid plasticity under specific environmental changes and simultaneously retain stable phenotypic specialization over time. As this problem cannot be resolved using bistability alone, we hypothesized that feedback across different scales of biological organization might give rise to additional degrees of freedom that allow for plasticity while maintaining stable specialization in the steady state.

To test this hypothesis, we used a well-established model system of phenotypic plasticity, colonies of primitively social paper wasps *Polistes canadensis* (Figure 1A) (Ferreira et al., 2013; Patalano et al., 2012, 2015; Sumner et al., 2006; Turillazzi and West-Eberhard, 1996; West-Eberhard, 1969). After the emergence of the foundress' (queen's) daughters (workers), a stable colony of paper wasps is established, with a single reproductive queen and 8–30 non-reproductive workers (specialization). If the queen dies (or is experimentally removed), the remaining workers can rapidly reprogram to generate a unique new queen, hence displaying strong phenotypic plasticity (Miyano, 1986; Strassmann et al., 2004; West-Eberhard, 1969). In order to understand the multi-scale processes regulating specialization and plasticity, we aimed to develop a unique experimental approach that allows us to describe the processes governing the regulation of specialization and plasticity upon *Polistes* queen removal at the molecular and colony scales and the interplay between both. A unique feature of this approach is that it allows measurements to be correlated at the level of the individual across different spatial scales. Based on these experiments, we sought to define a biophysical model that describes these processes and a theory that allows us to understand specialization and plasticity as an emergent property thereof.

We derive the main insight of this work from the synthesis of our experimental and theoretical approaches: we show that *Polistes* integrates processes on different layers of biological organization to distinguish between intrinsic perturbations of molecular states while reacting plastically to extrinsic cues affecting the society as a whole and thereby to simultaneously achieve rapid plasticity and robust specialization. Specifically, the society undergoes a saddle-node bifurcation governed by the population structure itself, thereby simultaneously achieving bistability in the steady state and transient, rapid convergence to the queen phenotype after colony-level perturbations.

RESULTS

Upregulation of queen genes in all workers after queen removal

To empirically study the interplay between processes on different scales of biological organization, we took the following experimental approach. After removal of the queen in stable and established colonies, we followed the relaxation dynamics until the re-establishment of a steady state in each colony simultaneously on vastly different spatial scales (Figure 1B): on the colony level by video recordings (individual interactions), on the individual level by characterizing their reproductive states (ovary dissection), and on the molecular level by whole genome multi-omics of the brains of individual insects (RNA sequencing [RNA-seq] of the transcriptome and bisulfite sequencing of the methylome). Although these experiments have individually been conducted in related systems in the past (West-Eberhard, 1969; Theraulaz et al., 1995; Taylor et al., 2021), we, here for the first time, correlate them on the level of individual insects. To characterize the reproductive and molecular dynamics occurring during the reprogramming experiments, we collected a subset of nests at five specific time points after queen removal (Figure 1C). Of the 26 nests monitored in their natural environment, queens were removed from 18 nests 2–3 weeks after the first emergence of workers (Table S1). After queen removal, a rapid reprogramming phase begins (eggless phase), at the end of which (6.55 ± SD 1.97 days) at least one reproductive individual is established (Figure S1A) as evidenced by the observation of egg laying behavior. The subsequent phases were determined on the basis of field observations: 1 day after the identification of at least one reproductive individual (D1 phase), 4–5 days after the establishment of a main reproductive individual (D4 phase), and 14 days after complete stabilization of the colonies (D14 phase).

We initially focused our analysis on the gene expression level, regarded as an important caste regulatory process in *Polistes* (Berens et al., 2015b, 2015a; Patalano et al., 2015; Sumner et al., 2006; Taylor et al., 2021). To identify changes in gene expression associated with queen replacement, we compared brain transcriptomes of all workers from 2 unmanipulated control nests (27 individuals) with transcriptomes of individuals from 3 other nests in which a main reproductive individual had been recently established (24 individuals, D4 phase). 227 genes were differentially expressed between control and D4 nests, with strong enrichment of genes previously associated with reproductive (queen-) phenotypes ($p = 3.1e-15$), henceforth referred to as "queen genes" (Patalano et al., 2015; Sumner et al., 2006) (Figure 1D). Consistent with this finding, we also found a strong enrichment of genes that correlate with ovary growth in control nests ($p = 1.5e-70$) as well as functional enrichments linked to metabolism, catalysis, and transport of lipids (Figure S1B). For instance, *Vitellogenin* and *Apolipophorin-3*, two genes involved in lipid transport, were strongly upregulated across individuals collected at D4, a conserved pathway activated for reproductive caste division in response to pheromone changes (Figure S1C) (Corona et al., 2016; Hunt et al., 2010; Holman et al., 2019). A principal component analysis confirmed that the workers collected during the D4 phase are all characterized by a shift in their transcriptome profile toward the transcriptome profile of established queens collected during the control phase

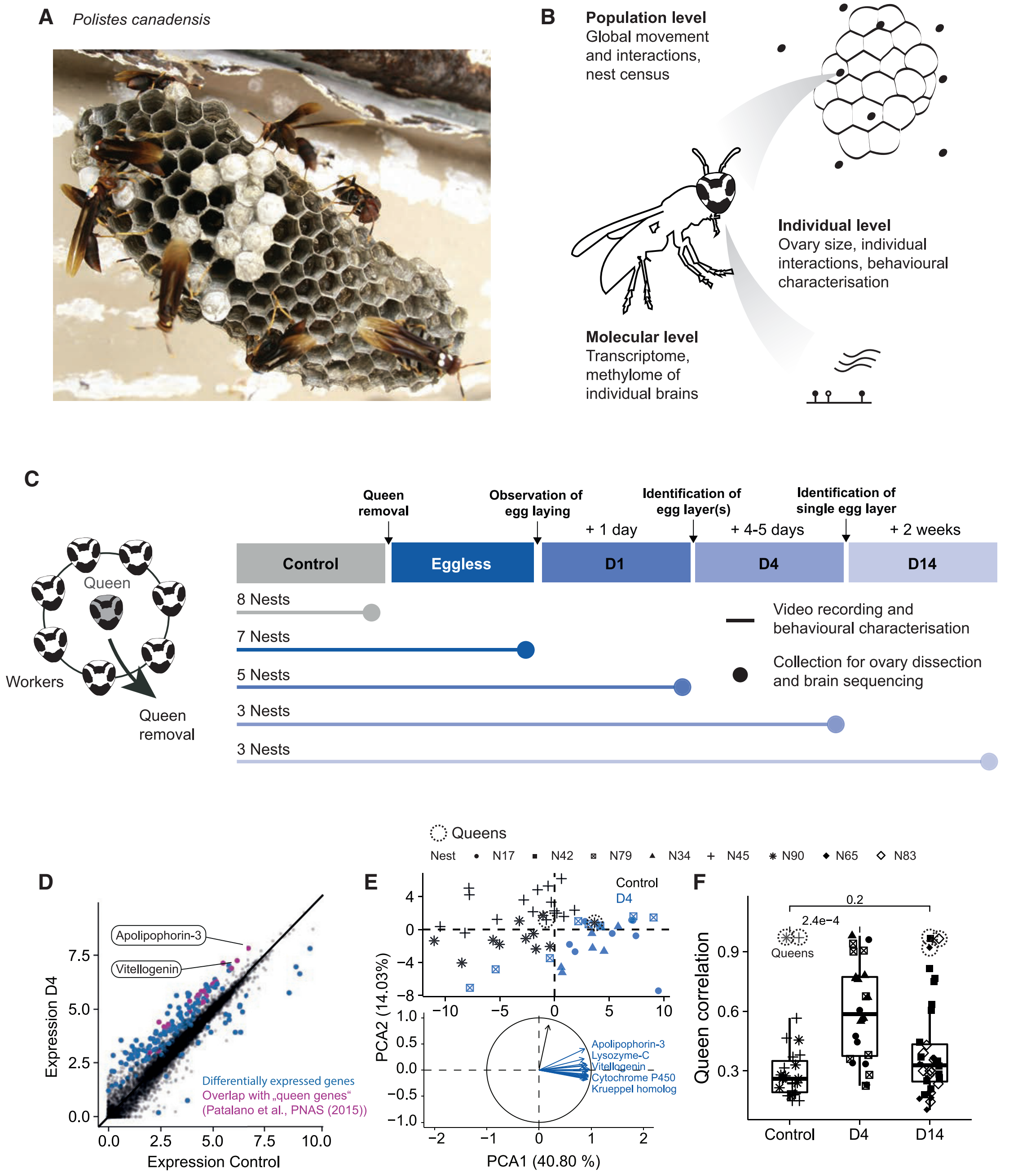

Figure 1. Upregulation of queen genes during reprogramming

(A) Photo of a typical nest of *Polistes canadensis*.

(B) Schematic of the multi-scale experimental approach.

(C) Schematic of the experimental time course of queen removal experiments. Control phase: 2–3 weeks prior to queen removal; eggless phase: time between queen removal and egg laying; D1 phase: day or day + 1 after identification of an egg layer(s); D4 phase: day + 4 or +5 after identification of the main egg layer; and D14 phase: 2 weeks after stable egg laying by the new queen.

(D) Gene expression levels in reads per kilobase of transcript per million mapped reads (RPKM), averaged over all individuals collected in control or D4 phases. Differentially expressed genes (DEGs) between phases are marked in blue. DEGs overlapping with queen genes identified in Patalano et al., 2015 are marked in pink.

(E) Principal component analysis of individual insects using DEGs. The radar plot shows the PCs of the 20 most contributing genes (blue: PC1; black: PC2).

(F) Global gene expression correlation of individuals compared with control (established) queens. Center line corresponds to the median, and lower and upper hinges to the 25th and 75th percentiles, respectively. p values were calculated using t tests between control and the two experimental phases and corrected for multiple testing using the Benjamini-Hochberg (BH) method. Control, n = 27; D1, n = 24; D4, n = 36.

CellPress
OPEN ACCESS

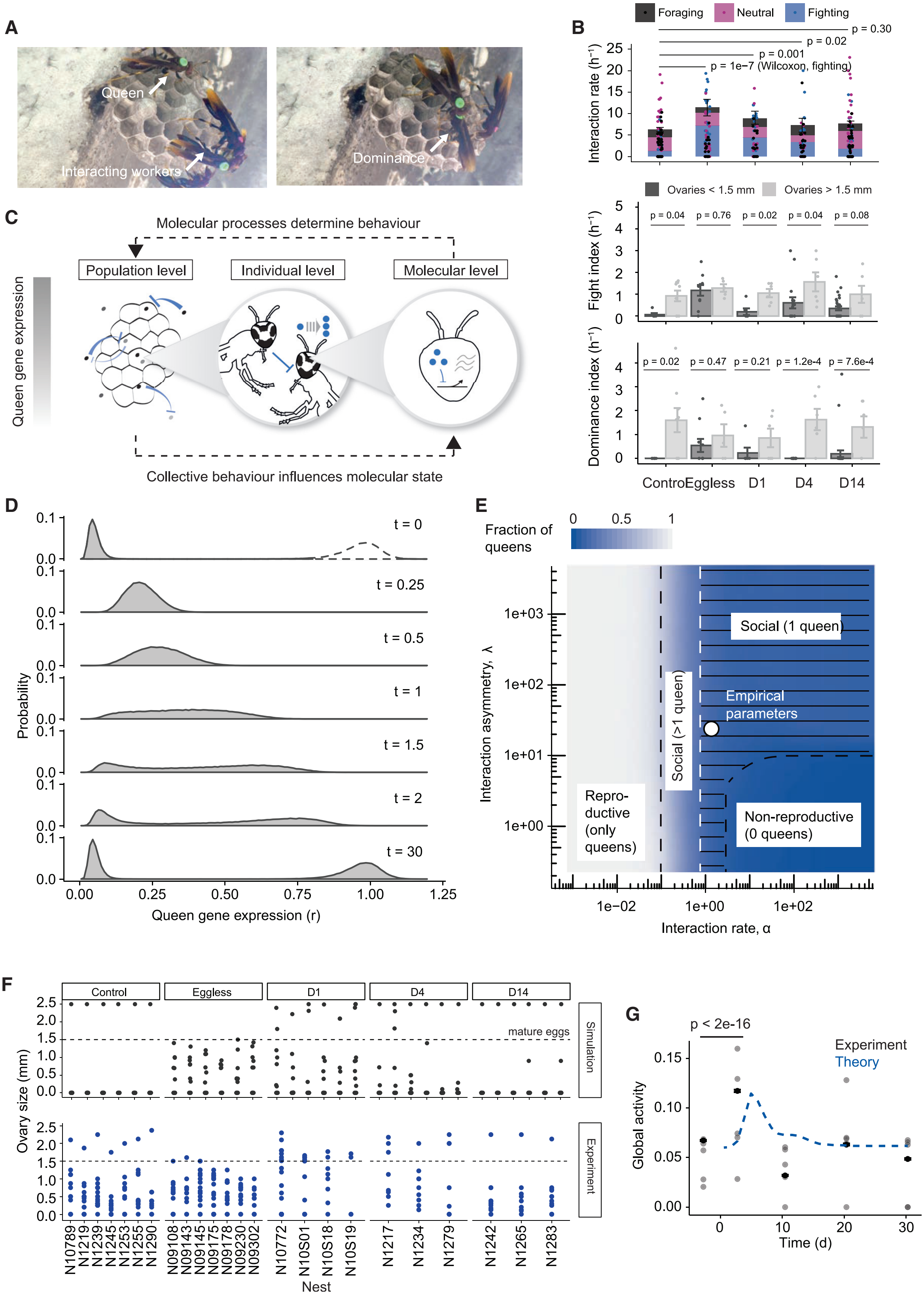

(legend on next page)

(Figure 1E). This observation is further supported by an increase in the correlation of queen gene expression profiles of workers in the D4 phase with queens in control nests (Figure 1F), consistent with the coordinated change of queen gene expression observed after queen removal in another *Polistes* species (Taylor et al., 2021). Taken together, these observations show that at the transcriptome level, queen removal rapidly causes a phenotypic switch to queens in all workers. However, this loss of worker phenotypes after queen removal is transient as individuals collected from 3 nests having already recovered their steady state (34 individuals, D14 phase) show gene expression profiles similar to unmanipulated workers (Figure 1F).

Understanding how the interplay between different scales of biological organization can give rise to specialization and plasticity requires understanding how perturbations on all scales are propagated across the different layers of biological organization. As this is experimentally unfeasible, our strategy is to derive a theory that predicts the multi-scale response of the society to perturbations. Such a theory would constitute a complete biophysical understanding of the multi-scale nest dynamics. As a first step, following the approach of statistical physics, we sought to define the simplest model compatible with the experimental data. In particular, in order for this model to be unsensitive to the partially unknown details of the complex processes underlying the regulation of the society, we did not assume any non-linear terms unless motivated by experimental observations. To begin deriving a biophysical description of the nest dynamics, we describe the molecular state of insect i by a single degree of freedom, r_i. This is supported by the observation that different kinds of molecular regulation respond to queen removal on similar time scales (hours), by our observation that the expression levels of different queen genes are correlated and by earlier observations in which *Vitellogenin* and *Apolipophorin* were upregulated within a few hours under hormonal influences resulting from the queen's absence or through induction (Edinger et al., 1997; Hamilton et al., 2016; Röseler, 1977; Röseler and Röseler, 1978). Importantly, our results do not depend on specific assumptions on the dynamics of particular molecular processes. Although r_i therefore represents a coarse-grained description of complex molecular states, we will, for specificity, henceforth refer to r_i as the concentration of queen gene products as an important instance of such states. The time evolution of the probability that a randomly chosen insect i has a concentration of queen gene products r_i, $P(r_i,\ t)$, is given by changes (so called probability fluxes) due to molecular processes, J_{mol}, and feedback by the colony level, J_{col}, $\partial_t P(r_i, t) = J_{mol} + J_{col}$. Our RNA-seq experiment shows that in the absence of the queen, individuals constitutively express queen genes (cf. Figures 1D–1F) (Alberts et al., 2015; Hamilton et al., 2016). The flux from the molecular level alone, J_{mol}, therefore reads in its simplest mathematical form $J_{mol} = \mathbb{E}^{-1}P(r_i,t) + \mathbb{E}^{1} r_i P(r_i,t)$, where the two terms can be interpreted as the production and degradation of queen gene products, respectively, and we have adopted the notation of step operators (van Kampen, 2007), $\mathbb{E}_k^n f(\{x_1, \ldots, x_N\}) = f(\{x_1, \ldots, x_k + n, \ldots, x_N\}) - f(\{x_1, \ldots, x_N\})$. Time is measured in units of the degradation time of queen gene products, and concentrations are scaled by the steady-state level of expression. Therefore, in the hypothetical case, where the society is regulated on the molecular level alone, the time evolution of $\partial_t P(r_i, t) = J_{mol}$ would lead to a steady state, termed attractor, where all individuals express queen genes at a high level given by a balance between production and degradation ($r_i = 1$). The existence of such a steady state in the molecular dynamics is sufficient for the derivation of all results presented below. As the evolution of dynamical systems is characterized by their attractors, taking into account other molecular processes (hormones, pheromones, gene expression,...) would not alter the conclusions drawn from this model (Strogatz, 2015).

Increase of dominant behavioral interactions during reprogramming

However, this symmetric convergence of all worker transcriptomes toward queen signatures (Figure 1E) cannot explain the eventual emergence of a single new reproductive individual. Thus, the restoration of a stable state necessarily requires the breaking of this symmetry by a collective process on the colony level.

To study whether there is a colony-level component in the regulation of reprogramming and phenotypic specialization, we analyzed over 17 h of video recordings from 5 nests that had undergone queen removal up to phase D4 (2 nests, 4 phases, 47 ± SD 22 min) and D14 (3 nests, 5 phases, 46 ± SD 21 min). We used computer vision analyses to quantify global activity (overall movement at the nest level, Figures 2A and 2B; Video S1) and manual video inspection to classify and quantify 6 stereotypic individual interactions (Figure 2A). We found a significant increase in both nest activity and the rate of interactions (Figures 2B, S2C, and S2D). Particularly, the rate of aggressive behaviors across individuals during the reprogramming process, especially during the eggless phase, and until the D4 phase increased

Figure 2. Plasticity and specialization emerge from antagonistic interactions on the molecular and the colony scales

(A) Snapshots of videos showing the interaction between wasps.

(B) Quantification of the interaction rate separated by type (top), the fight index (fraction of fighting interactions among all interactions per individual, middle), and the dominance index (fraction of dominant interactions among fighting interactions per individual, bottom). Bars depict mean ± SEM and dots represent individuals. p values were calculated from a Wilcoxon signed rank test between the corresponding phases and corrected for multiple testing using the BH method (n = 5 nests, 80 individuals).

(C) Schematic of the model (more details in supplemental theory).

(D) Probability densities of queen gene expression levels in a nest after queen removal obtained by stochastic simulations.

(E) Phase diagram showing different types of social structures as a function of the interaction asymmetry (λ) and the interaction rate (α). Empirical parameter values are marked by a white circle (STAR Methods).

(F) Experimental measurements of the size of the most mature egg of individual wasps for different nests across the whole duration of the reprogramming process (top) (n = 385 individuals). Exemplary stochastic trajectories (bottom).

(G) Prediction of global activity changes by the model. A t test was performed on the null hypothesis that average activity is equal in control and reprogrammed nests.

significantly, suggesting that such interactions could contribute to the re-establishment of the steady state.

To quantify a potential asymmetry in interactions, we calculated, for each individual, indices representing the frequency of their involvement in aggressive interactions (fight index) and the probability of dominance during these altercations (dominance index). In order to associate these colony-scale indices with those obtained at the molecular scale, we used the reproductive characteristics of each individual by measuring egg development. After classifying the individuals according to their ovary size, the analysis of fight and dominance indices revealed that in control nests, there is a significant asymmetry of interactions between individuals where only individuals with evidence of developed ovaries—typically the queens—are involved in fights and successfully dominate nestmates (Figure 2B). On the other hand, after the removal of the queen, this asymmetry broke down from the eggless phase, where all individuals, regardless of the size of their ovaries, start to interact in an aggressive manner, until phase D4 (Figure 2B). These observations are consistent with the collective upregulation of their queen genes as observed earlier (STAR Methods section; Figure S2E). After D14, the asymmetry in interactions is re-established. Taken together, these experimental results show that the development of ovaries positively correlates with the rate of interactions (fighting index) and with the probability of behavioral dominance (dominance index) in a given interaction event (Hamilton et al., 2016; Turillazzi and West-Eberhard, 1996). Because queen gene expression correlates with ovary development (Figure S2E), this indicates a parallel relationship between queen gene expression and interactions.

Based on these findings, in the simplest mathematical description reflecting these observations, the rate of interactions between two wasps i and j is proportional to their queen gene expression levels, $r_i r_j$. The asymmetry of interactions observed in control nests requires a factor that decreases monotonically with the difference of their queen gene expression, $\sigma(r_i - r_j)$. The function σ decreases monotonically on a scale $1/\lambda$, such that λ is proportional to the degree by which gene expression levels can be mutually discriminated (supplemental theory). As a result, the rate with which individual i is subject to subdominant interactions with individual j is $K(r_i, r_j) = r_i r_j \sigma(r_i - r_j)$. In order to break the molecular symmetry across workers, fighting interactions must have a repressive effect on the expression of queen genes in the subdominant insect, which is supported by our transcriptome results at D14 (Figure 1F) and by previous experimental observations showing inhibition of endocrine activities in subdominant individuals (Röseler et al., 1983). The biological details of such a repression usually involve the prolonged increase of the concentration of repressor molecules, which in our model reduces the rate of queen gene expression. By integrating out the degrees of freedom describing such repressor molecules (supplemental theory), the time evolution of $P(\{r_1, \ldots, r_N\}, t)$ effectively becomes dependent on its history, which in mathematical terms means that the process becomes non-Markovian. However, in the limit of time scales much longer than typical interaction times, the effect of the colony feedback on the molecular state of individual i can be entirely absorbed in J_{col} which can be approximately written as $J_{col} = \mathbb{E}^m \alpha P(r_i, t) \sum_{j \neq i} K(r_i, r_j)$, where the parameter α is the interaction rate per individual and m denotes the average reduction of queen gene products as a result of one interaction. A complete derivation of the model is given in the supplemental theory. Taken together, in this biophysical model (Figure 2C), collective dynamics on the colony level affect queen gene expression on the molecular scale and, vice versa, queen gene expression determines the rate and outcomes of interactions between pairs of individuals on the colony scale.

Antagonistic dynamics on the molecular and colony levels allow the emergence of a single queen

To test the hypothesis that phenotypic specialization and plasticity both result from the combination of upregulation of queen genes and their colony-level repression, we next considered the stochastic dynamics of the entire nest described by the joint probability $P(\{r_1, \ldots, r_N\}, t)$ of finding a given nest composition $\{r_1, \ldots, r_N\}$ at a time t. The time evolution of this probability is governed by contributions stemming from all individuals, $\partial_t P(\{r_1, \ldots, r_N\}, t) = \sum_i (J^i_{mol} + J^i_{col})$, with the molecular- and colony-induced fluxes defined as $J^i_{mol} = \mathbb{E}_i^{-1} P(\{r_1, \ldots, r_N\}, t) + \mathbb{E}_i^{1} r_i P(\{r_1, \ldots, r_N\}, t)$ and $J^i_{col} = \mathbb{E}_i^{m} \alpha P(\{r_1, \ldots, r_N\}, t) \sum_{j \neq i} K(r_i, r_j)$, respectively.

Our calculations and stochastic simulations show that starting from a configuration of individuals with low expression levels of queen genes (workers), the transient absence of repressive interactions leads to a global increase in queen gene expression levels. This is a emergent consequence of the model definition, and it is in agreement with the experimental observation of hormonal and gene expression changes in response to queen removal (Edinger et al., 1997; Hamilton et al., 2016; Röseler, 1977; Röseler and Röseler, 1978). Ultimately, the competition between upregulation on the molecular scale and repression on the population level indeed leads to a bimodal distribution of phenotypes in the population, where queens and workers are clearly distinguished by their molecular states (Figure 2D). Although experimentally the outcome is the emergence of a single queen, the model captures a range of potential asymptotic population structures depending on the interaction rate, α, and the degree of the sensitivity of dominance behavior, λ (asymmetry). More specifically, for weak and symmetric interactions, all wasps attain reproductive phenotypes, whereas for sufficiently frequent and asymmetric interactions, exactly a single queen phenotype is predicted to emerge, without the need for tuning of parameters (Figure 2E).

By design, this model does not aim to describe the full complexity of the biological processes regulating *Polistes* society. However, it nevertheless quantitatively predicts key experimental observations, such as the time evolution of ovary sizes and nest activity during the reprogramming dynamics (Figure S2G) and the non-trivial transient emergence of multiple egg layers just after the reprogramming process (Figures S2F–S2H). In addition to our experimental observations from which we derived the kinetic processes underlying the model, these predictions further support the mechanistic basis of the model.

Specialization and plasticity are simultaneously achieved by distinguishing between intrinsic and extrinsic perturbations

To gain mechanistic insight into how robust specialization and plasticity are simultaneously achieved in the wasp society, it is

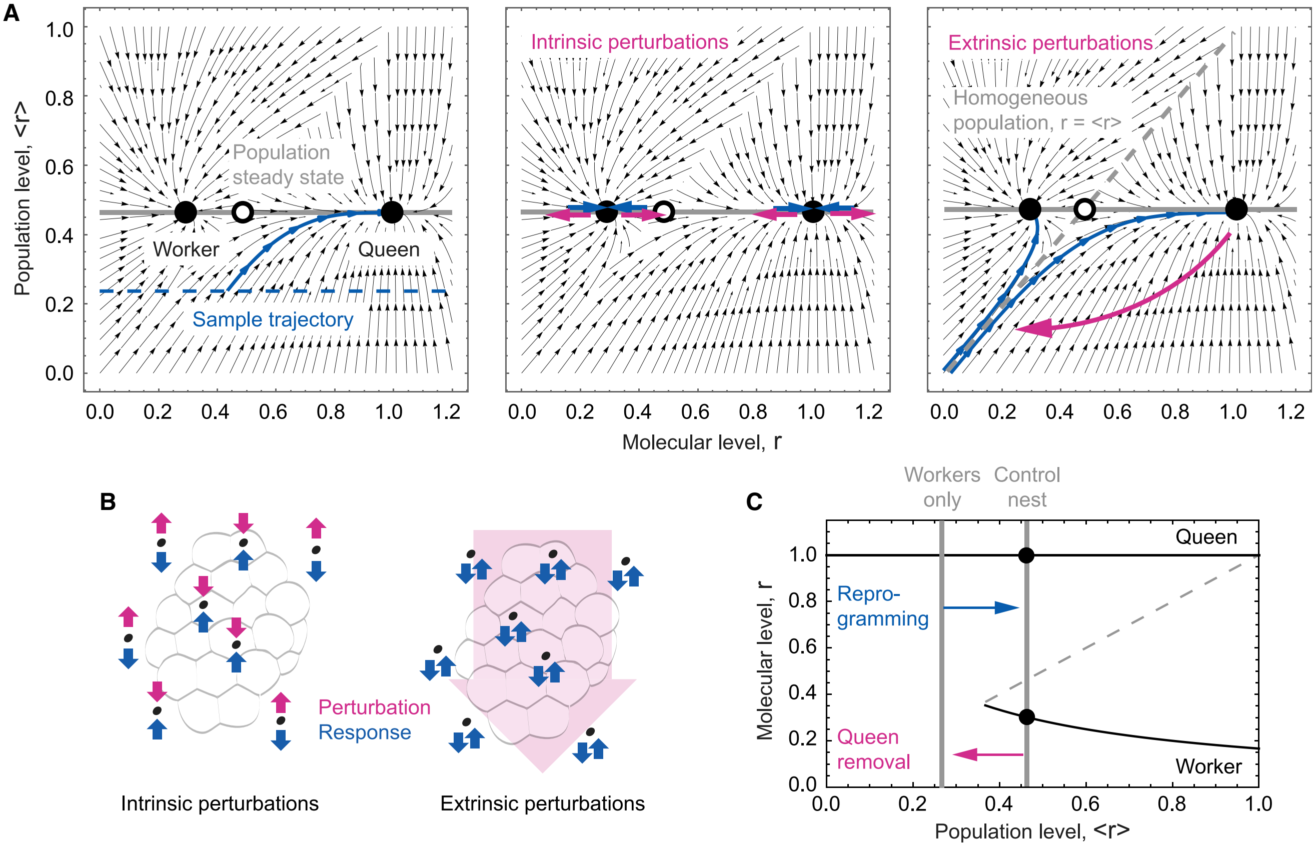

Figure 3. The colony reacts differently to perturbations across scales

(A) Phase portrait depicting the joint dynamics of the population structure $f(r,\tau)$, represented by the average $\langle r \rangle$, and an individual insect, represented by its queen gene expression, r. Left: unperturbed dynamics; middle: intrinsic perturbations; right: extrinsic perturbations. The gray line represents the steady-state average queen gene expression in the colony, the dashed blue line the initial average queen gene expression of a hypothetical queenless population, and the solid blue line the temporal evolution of the queen gene expression level of a particular individual from this colony. In the left and right panels, pink lines denote perturbation and blue lines the ensuing relaxation dynamics.

(B) Schematic depicting the response to intrinsic and extrinsic perturbations.

(C) Bifurcation diagram. Stable attractors are depicted as solid black lines, and unstable states are represented by the dashed gray line.

instructive to consider the dynamics in a mathematical limit where stochastic fluctuations are negligible (mean-field limit). To derive such a description, we considered the time evolution of a single individual embedded into a nest with a fixed composition $P(\{r_1,\ldots,r_N\},t)$ (Hartree approximation) and calculated the conditional probability of finding this individual in a state r for a given nest composition. In this (mean-field) limit, the time evolution of the number of individuals that have dimensionless gene expression level r at dimensionless time τ, $f(r,\tau)$, can be obtained in the limit of time scales much larger than typical interaction times. We find that

$$\partial_\tau f(r,\tau) + \partial_r \underbrace{[(1-r)f(r,\tau)]}_{\text{Molecular scale}} = \underbrace{\tilde{\alpha}\partial_r\left[\int_0^\infty K(r,r')f(r',\tau)\,dr'\right]}_{\text{Population scale}},$$

with interaction kernel $K(r,r') = rr'\sigma(r'-r)$ and rescaled interaction rate $\tilde{\alpha}$ (supplemental theory). This approximation accurately describes the structure of the phase space of the full stochastic dynamics, as validated by comparison to stochastic simulations (Figures S3A and S3B; supplemental theory). The dynamics reach a steady state if processes on the molecular scale (second term on the left-hand side) are balanced by the effect of colony-level feedback (term on the right-hand side). It is therefore instructive to consider the co-evolution of the molecular dynamics—represented by the expression level of queen genes, r, and the population composition, $f(r,\tau)$. To this end, we computed the time evolution of a hypothetical individual with a given expression value of queen genes and the corresponding evolution of the population composition (Figure 3A, left; Video S2). The co-evolution of both scales is represented by black arrows, with an exemplary trajectory highlighted in blue. Our analysis showed that the dynamics relax to a steady-state composition of the population (represented by a specific distribution of queen gene expression values, f, with an average value of $r = (\sqrt{2\tilde{\alpha}+1} - 1)/\tilde{\alpha}$). This steady state comprises two attractors of the microscopic dynamics at $r_2 = 1/(1+\tilde{\alpha}r)$ and $r_0 = 1$, corresponding to the worker and queen phenotype, respectively, and giving

rise to a bimodal population composition as observed in our stochastic simulations (Figure 2D).

Specialized phenotypes are subject to different sources of perturbations: intrinsic perturbations, such as gene expression noise, and extrinsic perturbations, such as the removal of the queen. Intrinsic perturbations by definition affect individuals independently such that the population composition remains unaffected. Such a perturbation leads to an opposing response in queen gene expression dynamics, such that this perturbation is actively suppressed by the interplay between molecular and colony-scale dynamics (Figure 3A, middle, horizontal displacements). Extrinsic perturbations affect all individuals in the society in a correlated manner, perturbing both the population composition, f, and gene expression levels, r. As a result of such perturbations, the system converges to a "plastic" state that separates convergence to the worker and the queen phenotype (separatrix). In this state, individuals have the capacity to evolve toward any of the two attractors and therefore become either worker or a queen (Figure 3A, right, diagonal displacement). Mathematically, the system undergoes a saddle-node bifurcation with the population structure, f, acting as a functional bifurcation parameter (Figures 3B and 3C). Taken together, the central result of this study obtained from the synthesis of our multi-scale experimental and theoretical approaches is that *Polistes* integrates antagonistic dynamics on different scales to distinguish between intrinsic, molecular-level perturbations (which are uncorrelated between individuals) and extrinsic, population-level perturbations (which are correlated between individuals), reacting stably to the former ones and plastically to the latter ones. Therefore, the interplay between dynamics on different biological scales of organization allows *Polistes* to simultaneously achieve robust specialization and rapid plasticity.

DNA methylation may stabilize the social structure against strong fluctuations

The preceding analysis predicts that the steady state is metastable with respect to perturbations that are uncorrelated across insects. The precise time scale of escape from this metastable state depends on the specific rates describing the dynamics on the molecular scale and cannot be rigorously deduced from this mean-field analysis. We will in the following estimate the quantitative value of the time scale of the nest stability considering strong noise. By employing an argument by contradiction, we will show with a simple statistical argument that the empirical time scale of nest stability necessitates processes reducing fluctuations on the molecular level.

A *Polistes* worker is, on average, subject to a subdominant interaction 3.7 ± SEM 1.6 times per day in control nests (Figure S4A). Therefore, interactions occur on a comparable time scale—of the order of hours—to typical gene activation or pheromone production times (Edinger et al., 1997; Hamilton et al., 2016; Röseler, 1977; Röseler and Röseler, 1978). As the ratio, R, of these time scales is of order one, such a situation necessarily leads to the frequent chance activation of queen genes in workers due to the stochastic timing of interactions. For example, a statistical estimation yields an expected queen persistence time $T = T_{int}(R + 1/[1-(1-e^{-R})^N])$, where T_{int} is the average time between two consecutive subdominant interactions of a worker. This queen persistence time would equate to less than three days for a nest of 20 insects and if the molecular state of insects is stable for 30 h after a queen interaction ($R = 4.6$) (Figure S4B; supplemental theory). The presence of the queen in *Polistes* societies is, however, very stable over several weeks (Southon et al., 2015), which would imply that molecular states such as queen gene expression would be stable in the absence of queen interactions significantly longer than 30 h (Figure S4C). This simple statistical estimate suggests that additional factors stabilize wasp societies against such fluctuations.

As in mammals, *Polistes* have additional, epigenetic, layers of gene expression regulation, including chemical modifications of the DNA, such as the methylation of cytosines (DNA methylation) (Patalano et al., 2015; Standage et al., 2016; Jeong et al., 2018). To understand whether DNA methylation could play a role in controlling fluctuations in the *Polistes* society, we analyzed the brain methylomes of all individuals from an unmanipulated control nest (8 individuals). Since the methylomes originated from the same individuals whose transcriptome had been sequenced before, we were able to correlate DNA methylation and gene expression on the level of single genes in individual insects. In line with previous work (Jeong et al., 2018; Zemach et al., 2010), we confirmed that DNA methylation in gene bodies is positively correlated with gene expression levels (Figure S4C). Moreover, notably, we found that DNA methylation in gene bodies is associated with a reduction of gene expression noise as evidenced by a decrease of the fraction of significantly variable genes with increasing DNA methylation levels (Figures 4B, S4D, and S4E), as it has previously been reported in other systems (Huh et al., 2013). As a mathematical consequence of such a reduction in gene expression variance, σ^2, due to DNA methylation, the probability of the chance activation of queen genes decreases stronger than exponentially and therefore necessarily increases the stability of the society by orders of magnitude, $T = T_{int}\{R + 1/[1-(1-e^{-1/\sigma})^N]\}$ (Figure 4C; supplemental theory). This relation holds independently of the model defined above. Hence, DNA methylation is predicted to contribute to stabilizing the colony as a whole. To test this, we compared the methylome of *Polistes* with another primitive social wasp species, *Belanogaster*, which has a similar level of social organization but reduced colony stability (Tindo and Dejean, 2000). Using mass spectrometry to measure overall DNA methylation levels in brains of insects collected in stable nests, we indeed observed significantly lower global DNA methylation levels in *Belanogaster*, which is in agreement with a stabilizing role of DNA methylation (Figure 4D).

If DNA methylation has a long-term stabilizing role for *Polistes* colonies, we would expect that rapid reprogramming after queen removal would require partial removal of DNA methylation marks. We therefore compared the methylomes of all individuals originating from a nest collected in D4 phase (8 individuals) with those collected in the control phase (8 individuals). We observed a partial but significant erasure of global methylation levels (Control: 1.94 ± SD 0.84%, D4: 1.23 ± SD 0.13%, unpaired t test, $p = 0.0335$, Figure 4E). This was evident across all genomic regions and particularly at the level of gene bodies and repetitive elements of the genome (Figure 4F). Consistent with DNA methylation loss, the frequency of genes showing significant expression variability increased after queen removal compared

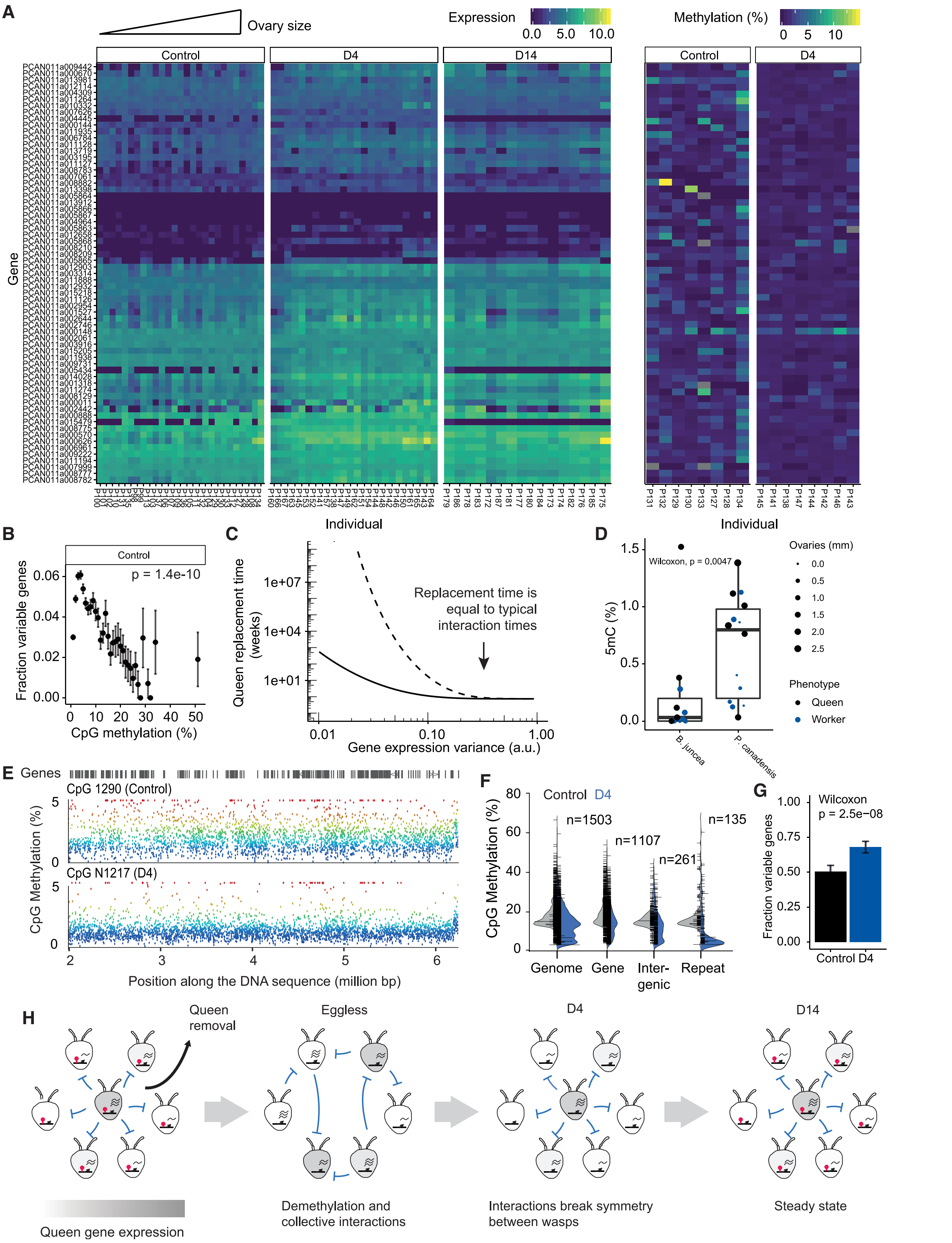

(legend on next page)

with control nests (Figure 4G). To verify that this trend of demethylation was not linked to genetic differences between nests, we validated this result by mass spectrometry by analyzing the global level of DNA methylation of 5 nests from which a subset of individuals was taken before and after queen removal. In each case, we observed a consistently lower rate of DNA methylation in individuals collected during the eggless phase, in which aggressive behaviors are highest (Figure 2B and Figure S5A), confirming the association of DNA methylation levels with the stability of the nest.

Although these observations will require further investigation, in particular for the identification of the mechanisms leading to the depletion of DNA methylation (Figures S5B and S5C) and to establish the reversibility of DNA methylation erasure in *Polistes*, our observations support a role of DNA methylation in stabilizing the *Polistes* society against strong fluctuations.

DISCUSSION

A combination of experimental and theoretical approaches shows that *Polistes* uses antagonistic dynamics on different spatial scales to distinguish between molecular- and colony-level perturbations, thereby achieving robustness to the former and plasticity to the latter. In our approach, we combined molecular profiling using multi-omics with colony-level video recordings in a way that correlates observations on the molecular, individual, and nest scales to inform a biophysical model and a theory that predicts the propagation of perturbations through multiple scales of organization in *Polistes* societies. In our experiments, we studied colonies of *Polistes* in their natural habitat during field work expeditions in Panama. Although studying a social insect in its natural environment limits the use of molecular perturbation techniques available in laboratories, it allowed us to perform behavioral perturbations under natural environmental fluctuations of *Polistes* nests.

Because of its complexity, a model comprising an accurate description of the biological complexity of the processes regulating the insect society would not allow gaining analytical insight into the response of the society to perturbations. We therefore incorporated the minimal set of assumptions that followed directly from our experimental observations. As the behavior of dynamical systems is governed by the stability of their attractors, our main conclusions in Figure 3 are robust with respect to the addition of biological complexities going beyond these assumptions. Importantly, in the spirit of statistical physics, variables constituting the model are therefore to be interpreted as coarse-grained descriptions of the complex processes regulating the society. Our theoretical work then allowed us to fully understand the response of the society on all scales to perturbations on the molecular and societal scale and, consequently, the self-organization processes underlying the simultaneous regulation of specialization and plasticity. These results show how societies of primitively social insects can control how fluctuations propagate across scales of biological organization to perform specific functions. Our results also suggest that DNA methylation seems to play an unanticipated role in regulating the stability of the society at the colony level. Our work demonstrates that correlated measurements across scales can give qualitatively new insights into the mechanisms underlying self-organization of biological systems (Figure 4H). Our approach may be more widely applicable to other biological systems of interest and expanded to more complex societal structures (Sasaki et al., 2016). Our work might also help to understand evolutionary processes on much longer time scales (Menzel and Feldmeyer, 2021), as it provides a unified framework for studying the transition from solitary insects to insect societies.

STAR★METHODS

Detailed methods are provided in the online version of this paper and include the following:

- KEY RESOURCES TABLE
- RESOURCE AVAILABILITY
  - Lead contact
  - Materials availability
  - Data and code availability
- METHOD DETAILS
  - Fieldwork and sample collection
  - Ovaries dissections
  - Video analysis
  - Transcriptome analysis
  - Methylation analysis
  - Biophysical modelling and theory

Figure 4. Epigenetic factors contribute to the stability of the social structure

(A) Heatmap depicting gene expression (left) and DNA methylation levels (right) of queen genes. Genes are ordered based on hierarchical clustering of gene expression in control nests. Individuals are ordered by their ovary size (small to large, left to right), and queens are marked in bold. Only individuals from oriented libraries are shown.

(B) Fraction of genes showing significant biological variability between workers (see STAR Methods) binned by similar DNA methylation levels in control nests. The p value was determined from a Pearson correlation test of the unbinned data.

(C) Theoretical prediction of queen replacements times as a function of gene expression variance across insects for exponentially (solid line) and normally (dashed line) distributed queen gene expression levels.

(D) Global level of DNA methylation measured by mass spectrometry in *Polistes canadensis* (n = 14 individuals) and *Belonogaster juncea* (n = 11 individuals) control nests. Center line corresponds to the median and lower and upper hinges to the 25th and 75th percentiles, respectively. Only nests with at least 2 individuals collected are shown.

(E) Example of global demethylation. Every dot represents average methylation across individuals in a window containing 50 informative CpGs. Color reflects the methylation level of the probe.

(F) Average of CpG methylation levels for different genomic features across 8 individuals collected before (eggless phase) and after (D4 phase) queen removal. Only windows containing 50 informative CpG with at least 10% average methylation in at least one phase are shown.

(G) Fraction of significantly variable genes (adjusted p-value < 0.05, Wilcoxon) in control and early-commitment phase.

(H) Graphical summary of the sequence of events during the relaxation of the nest.

- ○ Parameter estimation
- QUANTIFICATION AND STATISTICAL ANALYSIS

SUPPLEMENTAL INFORMATION

Supplemental information can be found online at https://doi.org/10.1016/j.cels.2022.08.002.

ACKNOWLEDGMENTS

We thank Frank Jülicher, Benjamin D. Simons, and all members of the Reik and Rulands laboratories for helpful discussions. We also thank Nathalie Smerdon at the Wellcome Trust Sanger Institute and Felix Krueger and Simon Andrews at the Babraham Institute for processing sequencing data and bioinformatics support with Illumina sequencing. We thank T. Lengronne, R. Zaurin, R. Southon, and E. Bell for help in the field and all the staff at the Galeta field station and at the Smithsonian Tropical Research Institute Panama for help and logistical support in fieldwork. We also thank P. Vardakas for assistance in the video analyses. This work was conducted under Autoridad Nacional del Ambiente (ANAM) permits #SE/A-33-09, #SE/A-65-10, and #SE/A-20-12 and export permit 10BR004553/DF and 11BR006471/DF. This work was funded by Marie Skłodowska-Curie Individual Fellowship (798082, S.P.), Wellcome Trust (095645/Z/11/Z; W.R.), BBSRC (BB/K010867/1; W.R.), Cancer Research UK (C9681/A18618, C14303/A17197; S.B.), and Wellcome Trust Senior Investigator Award (209441/z/17/z, S.B.), NERC (NE/K011316/1; S.S.). This project has received funding from the European Research Council (ERC) under the European Union's Horizon 2020 research and innovation program (grant agreement no. 950349).

AUTHOR CONTRIBUTIONS

S.P., A.A., S.S., C.G.-R., W.R., and S.R. designed the research. S.P., P.N, and S.D. conducted field work. S.P. conducted wet lab. M.B. and S.B. acquired mass-spectrometry data. S.P., A.A., C.G.-R., I.H.-H., and S.R. analyzed the data. A.A. and S.R. developed the theory. S.P., A.A., C.G.-R., and S.R. developed code for data analysis and numerical simulations. S.P., A.A., W.R., and S.R. wrote the manuscript.

DECLARATION OF INTERESTS

W.R. and S.B. are consultants and shareholders of Cambridge Epigenetix.

Received: August 3, 2021
Revised: April 10, 2022
Accepted: August 5, 2022
Published: August 30, 2022

SUPPORTING CITATIONS

The following references appear in the supplemental information: Assaf, and Meerson, (2017); Oi et al., (2019); Gardiner, (2004); Bauer et al., (2017); Jörg et al., (2021); Rulands et al., (2013); Biró, and Néda, (2018); and Gillespie, (1977).

REFERENCES

Alberts, B., Johnson, A., Lewis, J., Morgan, D., Raff, M., Roberts, K., and Walter, P. (2015). Molecular Biology of the Cell (Garland Science).

Assaf, M., and Meerson, B. (2017). WKB theory of large deviations in stochastic populations. J. Phys. A: Math. Theor. *50*, 263001.

Bachman, M., Uribe-Lewis, S., Yang, X., Williams, M., Murrell, A., and Balasubramanian, S. (2014). 5-Hydroxymethylcytosine is a predominantly stable DNA modification. Nat. Chem. *6*, 1049–1055.

Bauer, M., Knebel, J., Lechner, M., Pickl, P., and Frey, E. (2017). Ecological feedback in quorum-sensing microbial populations can induce heterogeneous production of autoinducers. eLife *6*, 1–38.

Berens, A.J., Hunt, J.H., and Toth, A.L. (2015a). Nourishment level affects caste-related gene expression in Polistes wasps. BMC Genomics *16*, 235.

Berens, A.J., Hunt, J.H., and Toth, A.L. (2015b). Comparative transcriptomics of convergent evolution: different genes but conserved pathways underlie caste phenotypes across lineages of eusocial insects. Mol. Biol. Evol. *32*, 690–703.

Biró, T.S., and Néda, Z. (2018). Unidirectional random growth with resetting. Phys. A *499*, 335–361.

Corona, M., Libbrecht, R., and Wheeler, D.E. (2016). Molecular mechanisms of phenotypic plasticity in social insects. Curr. Opin. Insect Sci. *13*, 55–60.

Cross, M., and Greenside, H. (2009). Pattern Formation and Dynamics in Nonequilibrium Systems (Cambridge University Press).

Davies, J.A. (2013). Mechanisms of Morphogenesis (Elsevier Academic Press).

Edinger, R.S., Mambo, E., and Evans, M.I. (1997). Estrogen-dependent transcriptional activation and vitellogenin gene memory. Mol. Endocrinol. *11*, 1985–1993.

Ferreira, P.G., Patalano, S., Chauhan, R., Ffrench-Constant, R., Gabaldón, T., Guigó, R., and Sumner, S. (2013). Transcriptome analyses of primitively eusocial wasps reveal novel insights into the evolution of sociality and the origin of alternative phenotypes. Genome Biol. *14*, R20.

Gardiner, C.W. (2004). Handbook of Stochastic Methods for Physics, Chemistry, and the Natural Sciences (Springer-Verlag).

Gillespie, D.T. (1977). Exact stochastic simulation of coupled chemical reactions. J. Phys. Chem. *81*, 2340–2361.

Giray, T., Giovanetti, M., and West-Eberhard, M.J. (2005). Juvenile hormone, reproduction, and worker behavior in the neotropical social wasp *Polistes canadensis*. Proc. Natl. Acad. Sci. USA *102*, 3330–3335.

Hamilton, A.R., Shpigler, H., Bloch, G., Wheeler, D.E., and Robinson, G.E. (2016). Endocrine Influences on Insect Societies. In Hormones, Brain and Behavior, D.W. Pfaff, A.M. Etgen, R.T. Rubin, A.P. Arnold, and S.E. Fahrbach, eds. (Elsevier Academic Press), pp. 421–451.

Hänggi, P., Talkner, P., and Borkovec, M. (1990). Reaction-rate theory: fifty years after Kramers. Rev. Mod. Phys. *62*, 251–341.

Herb, B.R., Wolschin, F., Hansen, K.D., Aryee, M.J., Langmead, B., Irizarry, R., Amdam, G.V., and Feinberg, A.P. (2012). Reversible switching between epigenetic states in honeybee behavioral subcastes. Nat. Neurosci. *15*, 1371–1373.

Holman, L., Helanterä, H., Trontti, K., and Mikheyev, A.S. (2019). Comparative transcriptomics of social insect queen pheromones. Nat. Commun. *10*, 1593.

Huh, I., Zeng, J., Park, T., and Yi, S.V. (2013). DNA methylation and transcriptional noise. Epigenetics Chromatin *6*, 9.

Hunt, J.H., Wolschin, F., Henshaw, M.T., Newman, T.C., Toth, A.L., and Amdam, G.V. (2010). Differential gene expression and protein abundance evince ontogenetic bias toward castes in a primitively eusocial wasp. PLoS One *5*, e10674.

Jeong, H., Wu, X., Smith, B., and Yi, S.V. (2018). Genomic landscape of methylation islands in hymenopteran insects. Genome Biol. Evol. *10*, 2766–2776.

Jörg, D.J., Kitadate, Y., Yoshida, S., and Simons, B.D. (2021). Stem cell populations as self-renewing many-particle systems. Annu. Rev. Condens. Matter Phys. *12*, 135–153.

Kennedy, P., Baron, G., Qiu, B., Freitak, D., Helanterä, H., Hunt, E.R., Manfredini, F., O'Shea-Wheller, T., Patalano, S., Pull, C.D., et al. (2017). Deconstructing superorganisms and societies to address big questions in biology. Trends Ecol. Evol. *32*, 861–872.

Menzel, F., and Feldmeyer, B. (2021). How does climate change affect social insects? Curr. Opin. Insect Sci. *46*, 10–15.

Merrell, A.J., and Stanger, B.Z. (2016). Adult cell plasticity in vivo: de-differentiation and transdifferentiation are back in style. Nat. Rev. Mol. Cell Biol. *17*, 413–425.

Miyano, S. (1986). Colony development, worker behavior and male production in orphan colonies of a Japanese paper wasp, Polistes chinensis antennalis Pérez (Hymenoptera: Vespidae). Res. Popul. Ecol. *28*, 347–361.

CellPress
OPEN ACCESS

Ocko, S.A., Heyde, A., and Mahadevan, L. (2019). Morphogenesis of termite mounds. Proc. Natl. Acad. Sci. USA *116*, 3379–3384.

Oi, C.A., Oliveira, R.C., van Zweden, J.S., Mateus, S., Millar, J.G., Nascimento, F.S., and Wenseleers, T. (2019). Do primitively eusocial wasps use queen pheromones to regulate reproduction? A case study of the paper wasp *Polistes Satan*. Front. Ecol. Evol. *7*, 199.

Patalano, S., Hore, T.A., Reik, W., and Sumner, S. (2012). Shifting behaviour: epigenetic reprogramming in eusocial insects. Curr. Opin. Cell Biol. *24*, 367–373.

Patalano, S., Vlasova, A., Wyatt, C., Ewels, P., Camara, F., Ferreira, P.G., Asher, C.L., Jurkowski, T.P., Segonds-Pichon, A., Bachman, M., et al. (2015). Molecular signatures of plastic phenotypes in two eusocial insect species with simple societies. Proc. Natl. Acad. Sci. USA *112*, 13970–13975.

Peleg, O., Peters, J.M., Salcedo, M.K., and Mahadevan, L. (2018). Collective mechanical adaptation of honeybee swarms. Nat. Phys. *14*, 1193–1198.

Pratte, M., Gervet, J., and Theraulaz, G. (1990). Behavioural profiles in *Polistes dominulus* (Christ) wasp societies: a quantitative study. Behaviour *113*, 223–249.

Röseler, P.F. (1977). Juvenile hormone control of oögenesis in bumblebee workers, *Bombus terrestris*. J. Insect Physiol. *23*, 985–992.

Röseler, P.F., and Röseler, I. (1978). Studies on the regulation of the juvenile hormone titre in bumblebee workers, *Bombus terrestris*. J. Insect Physiol. *24*, 707–713.

Röseler, P.F., Röseler, I., Strambi, A., and Augier, R. (1983). Influence of insect hormones on the establishment of dominance hierarchies among foundresses of the paper wasp, *Polistes gallicus*. Behav. Ecol. Sociobiol. *15*, 133–142.

Rulands, S., Klünder, B., and Frey, E. (2013). Stability of localized wave fronts in bistable systems. Phys. Rev. Lett. *110*, 038102.

Sasaki, T., Penick, C.A., Shaffer, Z., Haight, K.L., Pratt, S.C., and Liebig, J. (2016). A simple behavioral model predicts the emergence of complex animal hierarchies. Am. Nat. *187*, 765–775.

Sheng, L., Shields, E.J., Gospocic, J., Glastad, K.M., Ratchasanmuang, P., Berger, S.L., Raj, A., Little, S., and Bonasio, R. (2020). Social reprogramming in ants induces longevity-associated glia remodeling. Sci. Adv. *6*, eaba9869. https://doi.org/10.1126/sciadv.aba9869.

Simola, D.F., Graham, R.J., Brady, C.M., Enzmann, B.L., Desplan, C., Ray, A., Zwiebel, L.J., Bonasio, R., Reinberg, D., Liebig, J., and Berger, S.L. (2016). Epigenetic (re)programming of caste-specific behavior in the ant *Camponotus floridanus*. Science *351*, aac6633. https://doi.org/10.1126/science.aac6633.

Southon, R.J., Bell, E.F., Graystock, P., and Sumner, S. (2015). Long live the wasp: adult longevity in captive colonies of the eusocial paper wasp *Polistes canadensis* (L.). PeerJ *3*, e848. https://doi.org/10.7717/peerj.848.

Standage, D.S., Berens, A.J., Glastad, K.M., Severin, A.J., Brendel, V.P., and Toth, A.L. (2016). Genome, transcriptome, and methylome sequencing of a primitively eusocial wasp reveal a greatly reduced DNA methylation system in a social insect. Mol. Ecol. *25*, 1769–1784.

Strassmann, J.E., Fortunato, A., Cervo, R., Turillazzi, S., Damon, J.M., and Queller, D.C. (2004). The cost of queen loss in the social wasp Polistes dominulus (Hymenoptera: Vespidae). J. Kans. Entomol. Soc. *77*, 343–355.

Strogatz, S.H. (2015). Nonlinear Dynamics and Chaos: With Applications to Physics, Biology, Chemistry, and Engineering (CRC Press).

Sumner, S., Pereboom, J.J., and Jordan, W.C. (2006). Differential gene expression and phenotypic plasticity in behavioural castes of the primitively eusocial wasp, *Polistes canadensis*. Proc. Biol. Sci. *273*, 19–26.

Taylor, B.A., Cini, A., Wyatt, C.D.R., Reuter, M., and Sumner, S. (2021). The molecular basis of socially mediated phenotypic plasticity in a eusocial paper wasp. Nat. Commun. *12*, 775.

Theraulaz, G., Bonabeau, E., and Deneubourg, J.-L. (1995). Self-organization of hierarchies in animal societies: the case of the primitively eusocial wasp *Polistes dominulus* Christ. J. Theor. Biol. *174*, 313–323.

Tindo, M., and Dejean, A. (2000). Dominance hierarchy in colonies of Belonogaster juncea juncea (Vespidae, Polistinae). Insectes Soc. *47*, 158–163.

Todd, E.V., Ortega-Recalde, O., Liu, H., Lamm, M.S., Rutherford, K.M., Cross, H., Black, M.A., Kardailsky, O., Marshall Graves, J.A., Hore, T.A., et al. (2019). Stress, novel sex genes, and epigenetic reprogramming orchestrate socially controlled sex change. Sci. Adv. *5*, eaaw7006. https://doi.org/10.1126/sciadv.aaw7006.

Turillazzi, S., and West-Eberhard, M.J. (1996). Natural History and Evolution of Paper Wasps (Oxford University Press).

van Kampen, N.G. (2007). Stochastic Processes in Physics and Chemistry (Elsevier North-Holland).

West-Eberhard, M.J. (1969). The Social Biology of Polistine Wasps (Miscellaneous Publications Museum of Zoology, University of Michigan).

West-Eberhard, M.J. (2003). Developmental Plasticity and Evolution (Oxford University Press).

Zemach, A., McDaniel, I.E., Silva, P., and Zilberman, D. (2010). Genome-wide evolutionary analysis of eukaryotic DNA methylation. Science *328*, 916–919. https://doi.org/10.1126/science.1186366.

STAR★METHODS

KEY RESOURCES TABLE

REAGENT or RESOURCE	SOURCE	IDENTIFIER
Biological samples		
Polistes canadensis wasps	This paper	N/A
Chemicals, peptides, and recombinant proteins		
RNA Later	Ambion	R0901
Critical commercial assays		
AllPrep DNA/RNA Mini kit	QUIAGEN	80204
NEB Next Kit	New England Biolabs	7103
Imprint DNA Modification Kit	Sigma–Aldrich	MOD50
Deposited data		
Raw sequencing data	This paper	GEO: GSE144409
Polistes canadensis genome	NCBI	GCF_001313835.1
Software and algorithms		
Trim Galore v0.4.1		www.bioinformatics.babraham.ac.uk/projects/trim_galore/
TopHat v2.0.12		https://ccb.jhu.edu/software/tophat/index.shtml
Bismark		https://www.bioinformatics.babraham.ac.uk/projects/bismark/
SeqMonk Version 1.39.0		https://www.bioinformatics.babraham.ac.uk/projects/seqmonk/
RepeatMasker v4.0.1		https://www.repeatmasker.org
R-4.0.2		https://www.r-project.org
Open CV		https://opencv.org
Custom kinetic Monte-Carlo code		https://doi.org/10.5281/zenodo.6582146

RESOURCE AVAILABILITY

Lead contact

Further information and requests for resources and reagents should be directed to and will be fulfilled by the lead contact, Steffen Rulands (rulands@pks.mpg.de).

Materials availability

This study did not generate new unique reagents.

Data and code availability

- Sequencing data and their respective NCBI SRA accession numbers can be found in Table S2.
- Raw data on the size of the ovaries (DataSet_ OvariesDissection.xlsx), measurements of methylation by mass spectrometry (DataSet_ MassSpectrometry.xlsx), and both global (DataSet_GlobalNestActivity.xlsx) and individual (DataSet_IndividualBehaviour.xlsx) behaviour activities are available in the supplementary datasets.
- All original code has been deposited at Github (https://doi.org/10.5281/zenodo.6582146) and is publicly available as of the date of publication. The link is listed in the key resources table.
- Any additional information required to reanalyze the data reported in this paper is available from the lead contact upon request.

METHOD DETAILS

Fieldwork and sample collection

Polistes canadensis

Field experiments were conducted between June and August during 3 expeditions to Panama between 2009 and 2012 in the protected area of Punta Galeta, Colon (9°21’30.877’’N, 79°54’0.0053’’W), under the field collection permits #SE/A-33-09, #SE/A-65-10 and #SE/A-20-12 from the Autoridad Nacional del Ambiente (ANAM) of Panama. Nests were all selected at pre-emergence stages, when only the queen and few co-foundresses were present. Few co-foundresses were present and marked when we initiated the

experiment and none of them had developed mature ovaries during the experiment. All nests were monitored daily in order to mark every new emergent worker and censused every other evening to know the entire nest population. All emergent workers of the *Polistes canadensis* species are mated in early life and then have the potential to become a queen (Turillazzi and West-Eberhard, 1996). The queen was identified by removing an egg and observing who replaced it, as well as from behavioural observations and census data. 26 nests were monitored for three to nine weeks and female wasps representing the different stages of the queen succession were collected. 8 nests were collected unmanipulated (Control phase). Phenotypic reprogramming was induced by removing the queen and an egg in 18 nests and monitored every day to detect any new egg layers. 7 nests were collected before an egglayer appeared (Eggless phase). As soon as a new egg appeared the eggless phase was considered to be over. The identity of this new egg layer was determined over the following day by behavioural observations and then 5 nests were collected (D1 phase). A further 3 nests were monitored and collected 4 to 5 days after the first egg laying occurred (D4 phase). Finally, 3 nests were monitored until 2 weeks after egg laying (D14 phase). All collected nests had similar stage of development (35.6 ±14 days post-emergence) and comparable size (15.6 ± 7.2 individuals) and no sign of parasites or disease. Wasps were collected directly off their nests individually with forceps during the active hours of the day. Their heads were cut off and immediately placed in RNA later solution (Ambion) incubated at 4C overnight to ensure that solution penetrates the brain and kept at -20°C until the dissection of brains. Their bodies were stored in 80% ethanol and kept at -20°C until dissection of ovaries.

Belonogaster juncea

Three nests of *B. juncea* and their 11 individuals were collected in March 2013 in Ebolowa, Cameroon (2°55′N 11°9′E). Collection, storage and reproductive state assessment of all individuals were done similarly than for *P. canadensis*.

Ovaries dissections

Reproductive state was assessed for 385 wasps by measuring the first and biggest egg at the entrance of the oviduct. A mature egg has a size of 1.5 mm, which corresponds to the smallest egg size observed and associated with egg-laying. Ovary size was used a proxy for queen gene expression (*vitellogenin* and *apolipophorin-3*) (Röseler et al., 1983; Giray et al., 2005; Röseler, 1977).

Video analysis

An HD video camera was placed in front of 5 nests during the 2012 fieldwork and video recording was made for at least 30 min during active hours for each phase of the queen removal experiment. Two types of analyses were performed on the videos: quantification of the overall nest activity using computer vision and a classification of wasp behaviours. We use automated machine learning approach to quantify the global movement analysis of the overall nest. Camera movements and unusual captures that might disrupt the quantification were removed. Computer vision analyses (Video S1) were performed by the quantification of the global pixel changes measured between each frame using Python programming language and Open CV (Open Source Computer Vision Library) and normalized by the number of wasps' present. To allow phase and nest inter-comparisons, a normalization of a nest distance with the camera was computed by measuring the diameter of the label used to mark the wasps.

For the individual behavioural analyses, we monitored a total of 400 interactions from 80 individuals and manually classified them into 6 different types of interactions as previously described in *Polistes* (Pratte et al., 1990; Strassmann et al., 2004). The classification was performed manually, reducing the possibility of mis-classification compared to computational approaches. A repetition of the analysis produces results deviating by only 15% from the original analysis. Briefly, DOM: domination over a nestmate (bite, sting, chew, nip or chase). SUB: subordination (in response to DOM or escape). DEC: donor of foraging material to a nestmate. REC: receiver of the DEC. TR: involved in trophallactic exchanges (i.e., exchanges of fluid from one wasp to another). ANT: Involved in antennation. We categorised these types as follows: Fighting as DOM and SUB, foraging as REC and DEC, and neutral interactions as ANT and TR. From this table, Interaction rate (DOM+SUB+DEC+REC+ANT+TR per hour per wasp), Fight index (DOM + SUB / All interactions) and DOM index (DOM/(DOM+SUB)) were calculated for each individual wasp and normalised by the amount of time each wasp was observed on the nest. Wasps present in the nest but not performing any interaction were also included in our analysis. To calculate the rate of subdominant interactions per worker and per hour, i.e., a passive measure of interactions, we considered individuals who were not present in the nest during the video in order to obtain an unbiased measure of this rate. In this specific case, we therefore normalised by the total number of individuals detected in a night-time population census conducted before the video recording.

Transcriptome analysis

Three phases were analysed by RNA-seq (Control n=2 nests, D4 n=3 nests and D14 n=3 nests). The time points were selected in this way to ensure a representation of different stages of the reprogramming process (control: 0 day, D4: ~11 days, D14: ~21 days) and can thus best support the mathematical model. Total RNA was extracted from 87 single brains, using the QIAGENAll PrepDNA/RNAMini Kit according to the manufacturer's instructions. 50 to 200 ng of total RNA was enriched for mRNA using Dynabeads Oligo(dT)25 from Invitrogen in two subsequent steps of purification with fresh beads. Fragmentation was done by incubation of mRNAs for 5 min at 94 °C in the First-Strand Buffer (Invitrogen), and directly followed by cDNA synthesis using a SuperScript III Kit (Invitrogen) according to the manufacturer's instructions. dUTPs were incorporated for second-strand synthesis for library orientation except for individuals originating from nests N1265 and N1283. cDNA was end-repaired, A-tailed, and ligated using the NEB Next Kit (New England Biolabs) according to the manufacturer's instructions. dUTP excision was done before amplification using USER mix (New England Biolabs). Libraries were amplified with 16 cycles using 2× Phusion HF buffer (New England Biolabs). Size selection and

cleaning between steps were performed with the AMPure XP system (Agencourt) to select DNA fragments between 250 bp and 500 bp. Paired-end libraries were sequenced on HiSeq 2500 Illumina platform.

RNA-seq libraries were sequenced on the Illumina HiSeq platform using the default RTA analysis software. RNA-Seq data were trimmed with Trim Galore (v0.4.1, default parameters) and mapped to the *Polistes canadensis* genome assembly GCF_001313835.1 using TopHat v2.0.12 as previously described in (Patalano et al., 2015). Strand specific quantification was performed using RNA-seq pipeline in Seqmonk software Version 1.39.0 (www.bioinformatics.babraham.ac.uk/projects/seqmonk/). To normalize across nests size factors were calculated using the DEseq2 package in R and log transformed with only orientated samples. The list of "queen genes" corresponds to queen-biased genes identified in (Patalano et al., 2015). To identify differentially expressed genes between the control and D4 phase we generated three sets of genes using the DESeq2, EdgeR and intensity difference filters with standard parameters in Seqmonk. P-values were corrected for multiple testing using the Benjamini-Hochberg method and genes were considered differentially expressed if $p<0.05$. Gene set enrichment analysis was performed using a hypergeometric test. To establish the correlation between gene expression and ovary growth we calculated Pearson's correlation coefficient for each gene across all individuals in a given phase. Genes were considered significantly correlated if $p<0.05$ after Benjamini-Hochberg correction for multiple testing.

PCA analysis was performed using the FactoMineR package in R. Genes contributing the most to a given PCA dimension were identified from their cos2. To calculate the correlation coefficients to the queen transcriptomic profile, we computed Pearson's correlation coefficients of gene expression for list of genes associated with the queen phenotype (Patalano et al., 2015) between each individual and the two queens in control nests. Shown in Figure 1F is the average of these two Pearson's correlation coefficients for each individual. To calculate biological variance, after removing control queens from the dataset we fitted a technical noise model using the trendVar function of the scran package (version 1.12.1) and computed noise contributions using scran's decomposeVar function. Genes with p-values smaller than 0.1 after multiple testing correction using the Benjamini-Hochberg method were considered significantly variable.

Methylation analysis

Bisulfite sequencing

Two phases were analysed by BS-seq (Control n=1 nest, D4 n=1 nest).

Genomic DNA was extracted from 16 single brains, using the QIAGEN All Prep DNA/RNA Mini Kit according to the manufacturer's instructions. Between 200 ng and 500 ng of input genomic DNA was used per library and spiked with unmethylated lambda DNA to provide an estimation of BS conversion efficiency. DNA was end-repaired, A-tailed, and ligated with a methylation Adaptor Oligo Kit (Illumina) using the NEB Next kit according to the manufacturer's instructions. The adaptor-ligated DNA was treated with sodium-BS using an Imprint DNA Modification Kit from Sigma–Aldrich according to the manufacturer's instructions for the two-step protocol. BS-treated DNA was amplified using KAPA HiFi Uracil + DNA Polymerase (KAPA Biosystems) with 15 cycles. Size selection and cleaning between steps were performed with an AMPure XP system (Agencourt) to select DNA fragments between 250 bp and 500 bp and sequenced at the Sanger Institute using the HiSeq 2500 Illumina platform.

BS-seq libraries were sequenced on the Illumina HiSeq platform using the default RTA analysis software. Raw sequences were trimmed to remove both poor-quality calls and adapters using Trim Galore (version 0.3.5 with default parameters, www.bioinformatics.babraham.ac.uk/projects/trim_galore/). The remaining sequences were then aligned to genome assembly EVM/PASA (Patalano et al., 2015); using Bismark (version 0.12.2, with the parameters: –bowtie2 –score_min L,0,−0.4) and Genome_build: *Polistes canadensis* GCF_001313835.1 from. Quantification was done in SeqMonk over probes which contain 50 CpGs each with a minimum read count of 4. Only 30pb to 1kbp probes were kept. Intergenic and gene features analysis was done using probes that specifically overlap these features. Repetitive genomic regions were identified using RepeatMasker v4.0.1, using the 20120418 repeat libraries limited to Apocrita species. Methylation over a given feature was calculated by averaging the methylation levels of CpGs across the probes overlapping a given feature. Binomial filter from Seqmonk was applied to identify methylated probes having statistically greater variations than the ones observed globally (using CpGs covered by a minimum of 4 reads). Significance was considered only when probes significantly deviated from the global average by at least 10% after Benjamini-Hochberg correction. GO enrichment analysis was performed with OmicsBox using Fisher's Exact test ($p<0.01$) and reduced to the most specific terms.

Mass-Spectrometry

We used this technique to analyse the overall methylation levels in order to: 1) Validate the intra nest demethylation process (n = 5 nests), 2) Compare the methylation levels between the species *P. canadensis* (n = 6 nests, 14 individuals) and *B. juncea* (n = 3 nests, 11 individuals). Genomic DNA were isolated from all single brains of both species using protocol from (Patalano et al., 2015). For the detection of the methyl groups, five hundred nanograms of genomic DNA was incubated with 5 units of DNA Degradase Plus (Zymo Research) at 37 °C for 3 h. The resulting mixture of 2-deoxy- nucleosides was analysed on a Triple Quad 6500 mass spectrometer (AB Sciex) fitted with an Infinity 1290 LC system (Agilent) and an Acquity UPLC HSS T3 column (Waters), using a gradient of water and acetonitrile with 0.1% formic acid. External calibration was performed using synthetic standards, and for accurate quantification, all samples and standards were spiked with isotopically labelled nucleosides (Bachman et al., 2014).

Biophysical modelling and theory

A detailed and rigorous derivation of the theoretical approach is given in the supplemental theory. Briefly, we defined the minimal stochastic model capable of describing the experimental phenomenology. The evolution of the distribution $P(\{n_i, q_i\}, t)$ of the number of queen gene products, n, and the state of queen gene repression, q, in each individual at a given time t, is given by

$$\frac{d}{dt}P(\{n_i,q_i\},t) = \sum_{i=1}^{N+1}\Bigg\{\mu(1-q_i)[P(\{n_i-1,q_i\},t) - P(\{n_i,q_i\},t)] + \delta[(n_i+1)P(\{n_i,q_i\},t) - n_iP(\{n_i,q_i\},t)] + \Gamma\left(t_i^{int}\right)(1-2q_i)P(\{n_i,1\},t) + \omega(2q_i-1)\sum_{j\neq i}K_{ij}P(\{n_i,0\},t)\Bigg\},$$

where the first two lines account for the production and degradation of queen gene products and the last line contains the terms describing the effect of the population level interactions on gene expression. Having derived the full stochastic non-Markovian dynamics of the joint probability $P(\{n_i,q_i\},t)$, we then proceeded to derive a mean-field description valid in the limits of large time scales and negligible fluctuations that still captures the salient features of the structure of the phase space. This mean-field approximation can be written in integro-differential form as

$$\partial_\tau f(r,\tau) + \partial_r \underbrace{(1-r)f(r,\tau)}_{Molecular\ scale} = \underbrace{\tilde{\alpha}\partial_r\left[\int_0^\infty K(r,r')f(r',\tau)\,dr'\right]}_{Population\ scale}.$$

The mean-field approximation was validated a posteriori by comparison of the mean-field predictions of the dynamics with single realizations of the stochastic process defined by the master equation for the joint probability. Stochastic simulations were performed using custom code implementing Gillespie's algorithm (Gillespie, 1977). Finally, we analysed the influence of gene expression noise on population stability by performing model-free calculations. Our statistical calculations provide an expression relating population-wide gene expression variance to queen replacement times. We refer the interested reader to the supplemental information for details.

Parameter estimation

The interaction kernel $K(r_i, r_j)$ depends on two parameters, the dimensionless interaction rate per individual, α, and the interaction asymmetry λ. To locate the empirical parameter values in the phase diagram we estimated these parameters from the experimental data presented in Figure 2B of the main text. To estimate the interaction rate, we calculated the number of fighting interactions per individual in a time interval *of one day* obtaining an estimate of 3.7 interactions per day. The dimensionless interaction rate, α, is obtained by rescaling this quantity as indicated in the supplemental theory. Additionally, we counted the number of subdominant queen interactions to obtain a lower bound for the interaction asymmetry parameter λ. Out of 17 interactions involving queens in the control and late-commitment phases, we observed 0 interactions where the queen was subdominant. Therefore, the maximum likelihood estimates for the error rate using the beta-distribution as a (conjugate) prior is 1/17. Comparing this result with the analytical calculation of the error rate from the definition of the interaction kernel (supplemental theory) yields a lower bound for the asymmetry parameter $\lambda \approx 24$. We take this lower bound to be the empirical estimate of λ as the interaction kernel is not very sensitive to changes in the asymmetry parameter in this range.

QUANTIFICATION AND STATISTICAL ANALYSIS

Statistical details are given in the figure legends and in Table S3. Statistical details relating to bioinformatics processing of sequencing data is given in the method details section. Non-parametric statistical tests were used when data could not be assumed to be normally distributed. P-values were corrected for multiple testing using the Benjamini-Hochberg method where appropriate. The FDR was controlled at a level of alpha=0.05.

Cell Systems, Volume *13*

Supplemental information

Self-organization of plasticity

and specialization in a primitively social insect

Solenn Patalano, Adolfo Alsina, Carlos Gregorio-Rodríguez, Martin Bachman, Stephanie Dreier, Irene Hernando-Herraez, Paulin Nana, Shankar Balasubramanian, Seirian Sumner, Wolf Reik, and Steffen Rulands

Supplementary Information for:

"Self-organisation of plasticity and specialization in a primitively social insect"

Contents:

Supplementary Figures

Supplementary Tables

Supplemental Theory

Supplementary Figure 1

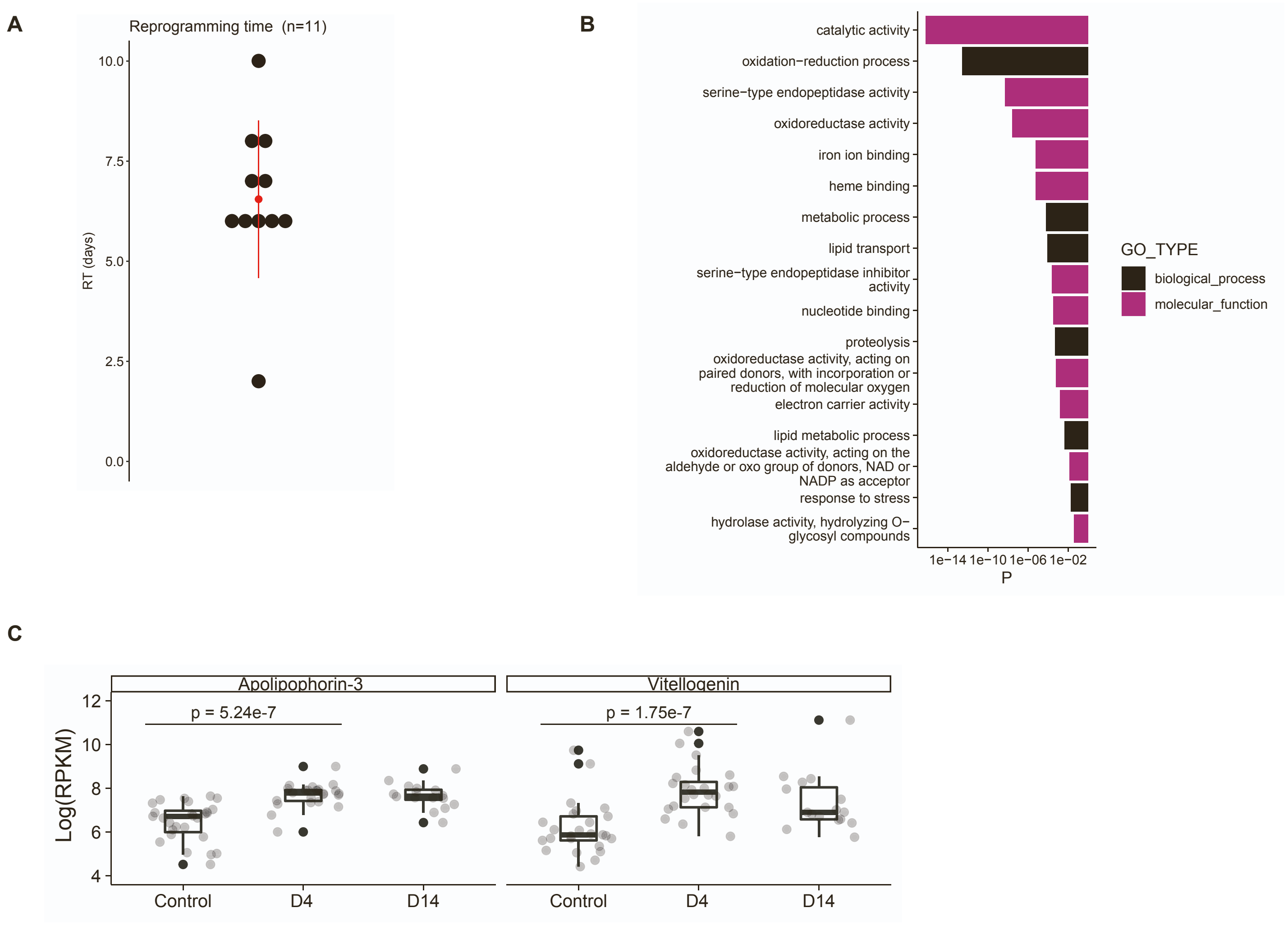

Supplementary Fig. 1.

A, Reprogramming times (i.e. the time taken for a new egglayer to emerge) after queen removal for individual nests (dots). Mean and standard deviation are represented by crossbars. **B,** GO enrichment analysis of the 227 differentially expressed genes. **C,** Expression levels of Vitellogenin and Apolipophorin-3 across the 3 phases (Control, D4, D14). P-values in **b** and **c** were calculated using the R package DESeq2 and corrected for multiple testing using the Benjamini-Hochberg method.

Supplementary Figure 2

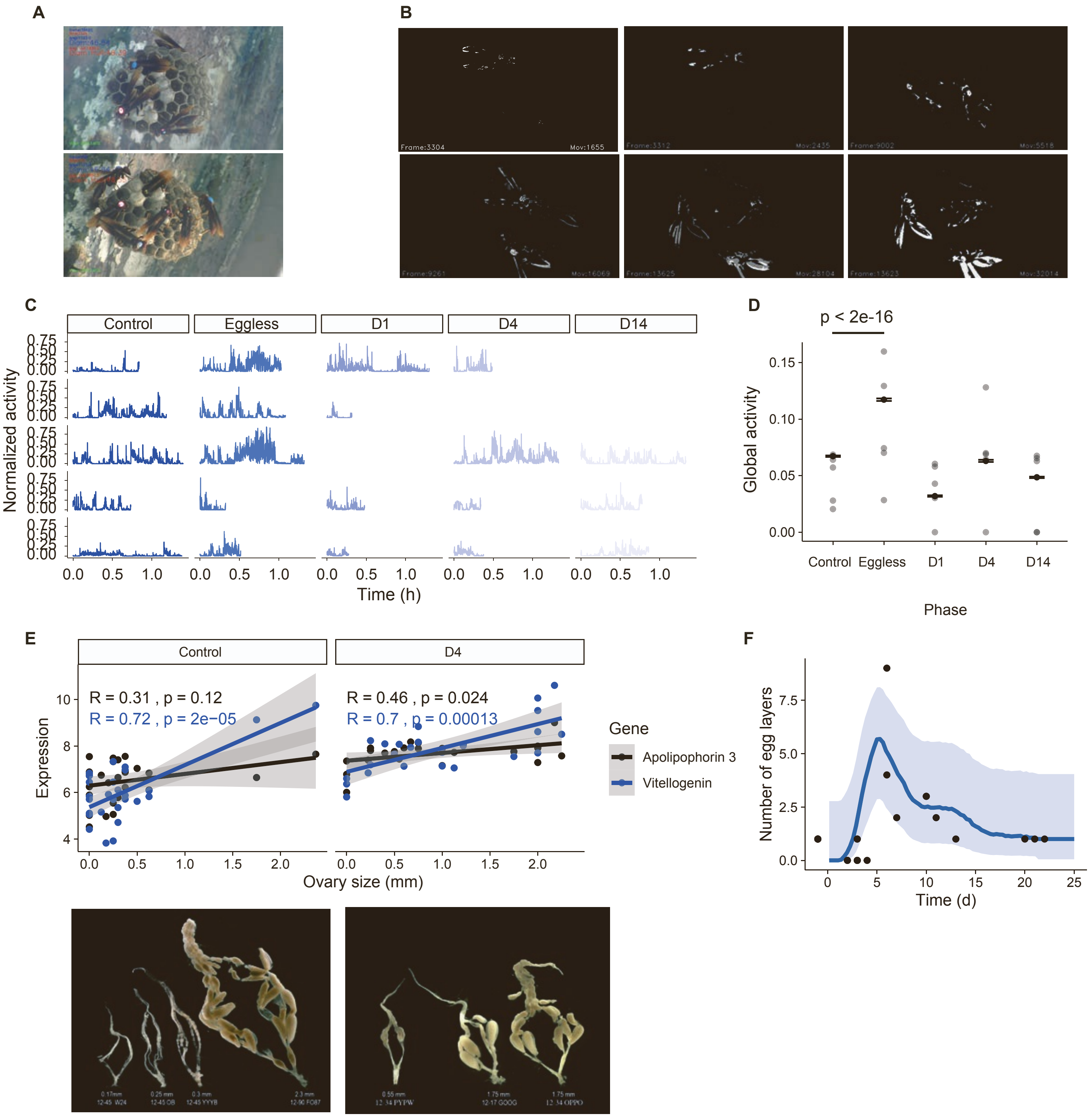

Supplementary Fig. 2.

A, Normalisation using the tag diameter to adjust camera and nest distance. **B,** Example of an increase in global movement detection in the nest N1217 shown by the increase of the amount of white at different time points of the analysis of pixel changes across various frames. 'Frame' indicates the time in which the picture was taken. 'Mov' indicates the number of pixels that changed compared with the previous frame. **C,** Global activity for each nest over time. **D,** Global changes in nest activity. Each grey dot represents a nest. Black dots and error bars correspond to the mean and SEM of all frames. A t-test was performed on the null hypothesis that average activity is equal in control and reprogrammed nests. **E,** Pearson correlation between ovaries development and both *Vitellogenin* and *Apolipophorin-3* genes. Examples of specific ovary dissections for each phase are shown below. **F,** Predicted and measured number of egg layers (ovary size > 1.5mm) over time after queen removal. Shaded area denotes 95% confidence bounds of the theoretical prediction for individual nests.

Supplementary Figure 3

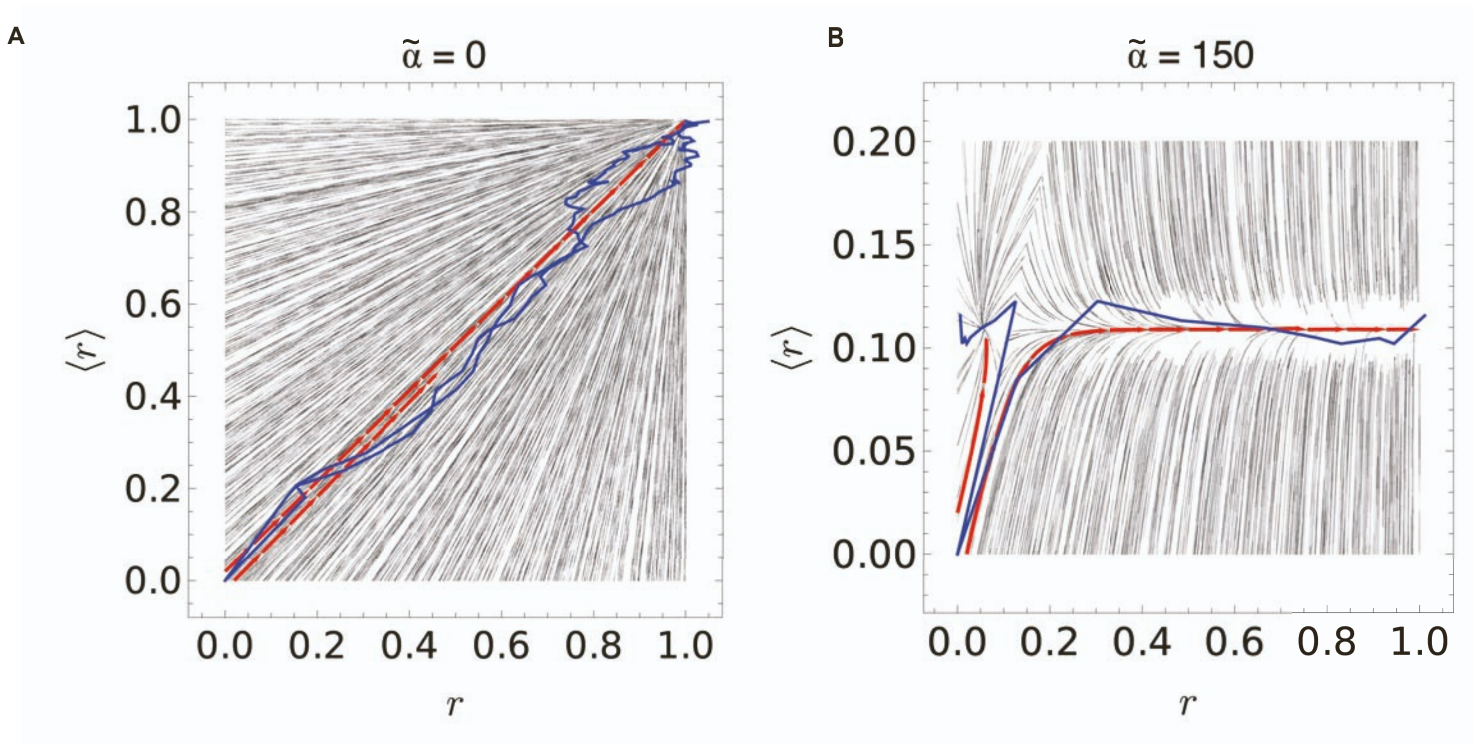

Supplementary Fig. 3.

Phase portrait of the mean-field master equation for values of the interaction rate $\tilde{\alpha}$ corresponding to fully reproductive (**A**) and social **(B)** steady states. The flow lines of the vector field defined by the mean-field master equation are represented by grey lines and two trajectories originating around the origin, corresponding to a nest composed only of individuals lacking queen gene expression, are highlighted in red. Blue lines denote trajectories from numerical simulations of the full stochastic description of the system (Supplemental Theory).

Supplementary Figure 4

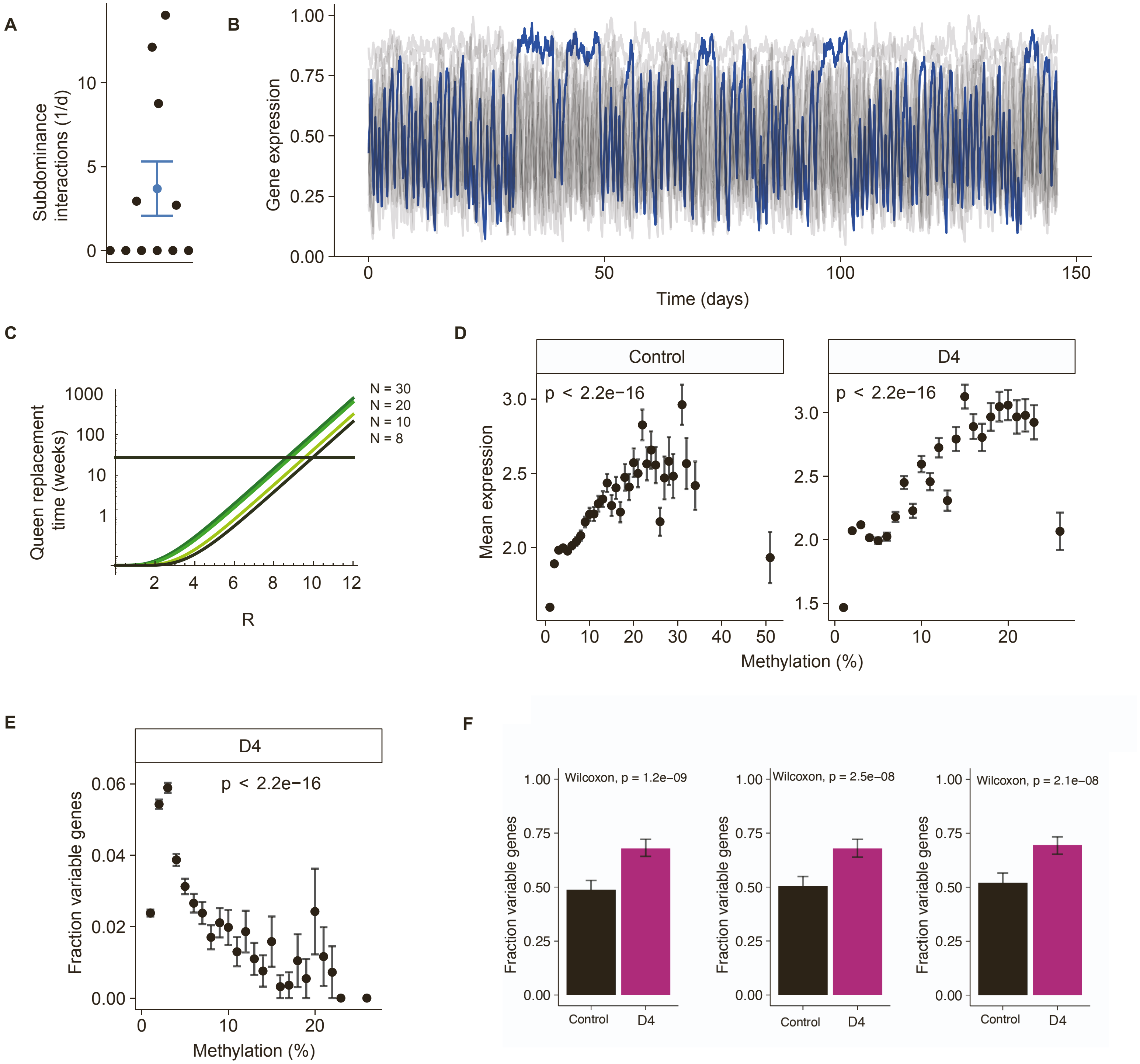

Supplementary Fig. 4.

A, Rate of subdominant interactions per worker and per hour and normalised by the total number of individuals detected during night census (see Methods section). **B,** Sample trajectory obtained from numerical simulations showing the stochastic turnover of queens**. C,** Predicted queen replacement times as a function of the ratio R between interaction and molecular time scales for different values of the population size N. The horizontal line indicates the experimentally measured queen lifespan in *Polistes canadensis* (Supplementary Table 1). **D,** Mean gene expression as a function of the DNA methylation levels in gene bodies. P-values were determined from a Pearson correlation test. **E,** Fraction of significantly variable genes as a function of DNA methylation in the same genes in the early-commitment phase. The p-value was determined from a Pearson correlation test. **F,** Fraction of significantly variable genes in control and early commitment phase for different threshold values of the adjusted p-value: 0.01 (left), 0.05 (centre), 0.1 (right).

Supplementary Figure 5

A

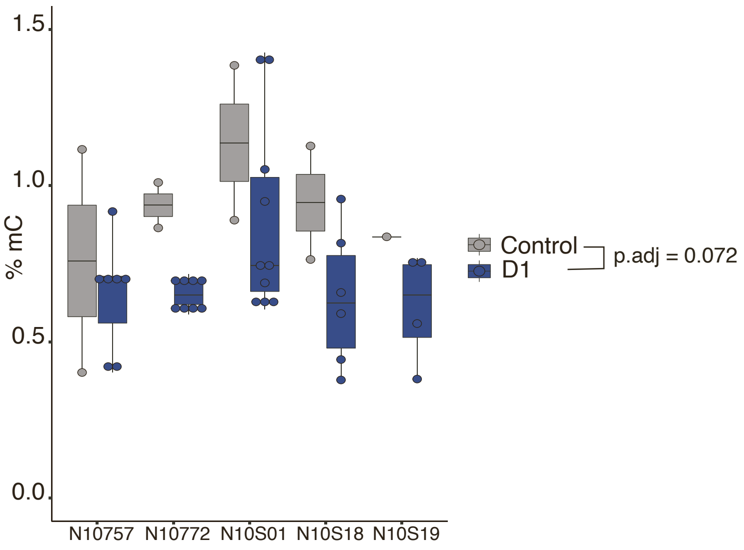

B

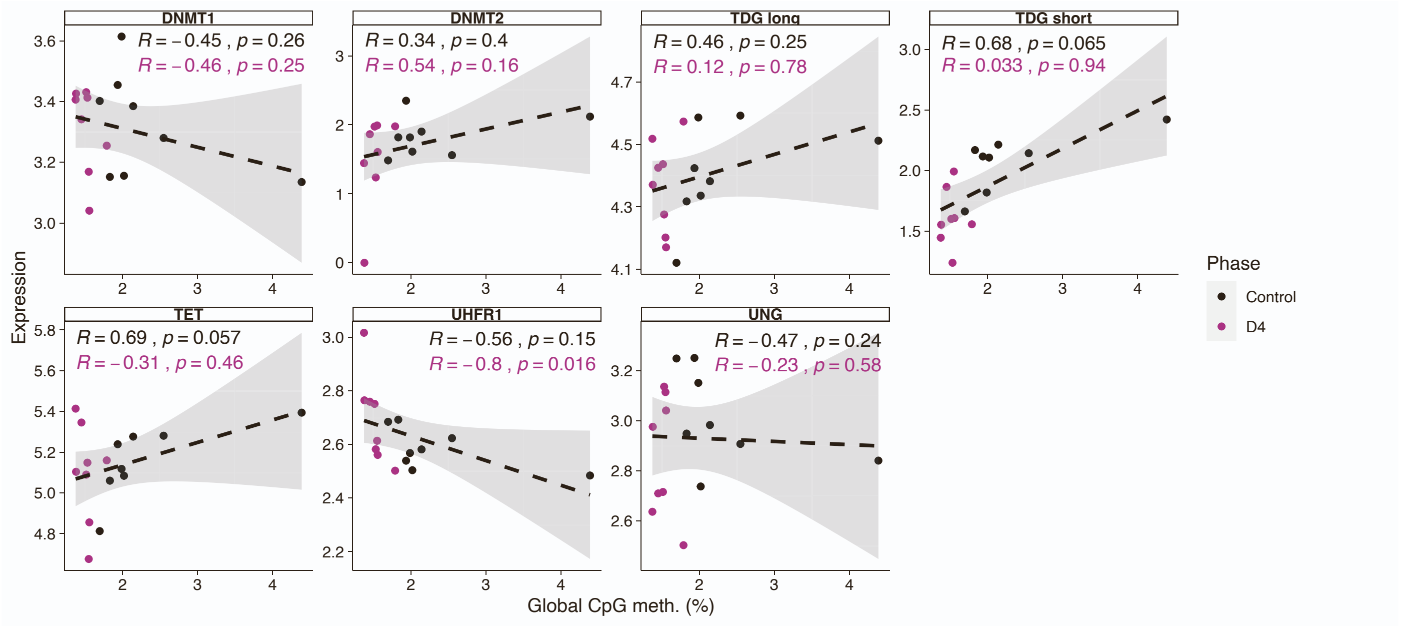

C

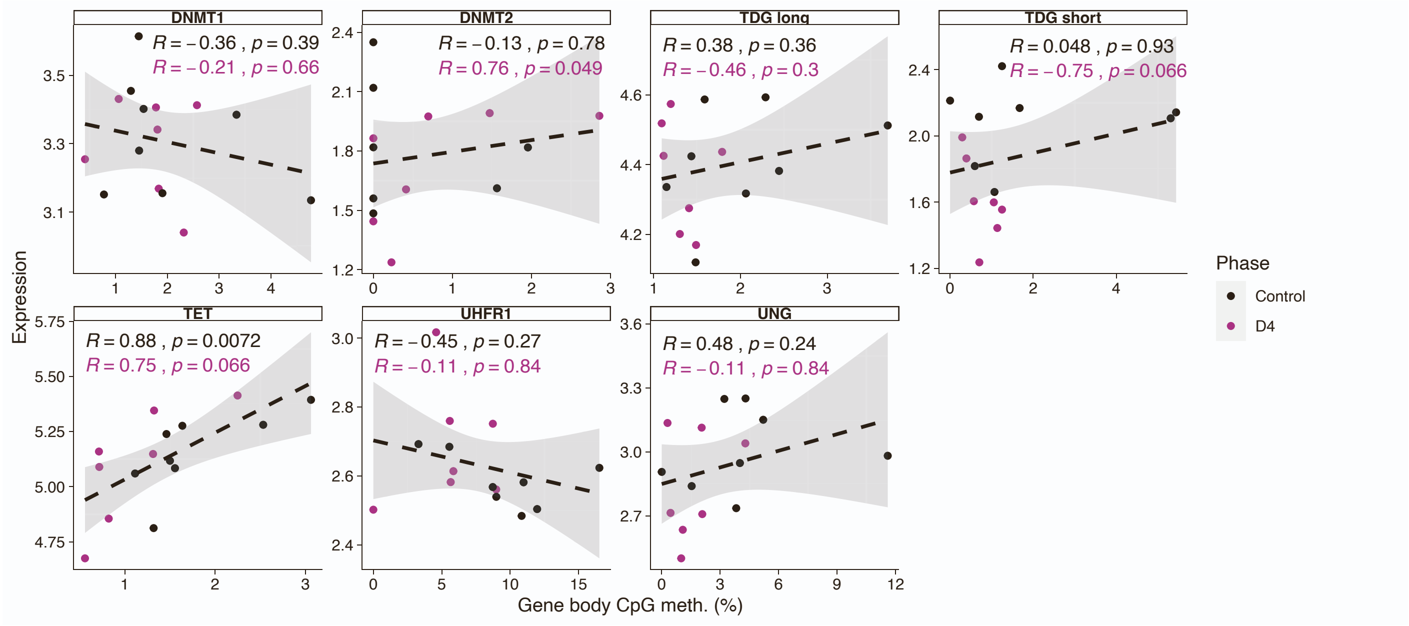

Supplementary Fig. 5.

A, Global level of DNA methylation measured by mass spectrometry in *Polistes canadensis* before and after queen removal (n=44). Center line corresponds to the median and lower and upper hinges to the 25th and 75th percentiles, respectively. P-value was calculated from a Wilcoxon signed rank test between the corresponding phases and corrected for multiple testing using the Holm method. **B, C,** Gene expression of key members of DNA methylation enzymatic machinery[19] as a function of global DNA methylation (B) and gene body DNA methylation (C) in the same genes. R and p denote Spearman's rank correlation coefficient and p-value, respectively, calculated independently for each of the two phases.

Supplementary Tables

Supplementary Table 1.

Number of collected and analysed individuals for each phase of queen removal experiments

Nest	Experiment	Collection	Individual collected	Behavioural - observation	Ovaries dissection	Mass spec methyl	RNA-seq sequencing	BS-seq sequencing
N10789	Control	8-Jul-10	14	N/A	13	0	0	0
N1253	Control	2-May-12	17	N/A	17	0	0	0
N1239	Control	8-May-12	21	N/A	21	0	0	0
N1219	Control	9-May-12	17	N/A	16	0	0	0
N1245	Control	18-May-12	16	N/A	16	0	16	0
N1255	Control	17-Jun-12	12	N/A	12	0	0	0
N1290	Control	17-Jun-12	12	N/A	11	0	11	8
N12101	Control	17-Jun-12	5	N/A	5	5	0	0
N09108	Eggless	20-Jul-09	16	N/A	16	0	0	0
N09143	Eggless	22-Jul-09	12	N/A	12	0	0	0
N09145	Eggless	23-Jul-09	21	N/A	21	0	0	0
N09175	Eggless	17-Jul-09	23	N/A	23	0	0	0
N09178	Eggless	17-Jul-09	17	N/A	15	0	0	0
N09230	Eggless	23-Jul-09	14	N/A	14	0	0	0
N09302	Eggless	23-Jul-09	13	N/A	12	0	0	0
N10757	Control / D1	28-Jun-10	14	N/A	13	9	0	0
N10772	Control / D1	7-Jul-10	45	N/A	43	10	0	0
N10S01	Control / D1	7-Jul-10	15	N/A	14	14	0	0
N10S18	Control / D1	7-Jul-10	10	N/A	10	8	0	0
N10S19	Control / D1	7-Jul-10	6	N/A	6	5	0	0

N1217	D4	27-May-12	10	18	10	0	8	8
N1234	D4	9-Jun-12	13	12	11	0	8	0
N1279	D4	7-Jun-12	11	N/A	10	0	8	0
N1242	D14	9-Jun-12	18	14	18	0	16	0
N1265	D14	13-Jun-12	11	15	11	0	9*	0
N1283	D14	9-Jun-12	15	21	15	0	11*	0
**Total**			**398**	**80**	**385**	**51**	**87**	**16**

* Unoriented sequenced libraries

Supplementary Table 2.

Sequencing data and their respective NCBI SRA accession numbers.

Nest	Experiment	Tag ID	Phenotype	Brain Dissection ID	Sequencing	BioSample	SRA
N1245	Control	OOOW	Worker	P98	RNA-seq	SAMN13938101	SRX7644456
N1245	Control	White	Worker	P99	RNA-seq	SAMN13938025	SRX7644457
N1245	Control	BYBY	Worker	P100	RNA-seq	SAMN13938077	SRX7644382
N1245	Control	W16	Foundress	P101	RNA-seq	SAMN13938076	SRX7644383
N1245	Control	GGGO	Worker	P102	RNA-seq	SAMN13938075	SRX7644384
N1245	Control	W23	Foundress	P103	RNA-seq	SAMN13938074	SRX7644385
N1245	Control	BOO	Worker	P104	RNA-seq	SAMN13938073	SRX7644386
N1245	Control	W64 - Pink	Worker	P105	RNA-seq	SAMN13938043	SRX7644387
N1245	Control	W24	Foundress	P106	RNA-seq	SAMN13938042	SRX7644388
N1245	Control	OB	Worker	P107	RNA-seq	SAMN13938041	SRX7644389
N1245	Control	W19	Queen	P108	RNA-seq	SAMN13938023	SRX7644390
N1245	Control	OBW	Worker	P109	RNA-seq	SAMN13938022	SRX7644391
N1245	Control	GYP	Worker	P110	RNA-seq	SAMN13938021	SRX7644392
N1245	Control	YYYB	Worker	P111	RNA-seq	SAMN13938020	SRX7644393
N1245	Control	W67	Foundress	P112	RNA-seq	SAMN13938019	SRX7644394
N1245	Control	W51 - Pink	Foundress	P113	RNA-seq	SAMN13938018	SRX7644395
N1290	Control	OOBG	Worker	P127	BS-seq	SAMN13938017	SRX7644365

N1290	Control	BPPG	Worker	P128	BS-seq	SAMN13938016	SRX7644366
N1290	Control	PBYY	Worker	P129	BS-seq	SAMN13938015	SRX7644367
N1290	Control	WYBG	Worker	P130	BS-seq	SAMN13938014	SRX7644368
N1290	Control	FO91	Foundress	P131	BS-seq	SAMN13938013	SRX7644369
N1290	Control	OOYG	Worker	P132	BS-seq	SAMN13938012	SRX7644370
N1290	Control	OWGB	Worker	P133	BS-seq	SAMN13938011	SRX7644371
N1290	Control	FO87	Queen	P134	BS-seq	SAMN13938010	SRX7644372
N1290	Control	OOBG	Worker	P127	RNA-seq	SAMEA2061704	ERS227149
N1290	Control	BPPG	Worker	P128	RNA-seq	SAMEA2060493	ERS227150
N1290	Control	PBYY	Worker	P129	RNA-seq	SAMEA2060662	ERS227151
N1290	Control	WYBG	Worker	P130	RNA-seq	SAMEA2061705	ERS227152
N1290	Control	FO91	Foundress	P131	RNA-seq	SAMEA2060660	ERS227145
N1290	Control	OOYG	Worker	P132	RNA-seq	SAMEA2060492	ERS227147
N1290	Control	OWGB	Worker	P133	RNA-seq	SAMEA2060661	ERS227148
N1290	Control	FO87	Queen	P134	RNA-seq	SAMEA2060491	ERS227144
N1290	Control	BPPY	Worker	P135	RNA-seq	SAMEA2060663	ERS227154
N1290	Control	BWWP	Worker	P136	RNA-seq	SAMEA2060494	ERS227153
N1290	Control	PYYY	Worker	P137	RNA-seq	SAMEA1971113	ERS227146
N1217	D4	P91	Foundress	P138	BS-seq	SAMN13938009	SRX7644373
N1217	D4	GYPG	Worker	P141	BS-seq	SAMN13938008	SRX7644374
N1217	D4	GOOG	Worker	P142	BS-seq	SAMN13938007	SRX7644375

N1217	D4	YGGG	Egg Layer	P143	BS-seq	SAMN13938006	SRX7644376
N1217	D4	OWPP	Worker	P144	BS-seq	SAMN13938005	SRX7644377
N1217	D4	P72 (From 12-18)	Foundress	P145	BS-seq	SAMN13938004	SRX7644378
N1217	D4	BPBP	Egg Layer	P146	BS-seq	SAMN13938003	SRX7644379
N1217	D4	Y65 - PPWW	Worker	P147	BS-seq	SAMN13938078	SRX7644380
N1217	D4	P91	Foundress	P138	RNA-seq	SAMN13938040	SRX7644396
N1217	D4	GYPG	Worker	P141	RNA-seq	SAMN13938039	SRX7644397
N1217	D4	GOOG	Worker	P142	RNA-seq	SAMN13938038	SRX7644398
N1217	D4	YGGG	Egg Layer	P143	RNA-seq	SAMN13938037	SRX7644399
N1217	D4	OWPP	Worker	P144	RNA-seq	SAMN13938036	SRX7644400
N1217	D4	P72 (From 12-18)	Foundress	P145	RNA-seq	SAMN13938035	SRX7644401
N1217	D4	BPBP	Egg Layer	P146	RNA-seq	SAMN13938034	SRX7644402
N1217	D4	Y65 - PPWW	Worker	P147	RNA-seq	SAMN13938033	SRX7644403
N1234	D4	OPYW	Worker	P148	RNA-seq	SAMN13938032	SRX7644404
N1234	D4	YOOG	Worker	P149	RNA-seq	SAMN13938031	SRX7644405
N1234	D4	OPPO	Egg layer	P150	RNA-seq	SAMN13938030	SRX7644406
N1234	D4	BPPB	Worker	P151	RNA-seq	SAMN13938029	SRX7644407
N1234	D4	WBYB	Worker	P152	RNA-seq	SAMN13938028	SRX7644408
N1234	D4	YGPP	Worker	P153	RNA-seq	SAMN13938027	SRX7644409
N1234	D4	YYYG	Worker	154	RNA-seq	SAMN13938026	SRX7644410
N1234	D4	PYPW	Worker	P157	RNA-seq	SAMN13938100	SRX7644411

N1279	D4	PBBP	Worker	P160	RNA-seq	SAMN13938099	SRX7644412
N1279	D4	Y11	Egg layer	P161	RNA-seq	SAMN13938098	SRX7644413
N1279	D4	BWB	Worker	P162	RNA-seq	SAMN13938097	SRX7644414
N1279	D4	BGYY=YBYW	Worker	P163	RNA-seq	SAMN13938096	SRX7644415
N1279	D4	PWG	Egg layer	P164	RNA-seq	SAMN13938095	SRX7644416
N1279	D4	PPPP	Worker	P165	RNA-seq	SAMN13938094	SRX7644417
N1279	D4	YWOO	Worker	P166	RNA-seq	SAMN13938093	SRX7644418
N1279	D4	BYBP	Worker	P167	RNA-seq	SAMN13938092	SRX7644419
N1242	D14	Bl	Worker	P172	RNA-seq	SAMN13938091	SRX7644420
N1242	D14	OGBB	Worker	P173	RNA-seq	SAMN13938090	SRX7644421
N1242	D14	YPGP	Worker	P174	RNA-seq	SAMN13938089	SRX7644422
N1242	D14	B59 - BP	Egg layer	P175	RNA-seq	SAMN13938088	SRX7644423
N1242	D14	OOBO	Worker	P176	RNA-seq	SAMN13938087	SRX7644424
N1242	D14	WYGY	Worker	P177	RNA-seq	SAMN13938086	SRX7644425
N1242	D14	WGBB	Worker	P178	RNA-seq	SAMN13938085	SRX7644426
N1242	D14	B10	Foundress	P179	RNA-seq	SAMN13938084	SRX7644427
N1242	D14	WYOY	Worker	P180	RNA-seq	SAMN13938083	SRX7644428
N1242	D14	YWOY	Worker	P181	RNA-seq	SAMN13938082	SRX7644429
N1242	D14	OYOY	Worker	P182	RNA-seq	SAMN13938081	SRX7644430
N1242	D14	W71	Worker	P183	RNA-seq	SAMN13938080	SRX7644431
N1242	D14	OPWO	Worker	P184	RNA-seq	SAMN13938079	SRX7644432

N1242	D14	PPBG	Worker	P185	RNA-seq	SAMN13938123	SRX7644433
N1242	D14	YOY	Worker	P186	RNA-seq	SAMN13938122	SRX7644434
N1242	D14	Bl	Worker	P187	RNA-seq	SAMN13938121	SRX7644435
N1265	D14	BWOB	Worker	P188	RNA-seq	SAMN13938120	SRX7644436
N1265	D14	BGGP	Worker	P189	RNA-seq	SAMN13938119	SRX7644437
N1265	D14	White spot	Foundress	P190	RNA-seq	SAMN13938118	SRX7644438
N1265	D14	WGPP	Worker	P191	RNA-seq	SAMN13938117	SRX7644439
N1265	D14	FY95	Foundress	P192	RNA-seq	SAMN13938116	SRX7644440
N1265	D14	YPBY	Worker	P193	RNA-seq	SAMN13938115	SRX7644441
N1265	D14	Yellow	Egg layer	P196	RNA-seq	SAMN13938114	SRX7644442
N1265	D14	green spot	Foundress	P197	RNA-seq	SAMN13938113	SRX7644443
N1265	D14	Red	Worker	P198	RNA-seq	SAMN13938112	SRX7644444
N1283	D14	GYWW	Worker	P201	RNA-seq	SAMN13938111	SRX7644445
N1283	D14	PPPW	Worker	P202	RNA-seq	SAMN13938110	SRX7644446
N1283	D14	BYOY	Worker	P203	RNA-seq	SAMN13938109	SRX7644447
N1283	D14	PYY	Egg layer	P204	RNA-seq	SAMN13938108	SRX7644448
N1283	D14	G91	Worker	P205	RNA-seq	SAMN13938107	SRX7644449
N1283	D14	GWPY	Worker	P206	RNA-seq	SAMN13938106	SRX7644450
N1283	D14	OYOW	Worker	P208	RNA-seq	SAMN13938105	SRX7644451
N1283	D14	BWWY	Worker	P209	RNA-seq	SAMN13938104	SRX7644452
N1283	D14	YWBB (from 12-42)	Foundress	P210	RNA-seq	SAMN13938103	SRX7644453

N1283	D14	G68	Worker	P211	RNA-seq	SAMN13938102	SRX7644454
N1283	D14	WOGW	Worker	P212	RNA-seq	SAMN13938024	SRX7644455

Supplementary Table 3.
Details of the statistical analysis

**Figure**	**Comparison**	**p-value**	**Adjusted p (BH)**	**Adjusted p (Holm)**	**Number of tests**	**Test type**
1F	Control-D4	7.85*10^-5	2.36*10^-4	2.36*10^-4	2	t-test
1F	Control-D14	0.20	0.20	0.20	2	t-test
2B(top)	Control-Eggless	3.13*10^-6	1.25*10^-5	1.25*10^-5	4	wilcoxon
2B(top)	Control-D1	0.005	0.01	0.015	4	wilcoxon
2B(top)	Control-D4	0.074	0.0987	0.148	4	wilcoxon
2B(top)	Control-D14	0.72	0.72	0.72	4	wilcoxon
2B(mid)	Queen-workers (Control)	0.041	0.0683	0.164	5	wilcoxon
2B(mid)	Queen-workers (Eggless)	0.759	0.759	0.759	5	wilcoxon
2B(mid)	Queen-workers (D1)	0.018	0.0683	0.09	5	wilcoxon
2B(mid)	Queen-workers (D4)	0.041	0.0683	0.164	5	wilcoxon
2B(mid)	Queen-workers (D14)	0.081	0.101	0.164	5	wilcoxon
2B(bot)	Queen-workers (Control)	0.02	0.033	0.06	5	wilcoxon
2B(bot)	Queen-workers (Eggless)	0.465	0.465	0.465	5	wilcoxon
2B(bot)	Queen-workers (D1)	0.214	0.268	0.428	5	wilcoxon
2B(bot)	Queen-workers (D4)	1.16*10^-4	5.8*10^-4	5.8*10^-4	5	wilcoxon

2B(bot)	Queen-workers (D14)	7.58*10^-4	1.90*10^-4	3.03*10^-3	5	wilcoxon
2G	Control-Eggless	<2.2*10^-16	-	-		
4B		1.4*10^-10	-	-		pearson
4D	B.juncea - P.canadensis	4.7*10^-3	-	-		wilcoxon
4G	Control-D4	2.5*10^-8	-	-		wilcoxon
S1C(l)	Control-D4	5.24*10^-7	-	-		t-test
S1C(r)	Control-D4	1.75*10^-7	-	-		t-test
S2D	Control-Eggless	<2.2*10^-16	-	-		t-test
S2E	Vitellogenin (control)	2*10^-5	-	-		pearson
S2E	Apolipophorin 3 (control)	0.12	-	-		pearson
S2E	Vitellogenin (D4)	1.3*10^-4	-	-		pearson
S2E	Apolipophorin 3 (D4)	0.024	-	-		pearson
S4D	Control	<2.2*10^-16	-	-		pearson
S4D	D4	<2.2*10^-16	-	-		pearson
S4E		<2.2*10^-16	-	-		pearson
S5A	Control-D1	0.00716		0.0072		wilcoxon
S5B	DNMT1 (Control)	0.26	-	-		pearson
S5B	DNMT1 (D4)	0.25	-	-		pearson

S5B	DNMT2 (Control)	0.4	-	-		pearson
S5B	DNMT2 (D4)	0.16	-	-		pearson
S5B	TDG long (Control)	0.25	-	-		pearson
S5B	TDG long (D4)	0.78	-	-		pearson
S5B	TDG short (Control)	0.065	-	-		pearson
S5B	TDG Short (D4)	0.94	-	-		pearson
S5B	TET (Control)	0.057	-	-		pearson
S5B	TET (D4)	0.46	-	-		pearson
S5B	UHFR1 (Control)	0.15	-	-		pearson
S5B	UHFR1 (D4)	0.016	-	-		pearson
S5B	UNG (Control)	0.24	-	-		pearson
S5B	UNG (D4)	0.58	-	-		pearson
S5C	DNMT1 (Control)	0.39	-	-		pearson
S5C	DNMT1 (D4)	0.66	-	-		pearson
S5C	DNMT2 (Control)	0.78	-	-		pearson
S5C	DNMT2 (D4)	0.049	-	-		pearson
S5C	TDG long (Control)	0.36	-	-		pearson
S5C	TDG long (D4)	0.3	-	-		pearson

S5C	TDG short (Control)	0.93	-	-		pearson
S5C	TDG short (D4)	0.066	-	-		pearson
S5C	TET (Control)	0.0072	-	-		pearson
S5C	TET (D4)	0.066	-	-		pearson
S5C	UHFR1 (Control)	0.27	-	-		pearson
S5C	UHFR1 (D4)	0.84	-	-		pearson
S5C	UNG (Control)	0.24	-	-		pearson
S5C	UNG (Control)	0.84	-	-		pearson

Supplemental Theory

Adolfo Alsina and Steffen Rulands

In this Supplemental Theory we provide details of the derivation of the mathematical framework underlying the results presented in the main text.

Contents

1 Introduction **3**

2 Model definition **5**

2.1 Dynamics on the molecular level 5

2.2 Interactions between individuals 6

2.3 Coupling between population-level interactions and gene expression . . 8

3 Phase diagram **10**

4 Derivation of the mean-field master equation **12**

4.1 Continuum limit 15

5 Derivation of the phase portrait **16**

6 Stability of the steady state **22**

7 Prediction of experimental data **26**

7.1 Marginal distribution of ovary sizes 26

7.2 Experimental parameters 29

8 Numerical simulations **31**

1 Introduction

The primitive social insect *Polistes canadensis* forms societies composed of a single queen, which is the only reproductive individual, and multiple workers. After removal of the queen the remaining workers are capable of reprogramming and producing a new queen. In this study, we therefore use *Polistes canadensis* as a model system to understand how biological systems form stable structures in noisy environments which can, at the same time, be rapidly remodelled upon specific cues.

In mathematical terms, within the scope of this work, we define *specialisation* to denote situations where the distribution of phenotypes in the steady state of the population exhibits clearly separated modes which are stable on time scales much longer than the intrinsic time scales of the system. Such a scenario is typically the result of effective barriers resulting either explicitly from a predefined potential landscape or implicitly, as entropic barriers, from the kinetic rules governing the dynamics.

Given such a situation, one would naively expect that the reestablishment of the population structure after removal of one of the modes occurs on similar time scales as the lifetime of the metastable steady state [1]. With the term *plasticity* we denote the capability of the population to repopulate a missing mode on a time scale similar to the intrinsic time scales, i.e. much faster than the lifetime of the metastable steady state.

Our approach to understanding specialization and plasticity in the *Polistes* society combines multi-scale experimental measurements with biophysical modelling. Different nests of *Polistes* were monitored in video, their individuals dissected and their brains subjected to multi-modal sequencing (see details in Supplementary Material), both before and at different time points after queen removal. This experimental approach provides information on different levels of biological organisation: the social, individual and molecular scales. Integrating the information provided by these experiments on different scales allows us to understand the mechanistic principles underlying

the robust specialisation and rapid reprogramming in *Polistes* societies.

In this supplement, we provide details of the calculations underlying the derivation and analysis of a multi-scale biophysical model to understand the simultaneous capability for robust specialization and plasticity in the *Polistes* society. In our approach we seek to define the simplest model that capable of describing the experimental phenomenology. Specifically, we do not assume non-linearities unless motivated by experimental observations. Although our model therefore necessarily does not reflect the full complexity of wasp behaviour, such a reductionist approach will allow us to obtain mechanistic understanding underlying the principles governing specialisation and plasticity. Starting from a model that describes the non-Markovian dynamics of the joint probability distribution we derive the time evolution equations for the marginal probabilities of gene expression levels and ovary sizes. We then take the mean-field limit to derive the structure of the phase space.

The structure of this document is as follows: in section 2 using a master equation formalism we construct a model that describes molecular processes taking place in each individual which we then couple through interactions on the population scale. In section 7 we draw on this model to derive the time evolution of experimental observables, such as the distribution of ovary sizes and global nest activity. Then, in section 3, we construct the rich phase diagram as a function of the interaction rate and the interaction sensitivity. In section 4 we derive a mean-field master equation from the full stochastic model describing the time evolution of the individual and collective dynamics. These results are then used in section 5 to build a phase portrait of the system, which we use to understand how specialization and plasticity are regulated. In section 6 we consider the stability of the systems with respect to fluctuations and investigate the stabilising effect of epigenetic processes on the social structure. Finally, in section 8 we describe the numerical implementation of the model.

2 Model definition

2.1 Dynamics on the molecular level

Our RNA-seq analysis showed that genes, which are upregulated in queens compared to workers in control nests (queen genes), are collectively expressed in all workers during the reprogramming process. This suggests, on the one hand, that the expression of these queen genes can be described by a single degree of freedom. We here denote this degree of freedom on the molecular level by n_i, taken to be the total abundance of proteins corresponding to queen genes in individual i. On the other hand, our analysis demonstrates that these queen genes are constitutively expressed in the absence of queen interactions, a finding corroborated by previous studies on individual workers in Polistes and other social insects [2]. Therefore, in the absence of interactions, the dynamics on the molecular level are described by the production and degradation of proteins with rates μ and δ, respectively. The time evolution of the probability of finding protein abundances $\{n_k\}$ in a population of $N+1$ insects, $P(\{n_k\},t)$, is governed by a master equation of the form

$$\frac{d}{dt}P(\{n_k\},t) = \sum_{i=1}^{N+1} \mu\,[P(\{n_i-1\},t) - P(\{n_i\},t)] \\ + \delta\,[(n_i+1)P(\{n_i+1\},t) - n_i P(\{n_i\},t)]\ , \tag{1}$$

where $P(\{n_i \pm 1\},t)$ represents the probability of observing protein levels $\{n_1,\ldots,n_i \pm 1,\ldots,n_{N+1}\}$ across the population. The first two terms describe the Poissonian production of proteins and the remaining terms their degradation. From here on, we will omit the time dependence of $P(\{n_k\},t)$ unless necessary. The ensuing stochastic dynamics give rise to a steady state characterised by a distribution of protein abundances with an average value of μ/δ. Therefore, in the absence of interactions, the dynamics converge to a steady state where all individuals express queen genes.

2.2 Interactions between individuals

To break the symmetry between individuals and select a single queen the dynamics on the molecular level need to be coupled to a collective process on the population scale. In the context of a nest, individuals interact in different ways, including fighting. These fighting interactions are directed, meaning that in every interaction there is a dominant individual, the attacker, and a subdominant one.

The rate with which individual i is subject to a subdominant interaction with individual j, K_{ij}, can be written as the total interaction rate between both, a_{ij}, times the conditional probability b_{ij} that individual i is subdominant in such an interaction, $K_{ij} = a_{ij}b_{ij}$. To calculate a_{ij} we note that according to our video recordings, which we correlated with ovary size measurements in individual insects, the interaction rate of an individual insect increases with ovary size and the expression level of queen genes, $a_i \equiv \sum_{j \neq i} a_{ij} \propto n_i$. The pairwise interaction rate therefore is proportional to the probability that both individuals interact in a given time interval, $a_i a_j$, times the probability that this interaction involves individuals i and j, $2/[N(N-1)]$, $a_{ij} = 2a_i a_j/[N(N-1)] \propto 2n_i n_j/[N(N-1)]$, such that we set $a_{ij} = \omega n_i n_j$ where ω is proportional to $2/[N(N-1)]$. To derive the conditional probability that individual i is subdominant, b_{ij}, we resort to previous work showing that the outcome of an interaction is strongly determined by the concentrations of Insect Juvenile Hormone (JH), a hormone involved in ovaries development [3]. Indeed, in our video recording of control nests we found that, in an interaction between two individuals the subdominant one is with high probability the one with the smaller ovaries. Given that ovary size is a proxy for queen gene expression, we take the conditional probability of an individual being subdominant to depend on the gene expression difference between the two interacting individuals, $b_{ij} = b(n_j - n_i)$.

Taken together, the conditional probability of i being the subdominant individual

in an interaction between i and j is

$$b(n_j - n_i) = \Theta(n_j - n_i)\,. \tag{2}$$

The total rate of subdominant interactions that individual i receives then takes the form

$$\omega \sum_{j \neq i} K(n_i, n_j)\,, \tag{3}$$

with the interaction kernel being defined by

$$K(n_i, n_j) = n_i n_j \Theta(n_j - n_i)\,. \tag{4}$$

In order to derive the above form of the interaction kernel we have assumed that individuals can accurately measure the gene expression levels of the insect they are interacting with. A more realistic approach is to take into account that sensing of gene expression levels is associated with an uncertainty. If this uncertainty is distributed following a normal distribution with zero mean and variance σ^2, the probability of individual i being subdominant in an interaction with individual j is

$$\int_{-\infty}^{\infty} \frac{1}{\sqrt{2\pi\sigma^2}} e^{\eta^2/2\sigma^2} \theta(n_j - n_i + \eta) d\eta = \frac{1}{2}\left[1 + \mathrm{Erf}\left(\frac{n_j - n_i}{\sqrt{2\sigma^2}}\right)\right]\,, \tag{5}$$

with the error function defined as $\mathrm{Erf}(x) = \int_0^x e^{-y^2}\, dy$. With this the interaction kernel reads

$$K(n_i, n_j) = \frac{n_i n_j}{2}\left[1 + \mathrm{Erf}\left(\frac{n_j - n_i}{\sqrt{2\sigma^2}}\right)\right]\,, \tag{6}$$

where σ^2 determines how precisely an individual can measure gene expression of other individuals. The above expression is difficult to manipulate both numerically and analytically due to the presence of the error function. It is, however, well approximated

by another sigmoidal function

$$K(n_i, n_j) = n_i n_j \frac{e^{-\lambda(n_i - n_j)}}{1 + e^{-\lambda(n_i - n_j)}} , \tag{7}$$

with $\lambda = 2\sigma^{-1}$ denoting the sensing sensitivity.

2.3 Coupling between population-level interactions and gene expression

How do interactions on the population scale get translated into changes on the molecular scale? An interaction leads to the transient increase in the concentration of factors influencing queen gene expression in the subdominant individual. While the precise nature of the pathways and molecular species involved in this process are not fully known in *Polistes*, to break the symmetry between insects and to obtain a single queen they must counteract the gene expression dynamics and thus can only be repressive. This reasoning is also supported by previous studies in *Polistes* showing that inhibition of fertility-linked compounds requires physical interactions [4]. In the following, we therefore refer to these factors as *queen gene repressors.* Based on this, we make a minimal set of assumptions to describe the coupling between interactions and gene expression dynamics:

1. Interactions lead to the transient presence of queen gene repressors in the subdominant individual and
2. these factors, while present, inhibit the expression of queen genes.

Not knowing the precise nature of the molecular pathways triggered by an interaction we model these repressive factors as a binary variably, $q_i \in \{0, 1\}$, representing the absence or presence of repressive factors in individual i, respectively. In our model, queen gene repressors inhibit the expression of queen genes which mathematically translates to a vanishing rate of the production of queen gene products. Therefore, the production rate of queen gene products reads $\mu(1-q_i)$. The dynamics of the repressive

factors themselves are comprised of two processes: queen gene repressors are activated in the subdominant individual upon an interaction ($q_i = 1$) and then persist for a time drawn from a distribution $\Gamma(t)$. If the activity of queen gene repressors involves sufficiently many steps Γ will be approximately normally-distributed with a variance much smaller than the mean. We therefore set $\Gamma(t) \propto \delta(t_{\rm per} - t)$, where $t_{\rm per}$ is the typical persistence time of queen gene repressors.

With this in mind we can now mathematically define the time evolution of the joint probability $P(\{n_k, q_k\})$. To this end, let us consider a population of $N+1$ individuals, each with two degrees of freedom: the number of queen gene products, n_k, and the state of presence of repressive factors, q_k. Taken together, the stochastic dynamics is described by in terms of the non-Markovian master equation of the form

$$\begin{aligned}\frac{d}{dt}P(\{n_k, q_k\}) = \sum_{i=1}^{N+1}\Bigg\{&\mu(1-q_i)\left[P(\{n_i - 1, q_i\}) - P(\{n_i, q_i\})\right] \\ &+ \delta\left[(n_i+1)P(\{n_i+1, q_i\}) - n_i P(\{n_i, q_i\})\right] \\ &+ \Gamma(t_i^{\rm int})P(\{n_i, 1\})(1-2q_i) + \omega\sum_{j\neq i}K_{ij}P(\{n_i, 0\})(2q_i - 1)\Bigg\},\end{aligned} \tag{8}$$

where $t_i^{\rm int}$ is a realisation of a stochastic process defined by the time elapsed since the last subdominant interaction of individual i evaluated at time t. Equation (8) accounts for the full stochastic dynamics of the population, encompassing gene expression dynamics and the dynamics of the repressive factors. Rescaling the number of gene products and time appropriately, we obtain the master equation in dimension-

less form,

$$\frac{d}{d\tau}P(\{r_k,q_k\}) = \sum_{i=1}^{N+1}\Big\{(1-q_i)\,[P(\{r_i-\epsilon,q_i\})-P(\{r_i,q_i\})]$$
$$+\,[(r_i+\epsilon)P(\{r_i+\epsilon,q_i\})-r_iP(\{r_i,q_i\})]$$
$$+\,\Gamma(\tau_i^{\mathrm{int}})P(\{r_i,1\})(1-2q_i)$$
$$+\,\alpha\frac{\mu}{\delta}\sum_{j\neq i}K(r_i,r_j)P(\{r_i,0\})(2q_i-1)\Big\}, \tag{9}$$

where $\alpha = \omega/\delta$ is the interaction rate in units of the degradation time, dimensionless time $\tau = \mu \cdot t$, rescaled queen gene expression levels $r_k = \delta n_k/\mu$ and $\epsilon = \delta/\mu$.

3 Phase diagram

In order to gain insight into the range of possible behaviours of the system we performed stochastic simulations of the dynamics defined by Equation (9) as described in section 8. We scanned the phase space of the system as a function of two parameters that control the coupling between the molecular and population scales: the interaction rate, α, and the sensitivity, λ. For a given combination of these parameters we sampled 50 independent trajectories of the stochastic nest dynamics defined in Equation (9). We set the population size to $N = 10$ individuals and $\tau_{\mathrm{per}} = 1$. We took averages over the the asymptotic ovary size (for a definition see section 7) at a time long after the steady state had been reached, $\tau = 60$. The results are shown in Figure 2e of the main text.

In this phase diagram, we identify three regimes based on the value of the asymptotic number of queens, defined here as those individuals whose gene expression value is larger or equal than 0.8, which corresponds to 80% of the steady state value. In the limit $\alpha \ll 1$ interactions occur on a much slower time scale than the molecular

processes, effectively uncoupling the molecular and the population scales and leading to a steady state where all individuals obtain a queen phenotype. On the other hand, for $\alpha \gg 1$, the asymptotic composition of the society depends on the sensitivity, λ: If λ is small, $\lambda \ll 1$, subdominant interactions affect all individuals with roughly equal probability. As a result, we observe that the asymptotic dynamics converges to a state represented solely by individuals with low expression of queen genes (workers).

On the other hand, if the scale defined by the sensitivity λ is at least of equal order than the typical variability of queen gene expression values, i.e. individuals are able to distinguish phenotypically relevant changes in gene expression, $\lambda > 1$, interactions break the symmetry between individuals and a bimodal social steady state arises. While multiple queens can exist for precisely defined values of α, exactly one single queen is guaranteed to emerge as long as the interaction rate exceeds a threshold value and individuals are capable of distinguishing "macroscopic" gene expression states. Therefore, by integrating antagonistic dynamics on different spatial scales, *Polistes* societies establish a single queen robustly for a large range of parameters, avoiding the need for fine-tuning of parameters that fluctuate in time and from nest to nest.

To locate the empirical parameter values in the phase diagram we estimated the parameters from the experimental data presented in Figure 2b of the main text, obtaining $\alpha_{\mathrm{exp}} \approx 1$. To estimate λ we counted the number of subdominant queen interactions. Out of 17 interactions involving queens in the control and late-commitment phases we observed 0 interactions where the queen was subdominant. The maximum likelihood estimate for the error rate using the beta-distribution as a (conjugate) prior therefore is 1/17. Using the definition of the interaction kernel we find an analytical expression for the error rate,

$$2\int_{-\infty}^{0} d\Delta r(1+\exp(-\lambda\Delta r))^{-1} = 2\ln 2/\lambda$$

Solving for λ we find that $\lambda \approx 24$. According to the phase diagram, these parameters indeed lead to the emergence of a single queen in support of the proposed paradigm.

4 Derivation of the mean-field master equation

The model defined in Equation (9) predicts many of the features of the reprogramming process and properties of the steady state. But its high dimensionality and non-Markovianity render it unsuitable for analytical treatment. In order to understand how specialization and plasticity are simultaneously achieved in the *Polistes* society we start from Equation (9) and develop a continuum, mean-field description. We will then employ this continuum description in section 5 to explore the structure of the phase space as a function of the individual and collective degrees of freedom.

To begin, we consider the time evolution of a single "tracer" individual embedded in a nest with a given composition $P\left(\{r_i, q_i\}_{i=1}^{N}\right)$ and study the evolution of the probability of finding the tracer in the state (r, q) given the nest composition, $P(r, q) \equiv P(r, q|\{r_i, q_i\})$. In this approach we consider the nest as a "bath", which is not affected by the tracer and which determines the fluctuations of the individual tracer dynamics. The master equation for the time evolution of the queen gene expression level and repressor state of the tracer individual reads

$$\begin{aligned}\frac{d}{d\tau}P(r,q) &= (1-q)\left[P(r-\epsilon,q) - P(r,q)\right] \\ &+ \left[(r+\epsilon)P(r+\epsilon,q) - rP(r,q)\right] \\ &+ \Gamma(\tau^{i}_{int})P(r,1)(1-2q) + \alpha\frac{\mu}{\delta}\sum_{j=1}^{N} K(r,r_j)P(r,0)(2q-1)\,. \end{aligned} \tag{10}$$

To obtain a time evolution equation for the marginal probability $P(r)$ we first need to integrate out the queen gene repressors variable, q. To this end, we formally define a trajectory dependent time (cf. Figure 1) as

$$\tilde{\tau}(t) = \int_0^{\tau} d\tau' \prod_{i\in\mathcal{I}} f(\tau_i - \tau')\,, \tag{11}$$

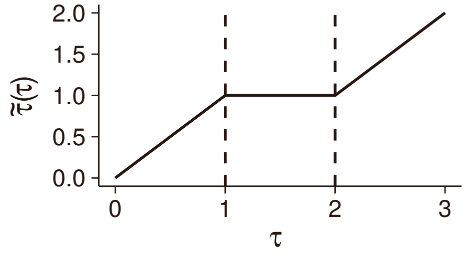

Figure 1: Example depicting the dependence of the trajectory dependent time $\tilde{\tau}$ as a function of the physical time. $\tilde{\tau}$ grows as the same rate as the physical time τ unless an interaction takes place (in this case at $\tau = 1$), in which case it remains at the same value for a time τ_{per}.

where $\{\tau_i\}_{i\in\mathcal{I}}$ is the set of times when an interaction, and hence a $q = 0$ to $q = 1$ transition, takes place, and the function $f(\tau_i - \tau)$ is defined as

$$f(\tau_i - \tau) = \begin{cases} 0 & \tau \in [\tau_i, \tau_i + \tau_{per}] \\ 1 & \text{otherwise}\,. \end{cases} \tag{12}$$

From this definition it follows that $\tilde{\tau}$ increases in the same manner as τ if and only if $q = 0$ and is constant otherwise. The evolution of the system in the new time coordinate $\tilde{\tau}$ thus coincides with the evolution with respect to τ when $q = 0$ and its effect is collapsed to a single time point when $q = 1$. This is equivalent to considering dynamics where $q = 0$ for all times and where a suitably chosen number, M, of queen gene products are instantaneously degraded at times $\tau \in \{\tau_i\}_{i\in\mathcal{I}}$. M is the typical number of molecules degraded in a time interval of length τ_{per}, $M \approx r[1 - \exp(-\tau_{\text{per}})]$. The master equation describing such dynamics takes the form

$$\begin{aligned} \frac{d}{d\tilde{\tau}}P(r) &= [P(r-\epsilon) - P(r)] \\ &+ [(\epsilon + r)P(r+\epsilon) - rP(r)] \\ &+ \alpha\frac{\mu}{\delta}\sum_{j=1}^{N}\left[K(r+M, r_j)P(r+M) - K(r, r_j)P(r)\right]\,. \end{aligned} \tag{13}$$

Next, we consider time scales much longer than typical interaction times. To derive a continuum description, we rescale the persistence time of the repressive factors $\tau_{\rm per}$ in such a way that the average number of degraded queen gene products after an interaction $M(\tau_{\rm per})$ is, on average, equal to a queen gene product unit, ϵ. We simultaneously keep the total effect of repressive interactions in a long time interval $[\tau_1, \tau_2]$ on the concentration of queen gene products, $\int_{\tau_1}^{\tau_2} d\bar{\tau}' M(\bar{\tau}_{\rm per}) \prod_{i\in\mathcal{I}} \delta(\bar{\tau}_i - \bar{\tau}')$, invariant. To this end, the above constraint dictates a corresponding rescaling of the interaction rate, α. Intuitively, such a coarse-graining operation corresponds to a homogeneous distribution of interaction events in the time domain for sufficiently long time scales. As the total effect of interactions on long time scales remains unchanged the structure of the phase portrait remains unchanged as well. With this, we obtain

$$\begin{aligned}\frac{d}{d\tilde{\tau}} P(r) = {} & [P(r-\epsilon) - P(r)] \\ & + [(r+\epsilon)P(r+\epsilon) - rP(r)] \\ & + \alpha' \sum_{j=1}^{N} [K(r+\epsilon, r_j)P(r+\epsilon) - K(r, r_j)P(r)]\end{aligned} \tag{14}$$

where $\alpha' \approx \alpha\mu M/\delta$ is the rescaled interaction rate. The mean-field master equation is then obtained by setting, $x = r/\Omega$, and performing a Kramers-Moyal expansion to the lowest order [5]. For simplicity retaining the symbol r to represent queen gene expression, we find

$$\partial_{\bar{\tau}} r = \tilde{\alpha}_1(x) = 1 - r - \tilde{\alpha} \sum_{j=1}^{N} K(r, r_j)\,, \tag{15}$$

where

$$\tilde{\alpha}_1(r) = \Omega^{-1} \int_{-\infty}^{\infty} dr'(r'-r) W(r'|r) \tag{16}$$

is the first jump moment and $\tilde{\alpha} = \alpha'/\Omega^2$. We henceforth refer to the dual version of Equation (15) describing the evolution of the probability density as the mean field

master equation,

$$\partial_{\tilde{\tau}} P(r) + \partial_r \left[(1-r)P(r)\right] = \tilde{\alpha}\partial_r \left(P(r) \sum_{j=1}^{N} K(r, r_j) \right) . \tag{17}$$

4.1 Continuum limit

We next we take the continuum limit of Equation (15) on the number of individuals, $N \to \infty$, to obtain the time evolution of queen gene expression levels in the "tracer" wasp,

$$\partial_t r = 1 - r - \tilde{\alpha} \sum_{i \neq j} K(r, r_j) = 1 - r - \tilde{\alpha} \int_0^{\infty} \sum_{j=1}^{N} K(r, r')\delta(r' - r_j) \tag{18}$$

$$1 - r - \tilde{\alpha} \int_0^{\infty} \sum_{j=1}^{N} K(r, r')\delta(r' - r_j) \xrightarrow[N \to \infty]{} 1 - r - \tilde{\alpha} \int_0^{\infty} K(r, r') f(r') dr' \tag{19}$$

where the empirical distribution function $f(r') \equiv \sum_j \delta(r_j - r')$ represents the fraction of individuals in the nest having queen gene expression between r and $r + dr$, as well as the time evolution of the population composition,

$$\partial_{\tilde{\tau}} f(r) + \partial_r \left[(1-r)f(r)\right] = \tilde{\alpha}\partial_r \left(f(r) \int_0^{\infty} K(r, r') f(r') dr' \right) . \tag{20}$$

This represents the mean-field description of Equation (9) which is valid in the limit of large populations and time scales, i.e. in the steady state. As we will discuss below, while Equation (19) and Equation (20) are not suitable for quantitatively describing the reprogramming dynamics they nevertheless are capable of providing mathematical insight into the mechanisms underlying the regulation of specialisation and plasticity. It is interesting to note that Equation (20) is conceptually similar to equations described in other biological contexts, such as quorum-sensing bacteria [6] or Mitogen competition by stem cells [7]. In this context, it is also worth to note that in

spatially structured systems specialisation can be achieved by spatially separating different phenotypes, such as via the Turing mechanism, spinodal decomposition, lateral inhibition or via external signalling gradients [8, 9]. If the spatially homogeneous state is unstable such systems are naturally "plastic".

The dynamics on the molecular scale are coupled through a collision-like functional that represents the effect of repressive interactions. In the next section we will study how such a coupling gives rise to specialization and plasticity as emergent properties of the system.

5 Derivation of the phase portrait

The individual and collective dynamics derived in the previous section provide a mean-field description of the system. In Equation (20) the time evolution of $f(r, \bar{\tau})$ is governed by a term describing the molecular dynamics (second term on the left hand side) and a term describing the collective behaviour on the population scale (term on the right hand side). The equation gives rise to a steady state when the molecular dynamics is balanced by the population-level feedback. To understand the relaxation dynamics to the steady state, and its stability, it is instructive to consider the co-evolution of the molecular scale, given by the queen gene expression level r, and the population scale, represented by the distribution $f(r, t)$. In this section we will illustrate the results of our analysis by means of a phase portrait of the multi-scale dynamics. Although the limits we take, such as taking the mean-field limit, do not accurately reflect the full biological complexity, our approximations are validated by comparison to simulations of the full stochastic dynamics.

The starting point of our analysis are Equation (19) and Equation (20), describing the individual and collective dynamics, respectively. Taken together, these equations describe the co-evolution of the queen gene expression level of an individual and the population structure. Stable fixed points of such dynamics represent possible pheno-

types in the society, such as queen and workers. We calculate these fixed points from the intersection of the nullclines of the system. These nullclines are given by

$$0 = 1 - r - \tilde{\alpha} \int_{-\infty}^{\infty} K(r, r') f(r') dr' \tag{21}$$

$$\partial_r \left[(1-r) f(r,t)\right] = \tilde{\alpha} \partial_r \left(f(r,t) \int_{-\infty}^{\infty} K(r, r') f(r', t) dr' \right) . \tag{22}$$

Equation (21) and Equation (22) provide the basis for understanding the steady state of the system as a function of the population composition and the fixed points of the individual dynamics. Although the population composition is represented by a probability distribution, f, its functional nature complicates intuitive interpretations of the relaxation dynamics. In order to obtain a more intuitive picture, we reduce the system to a two-dimensional system describing the coupled evolution of r and the first moment of the population composition, $\langle r \rangle$. The starting point of this approximation is Equation (19),

$$\partial_{\tilde{\tau}} r = 1 - r - \tilde{\alpha} \int_{0}^{\infty} r r' \Theta(r' - r) f(r') dr' \,, \tag{23}$$

describing the evolution of the molecular degree of freedom of a tracer individual. The integral in the right hand side of the equation represents the effect of the interactions received by the tracer individual. In the limit of long times compared with the typical interaction time scale, $t \gg \alpha^{-1}$, the effect of interactions can be approximated by the overall effect of interacting with an effective individual with gene expression level $\langle r \rangle$,

$$\partial_{\tilde{\tau}} r \approx 1 - r - \tilde{\alpha} r \langle r \rangle \Theta(\langle r \rangle - r) \,, \tag{24}$$

where $\langle r \rangle = \int_0^\infty r f(r,t) dr$. Further, by multiplying Equation (20) by r and integrating over r, we obtain the time evolution of the first moment,

$$\partial_{\tilde{\tau}} \langle r \rangle = 1 - \langle r \rangle - \tilde{\alpha} \int_{0}^{\infty} dr \int_{0}^{\infty} dr' K(r, r') f(r) f(r') \,, \tag{25}$$

where $\int_0^\infty dr \int_0^\infty dr' K(r,r')f(r)f(r')$ is the total interaction rate at time t in the nest. To close the system of equations we approximate $\int_0^\infty dr \int_0^\infty dr' K(r,r')f(r)f(r') \approx \langle r\rangle^2/2$ where the factor 2 ensures arises due to double counting of subdominant interactions. Finally, our reduced system of equations reads

$$\partial_{\tilde{T}} r \approx 1 - r - \tilde{\alpha} r \langle r\rangle \Theta(\langle r\rangle - r)\,, \tag{26}$$

$$\partial_{\tilde{T}} \langle r\rangle \approx 1 - \langle r\rangle - \tilde{\alpha}\frac{\langle r\rangle^2}{2}\,. \tag{27}$$

Equation (26) and Equation (27) form a two-dimensional system of equations that is amenable for a bidimensional graphical representation given by a phase portrait. The solutions of these equations in the steady state provide the fixed points of the dynamics. In the steady state we find,

$$\langle r\rangle_0 = \frac{\sqrt{2\tilde{\alpha}+1}-1}{\tilde{\alpha}}\,. \tag{28}$$

For $\tilde{\alpha} = 0$ Equation (26) and Equation (27) admit only one stable solution corresponding to high queen gene expression levels, $r_0 = 1$ (Figure 2). For $\alpha > 0$, we find three solutions if

$$\langle r\rangle > \frac{\sqrt{4\tilde{\alpha}+1}-1}{2\tilde{\alpha}}\,, \tag{29}$$

which in the steady state is always fulfilled for $\tilde{\alpha} > 0$. These three solution comprise two stable branches, at $r_0 = 1$ and $r_2 = 1/(1+\tilde{\alpha}\langle r\rangle)$ and an unstable branch at $r = \langle r\rangle$. Formally, the system therefore comprises a saddle node bifurcation with the population structure as a bifurcation parameter. Intrinsic perturbations, which do not change the value of $\langle r\rangle$, are therefore suppressed by the bistable dynamics in the steady state. Substituting the population steady state,$\langle r\rangle_0$ we obtain as intersections

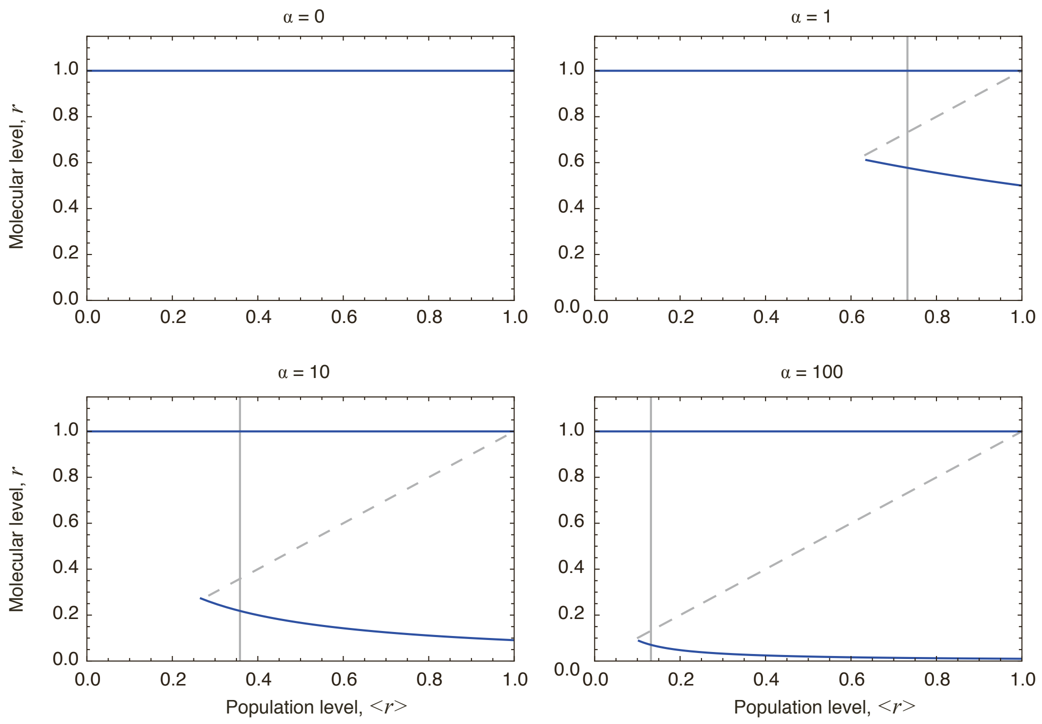

Figure 2: Bifurcation diagram showing fixed points of Equation (26) as a function of $\langle r \rangle$. Stable branches are represented by solid lines and unstable branches by dashed lines. Vertical lines denote the nullclines of Equation (27). For $\tilde{\alpha} > 0$ the dynamics undergo a saddle node bifurcation with the population composition, $\langle r \rangle$, as a bifurcation parameter.

of the nullclines for $\tilde{\alpha} > 0$,

$$
\begin{aligned}
r'_0 &= 1\,, \\
r'_1 &= \frac{\sqrt{2\tilde{\alpha}+1}-1}{\tilde{\alpha}}\,, \\
r'_2 &= \frac{1}{\sqrt{2\tilde{\alpha}+1}}\,.
\end{aligned}
\tag{30}
$$

The flow of the system towards the steady state is represented in Figure 3 and in Figure 3a of the main text. In this representation the x axis corresponds to the molecular degree of freedom and the y axis to the population composition, represented by the first moment of the distribution, $\langle r \rangle$. A population in this portrait is represented

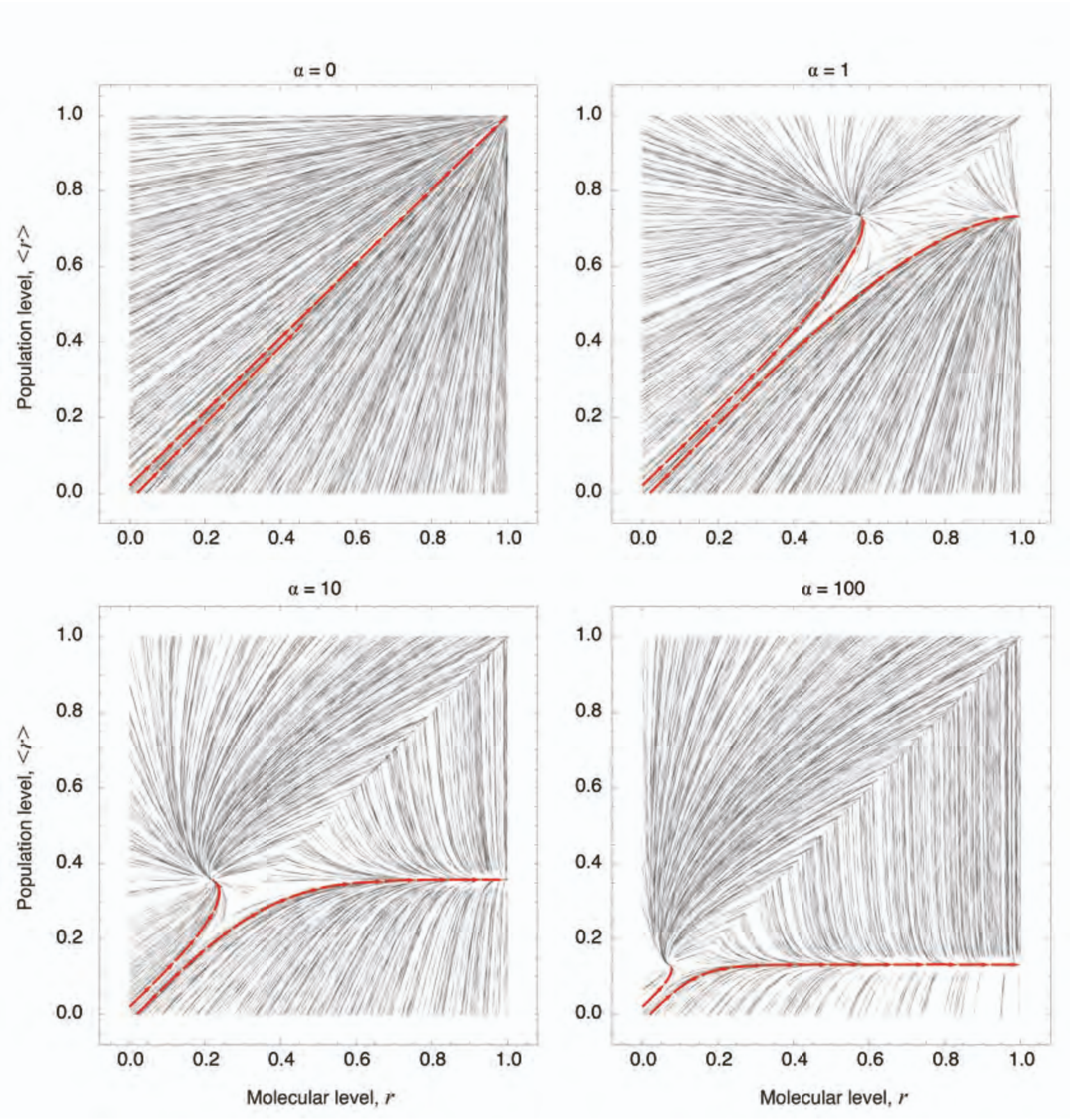

Figure 3: Phase portraits of Equation (26) and Equation (27) for different values of $\tilde{\alpha}$. Random trajectories are represented by gray lines and two trajectories originating around the point $(0, 0)$ corresponding to a nest with only individuals lacking queen gene expression are highlighted in red.

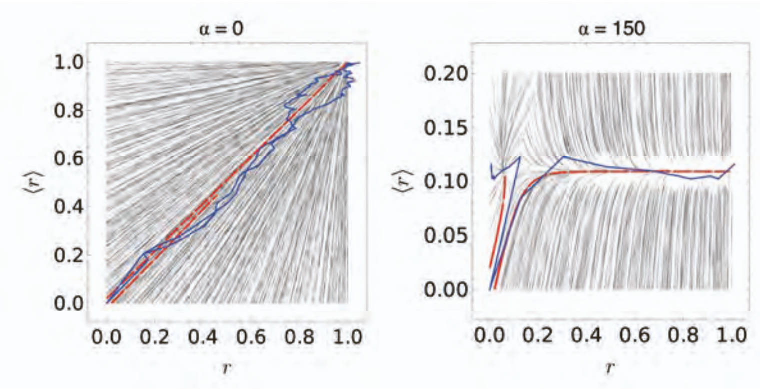

Figure 4: Phase portraits of Equation (26) and Equation (27) for different values of $\tilde{\alpha}$. As in Figure 3 random trajectories are represented by gray lines and two trajectories originating around the point $(0, 0)$ corresponding to a nest with only individuals lacking queen gene expression are highlighted in red. Blue lines denote trajectories from stochastic simulation of the full stochastic system, Equation (9).

by a set of N points, each of them with a different value of the x coordinate, but all of them with the same value of the y coordinate. Changes in the molecular degree of freedom are reflected in changes in the population composition that again affect all individuals identically.

The flow in the mean-field limit qualitatively represents the flow obtained from stochastic simulations of Equation (9) as shown in Figure 4. Therefore, while our calculations are strictly only valid in the limit of an infinite population size and on long time scales, Equation (26) and Equation (27) accurately represent the qualitative structure of the deterministic phase space of Equation (9).

After the removal of the queen the population obtains a homogeneous structure narrowly distributed around the separatrix defined by $r = \langle r \rangle$ and $r = \langle r \rangle \ll 1$. From these initial conditions, the dynamics evolve rapidly along the separatrix. On this separatrix fluctuations can drive each individual either to the queen attractor or to the worker attractor.

6 Stability of the steady state

In section 5 we have analyzed the fixed points of the dynamics of the system and studied their stability against uncorrelated and correlated perturbations. To this end, we employed a mean-field approximation effectively neglecting fluctuations arising due to the finite number of individuals and stochasticity in interaction times, Equation (19) and Equation (20). We will ask how the stability of the nest and the social order, manifest in a clear separation of queen and worker phenotypes, can be maintained in the presence of strong fluctuations. Indeed, typical population sizes range between 8 to 30 individuals and interactions occur on similar time scales as typical protein degradation times, suggesting that fluctuations stabilise the steady state of the society.

To quantitatively understand how fluctuations affect the stability of the society we begin by estimating the persistence time of the society given typical time scales of molecular and population level processes. To this end, we first calculate the probability that any individual upregulates its queen genes in the time interval between two consecutive interactions, and thereby destabilised the society, by producing a sufficient number of queen gene products. If interaction events are statistically independent the waiting time T between consecutive interactions follows an exponential distribution, $P(T) = \omega e^{-\omega T}$. Denoting the time scale of molecular processes (e.g. the time needed for an individual to upregulate the queen genes) by T_δ, the probability that T is longer than T_δ is

$$P(T > T_\delta) = \int_{T_\delta}^{\infty} P(T)dT = e^{-\omega T_\delta} \,. \tag{31}$$

Thus, the probability of a single worker being subject to a repressive interactions in a time interval of length T_δ is $1 - e^{-\omega T_\delta}$. In a nest with N workers, the probability of all of them being subject to a repressive interaction in a time interval T then is $\left(1 - e^{-\omega T_\delta}\right)^N$. Conversely, the probability that at least one worker is capable of

upregulating its queen genes between two consecutive interactions is

$$p = 1 - \left(1 - e^{-R}\right)^{N} \ , \tag{32}$$

where $R = \omega T_{\delta}$ is a dimensionless parameter that represents the ratio between the molecular and the interaction time scales. The number of interactions before an insect is capable of upregulating its queen genes follows a geometric distribution with mean $1/p$. Therefore, the persistence time of the society, τ. is

$$\tau = \omega^{-1} \left(p^{-1} + R\right) \ . \tag{33}$$

The theoretical prediction given by Equation (33) indicates that in order to obtain a queen turnover time consistent with the experimentally measured value of roughly 27.5 weeks (Supplementary Table 1) the ratio between molecular and interaction time scales, R, must be larger or equal than 9 (Supplementary Fig. 4c). Given the experimental estimation of the time interval between two consecutive interactions, this would imply that gene expression states are stable for at least three days in the absence of interactions. However, transcriptional queen signatures are already established in *Polistes* three days after queen removal [10].

This result, based on a simple comparison of time scales and the constitutive expression of queen genes in the absence of interactions, is in seeming contradiction to the experimentally observed stability of *Polistes* societies over much longer time scales. This raises the question of how the social structure is stabilised. In the remainder of this section we will ask whether the observed reduction of gene expression variance in workers (Fig. 4b of the main text) provides a mechanism for stabilising the society in the long term.

We begin by considering the probability distribution of queen gene expression values, $P(r)$, in workers. In a population composed of N workers, the probability of observing

a new queen is equal to the probability that at least one worker up regulates the queen genes. Assuming that the queen gene expression value workers is distributed according to $P(r)$, the probability of observing a new queen is equal to the probability of finding an insect with queen gene expression larger or equal to one,

$$p = \int_1^\infty P(r)dr\,, \tag{34}$$

Consistent with our previous calculation, Equation (33), the persistence time of the society then is

$$\tau(\sigma) = \omega^{-1}\left[\frac{1}{1-(1-p)^N} + R\right]\,. \tag{35}$$

The variance of $P(r)$, σ^2 is determined by an interplay between queen gene expression and queen interactions. Our main conclusions from this analysis are independent of the details of these processes. We here provide two examples to illustrate our results. If subdominant interactions with the queen are statistically independent and happen at a rate independent of queen gene expression levels in workers, then queen gene expression levels in workers follow an exponential distribution, $P(r) = \exp(-\omega r/\mu)$, with μ and ω being the rate of queen gene expression and interactions, respectively. With the variance given by $\sigma^2 = (\mu/\omega)^2$ we obtain $p = \exp(-1/\sigma)$ and a persistence time of the society of

$$\tau = \omega^{-1}\left[\frac{1}{1-(1-e^{-1/\sigma})^N} + R\right]\,. \tag{36}$$

In a more realistic setting corresponding to our model the rate of repressive interactions of a worker is proportional to its level of queen gene expression. In this case, in analogy to stochastic growth processes with resetting, queen gene expression levels

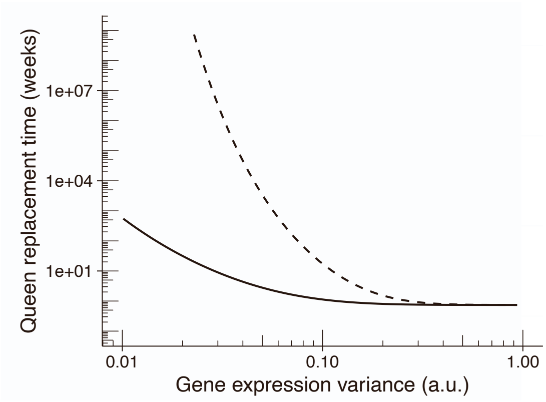

Figure 5: Lifetime of the society as a function of the population variance for normally (dashed line) and exponentially (solid line) distributed populations for values of the parameters $\omega = 3.7$ interactions/day, $R = 5.55$ and $N = 20$.

of workers follow a truncated normal distribution [11],

$$P(r) = \sqrt{\frac{2\omega^2}{\pi\mu^2}} e^{-\frac{r^2\omega^2}{2\mu^2}} . \tag{37}$$

With a variance $\sigma^2 = \mu^2/\omega^2$ we find in this case that

$$\tau = \omega^{-1} \left(\frac{1}{1 - (1 - \mathrm{Erfc}\left[(2\sigma^2)^{-1/2}\right])^N} + R \right) , \tag{38}$$

where $\mathrm{Erfc}(x) = 1 - \mathrm{Erf}(x)$ is the complementary error function.

Taken together, variations in the variance of queen gene expression levels across workers have a strong, orders of magnitude effect on the stability of the society as a whole Figure 5. This holds qualitatively true independently of the details of the molecular of population level processes. These results, together with our finding that gene body methylation is associated with a decrease in gene expression variance in *Polistes* (Fig. 4b of the main text) shows that DNA methylation plays a significant role in stabilising the society in the long term.

7 Prediction of experimental data

Having derived the model describing the evolution of the joint probability of gene expression and queen gene repressors in Equation (9), we now set out to fix its parameters and predict the experimental data. To derive a mathematical description that is less dependent on parameters describing poorly understood molecular processes, such as the dynamics of queen gene repressors, we now derive an effective description of the time evolution of the marginal distribution of ovary sizes. To this end, we will derive a description based on ovary growth starting from the model derived above. As ovary sizes are a mere downstream effect of the queen gene expression dynamics (see below), such an effective description should be structurally similar to the master equation describing the time evolution of the marginal distribution of queen gene expression levels, Equation (13), if the time scales are chosen appropriately.

7.1 Marginal distribution of ovary sizes

In order to predict the time evolution of the probability of observing ovaries of size $\{o_k\}$ at time t, $P(\{o_k\}, t)$, we first note that many of the queen genes have functions related to reproduction or are directly responsible for ovary development, such that the instantaneous rate of ovary growth is given by a function that depends on the expression level of queen genes, $g(n_k)$. The function $g(n)$ summarises a cascade of molecular pathways which are not understood in detail. We here make the simplest possible assumption about the functional form of $g(n)$, namely that it depends linearly on gene expression levels and that ovaries grow if the gene expression level exceeds a threshold, n_0, and shrink otherwise. Therefore, the rate of ovary growth takes the form $g(n) \propto n - n_0$. As eggs are laid once they have reached a mature size, we impose a reflective boundary condition on o_i at a size o_0 which we set to be the maximum ovary size observed in the experiment, $o_0 = 2.5\,\text{mm}$. With this, switching back to dimensional quantities, the time evolution of the conditional probability of ovary sizes

follows a master equation of the form

$$\frac{d}{dt}P(\{o_k\},t|\{n_k,q_k\}) = g(n_k)\theta(n_k-n_0)\left[P(\{o_k-1\},t|\{n_k,q_k\}) - P(\{o_k\},t|\{n_k,q_k\})\right] \tag{39}$$

$$- g(n_k)\theta(n_0-n_k)\left[P(\{o_k+1\},t|\{n_k,q_k\}) - P(\{o_k\},t|\{n_k,q_k\})\right] .$$

As, in this model, the expression level of queen genes is independent of ovary size, the joint probability $P(\{n_k,o_k\},t)$ factorizes as $P(\{n_k,o_k\},t) = P(\{o_k\}|\{n_k\},t)P(\{n_k\},t)$. Therefore, the dynamics of the joint probability of $\{n_k,o_k,q_k\}$ are described by

$$\begin{aligned}\frac{d}{dt}P(\{n_k,o_k,q_k\}) = \sum_{i=1}^{N+1}\Big\{&\mu(1-q_i)\left[P(\{n_i-1,o_i,q_i\}) - P(\{n_i,o_i,q_i\})\right] \\ &+ \delta\left[(n_i+1)P(\{n_i+1,o_i,q_i\}) - n_iP(\{n_i,o_i,q_i\})\right] \\ &+ g(n_i)\theta(n_i-n_0)\left[P(\{n_i,o_i-1,q_i\}) - P(\{n_i,o_i,q_i\})\right] \\ &- g(n_i)\theta(n_0-n_i)\left[P(\{n_i,o_i+1,q_i\}) - P(\{n_i,o_i,q_i\})\right] \\ &+ \Gamma(t_i^{\mathrm{int}})P(\{n_i,o_i,1\})(1-2q_i) \\ &+ \omega\sum_{j\neq i}K(n_i,n_j)P(\{n_i,o_i,0\})(2q_i-1)\Big\} ,\end{aligned} \tag{40}$$

with $g(n)$ denoting the rate of ovary growth for a gene expression level n.

With the aim of comparing our simulation results to the experimental ovary dissection data we first integrate out gene expression, yielding an equation describing the evolution of ovary sizes. If the persistence time of the repressive effect of interactions plus the typical production time of gene products is smaller than the typical time between two interactions, individuals alternate periods of growing and shrinking of their ovaries with the duration of these periods determined by the ratio between the typical interaction and persistence times plus the queen gene production times. Taking this limit, the ovary growth rate only depends on whether queen gene expression is above

or below the threshold n_0. For that purpose we define a random variable,

$$s_i = \Theta(n_i - n_0)\,, \tag{41}$$

such that the ovary growth rate is proportional to $2s_i - 1$.

The time evolution of the random variable s_i is linked to the dynamics of n_i. This gives rise to an explicit time delay in the ovary equation. Specifically, following an interaction, an individual with $s_i = 1$ needs a time t_{off} to degrade enough gene products and activate the pathways responsible for flipping the ovary growth state, $s_i = 0$. On the other hand, if an individual with $s_i = 0$ does not engage in a subdominant interaction during a time $t_{\text{on}} \approx t_{\text{off}} + t_{\text{per}}$, given by the sum of the persistence time of queen gene repressors and the time needed to express queen genes beyond a level n_0 and activate pathways related to reproduction, it will again flip the ovary growth state, $s_i = 1$.

In the long time limit the ovary size of a given individual is determined by the sign of the diference $T_{s_i=1} - T_{s_i=0}$ where $T_{s_i=j} = \int_0^t dt'\theta(s_i - j)$ is the time that the ovary growth rate spends in the state j. In this limit, the instantaneous value of gene expression r at time t is a good indicator of the sign of $T_{s_i=1} - T_{s_i=0}$, and hence of ovary growth. Taking this into account the interaction kernel can be expressed in terms of ovary size and we obtain the effective master equation governing the evolution of the

marginal distribution of ovary sizes,

$$\frac{d}{dt}P(\{o_k, s_k\}) = \sum_{i=1}^{N+1}\Big\{g(s_i)\left[P(\{o_i - 1, s_i\}) - P(\{o_i, s_i\})\right] + \delta(t_i^{\mathrm{int}} - t_{\mathrm{off}})P(\{o_i, 1\})(1 - 2s_i) + \delta(t_i^{\mathrm{int}} - t_{\mathrm{on}})P(\{o_i, 0\})(2s_i - 1)\Big\}, \tag{42}$$

where t_i^{int} is, as before, the time elapsed since the last subdominant interaction of individual i. Equation (42) provides a description of the system at the ovary level that retains the foremost characteristics present in the experimental data, i.e. the presence of multiple queens in the nest shortly after reprogramming and the posterior relaxation towards an analogous state to the control, as demonstrated by the results in Figure 2F of the main text. The existence of explicit time delays t_{on} and t_{off} is responsible for the transient observation of multiple queens during reprogramming. Specifically, such an overshoot arises if the value of t_{off} is of similar magnitude as the time scale associated with ovary growth.

7.2 Experimental parameters

The model defined in Equation (42) includes several parameters. In this section we provide justifications for the parameter values used to predict the experimental observables in Supplementary Fig. 2f-h of the main text. In the derivation of the model, Equation (42), we did not assume any non-linearities unless supported by experimental data. Such non-linearities, for example in the relation between ovary growth and gene expression, naturally exist in any biological system. Therefore, we have to exercise some caution in interpreting these parameters in literal biological terms. We still expect, however, that the order of magnitude of parameters is not altered by unknown

Parameter	Value	Justification
ω (Interaction rate)	3.7 day^{-1}	Measurements from video recordings presented in Supplementary Fig. 4A of the main text.
τ_{off} (Typical time between an interaction and changes in ovary growth)	2.5 days	We estimated this parameter based on the observation of 2-3 egg layers in the early commitment phase.
o_r (Ovary growth rate)	$0.25\,\text{mm}\,\text{day}^{-1}$	The first egg layer is observed 6 days after queen removal with a size of mature eggs of 1.5 mm.

Table 1: Summary of parameter values used for predicting experimental data.

non-linearities and can be estimated by independent observations from experiments or the literature. Parameter values are summarised in Table 1.

The time evolution of the global activity in a nest (Supplementary Fig. 2h) is determined by a component which is independent of the fighting interactions involved in the regulation of the reprogramming process and a component reflecting these interactions. Only the latter component is reflected in the model. We note that this component is proportional to the total interaction rate. From Equation (42) we find that this interaction rate is

$$\sum_{ij} o_i o_j f(o_i) f(o_j) = \left(\sum_i o_i f(o_i) \right) \left(\sum_j o_j f(o_j) \right) = \langle o_i \rangle^2 .$$

In Supplementary Fig. 2h, to take into account the different contributions to the global activity mentioned above, an we added an offset value to the theoretical prediction such that the empirical and theoretical values matched in the control phase. Then, activity levels were rescaled such that the maximum of both curves matched.

8 Numerical simulations

We performed kinetic Monte Carlo simulations following Gillespie's algorithm to obtain approximate solutions to master equation for a population of N individuals [12]. Out of the different possible processes - production of a new gene product, degradation of gene products, interactions, degradation of the queen gene repressors or ovary development - one was randomly selected with probability proportional to the overall rate of the respective process (in vector form)

$$\left(\mu, \delta n_i, \alpha K(n_i, n_j), \delta(t_{int}^i - t_{per}), g(n_i)\right) \ . \tag{43}$$

Once a process has been selected the state of the system is updated depending on the selected reaction and finally, the simulation time is advanced an amount Δt drawn from an exponential distribution of parameter λ given by the inverse sum of the overall rates,

$$\lambda = \left\{ \sum_i \left(\mu + \delta n_i + \sum_j K(n_i, n_j) + \delta(t_{int}^i - t_{per}) + g(n_i) \right) \right\}^{-1} \ . \tag{44}$$

Unless specified otherwise the following parameters were used for all the simulations $\mu = 500$, $\delta = 1$, $n_0 = 250$, $\lambda = 10$, $t_{per} = 1$. The ovary growth rate was chosen so that ovaries were mature in 6 days, as observed experimentally.

All simulations were implemented in Julia and the source code is available upon request to the authors.

References

1. Assaf, M. & Meerson, B. WKB Theory of Large Deviations in Stochastic Populations. *J. Phys. A: Math. Theor.* **50,** 263001 (2017).

2. Hamilton, A., Shpigler, H., Bloch, G., Wheeler, D. & Robinson, G. in *Hormones, Brain and Behavior* 421–451 (Elsevier, 2017).

3. Roeseler, P.-F., Roeseler, I., Strambi, A. & Augier, R. Influence of Insect Hormones on the Establishment of Dominance Hierarchies among Foundresses of the Paper Wasp, Polistes Gallicus. *Behav Ecol Sociobiol* **15,** 133–142 (1984).

4. Oi, C. A. *et al.* Do Primitively Eusocial Wasps Use Queen Pheromones to Regulate Reproduction? A Case Study of the Paper Wasp Polistes Satan. *Front. Ecol. Evol.* **7,** 199 (2019).

5. Gardiner, C. W. *Handbook of Stochastic Methods for Physics, Chemistry, and the Natural Sciences* 3rd ed (Springer-Verlag, 2004).

6. Bauer, M., Knebel, J., Lechner, M., Pickl, P. & Frey, E. Ecological Feedback in Quorum-Sensing Microbial Populations Can Induce Heterogeneous Production of Autoinducers. *eLife* **6,** 1–38 (2017).

7. Jörg, D. J., Kitadate, Y., Yoshida, S. & Simons, B. D. Competition for Stem Cell Fate Determinants as a Mechanism for Tissue Homeostasis. arXiv: `1901.03903 [physics, q-bio]` (2019).

8. Cross, M. & Greenside, H. *Pattern formation and dynamics in nonequilibrium systems* (Cambridge University Press, 2009).

9. Rulands, S., Klünder, B. & Frey, E. Stability of Localized Wave Fronts in Bistable Systems. *Phys. Rev. Lett.* **110,** 038102 (2013).

10. Taylor, B. A., Cini, A., Wyatt, C. D. R., Reuter, M. & Sumner, S. The Molecular Basis of Socially Mediated Phenotypic Plasticity in a Eusocial Paper Wasp. *Nature Communications* **12,** 775 (2021).

11. Biró, T. & Néda, Z. Unidirectional Random Growth with Resetting. *Physica A: Statistical Mechanics and its Applications* **499,** 335–361 (2018).

12. Gillespie, D. T. Exact Stochastic Simulation of Coupled Chemical Reactions. *J. Phys. Chem.* **81,** 2340–2361 (1977).
